# Mechanistic studies of the palladium-catalyzed S,O-ligand promoted C–H olefination of aromatic compounds†

**DOI:** 10.1039/d2sc06840k

**Published:** 2023-02-16

**Authors:** Kananat Naksomboon, Enrique Gómez-Bengoa, Jaya Mehara, Jana Roithová, Edwin Otten, M. Ángeles Fernández-Ibáñez

**Affiliations:** a Van't Hoff Institute for Molecular Sciences, University of Amsterdam Science Park 904 1098 XH Amsterdam The Netherlands m.a.fernandezibanez@uva.nl; b Department of Organic Chemistry I, Universidad País Vasco, UPV/EHU Apdo. 1072 20080 San Sebastian Spain; c Institute for Molecules and Materials, Radboud University Heyendaalseweg 135 6525 AJ Nijmegen The Netherlands; d Stratingh Institute for Chemistry, University of Groningen Nijenborgh 4 9747 AG Groningen The Netherlands

## Abstract

Pd-catalyzed C–H functionalization reactions of non-directed substrates have recently emerged as an attractive alternative to the use of directing groups. Key to the success of these transformations has been the discovery of new ligands capable of increasing both the reactivity of the inert C–H bond and the selectivity of the process. Among them, a new type of S,O-ligand has been shown to be highly efficient in promoting a variety of Pd-catalyzed C–H olefination reactions of non-directed arenes. Despite the success of this type of S,O-ligand, its role in the C–H functionalization processes is unknown. Herein, we describe a detailed mechanistic study focused on elucidating the role of the S,O-ligand in the Pd-catalyzed C–H olefination of non-directed arenes. For this purpose, several mechanistic tools, including isolation and characterization of reactive intermediates, NMR and kinetic studies, isotope effects and DFT calculations have been employed. The data from these experiments suggest that the C–H activation is the rate-determining step in both cases with and without the S,O-ligand. Furthermore, the results indicate that the S,O-ligand triggers the formation of more reactive Pd cationic species, which explains the observed acceleration of the reaction. Together, these studies shed light on the role of the S,O-ligand in promoting Pd-catalyzed C–H functionalization reactions.

## Abstract 2

A mechanistic study of the role of the S,O-ligand in promoting palladium catalyzed C–H activation of arenes is described. The data indicate that the S,O-ligand triggers the formation of more reactive palladium cationic species.

## Graphical abstract

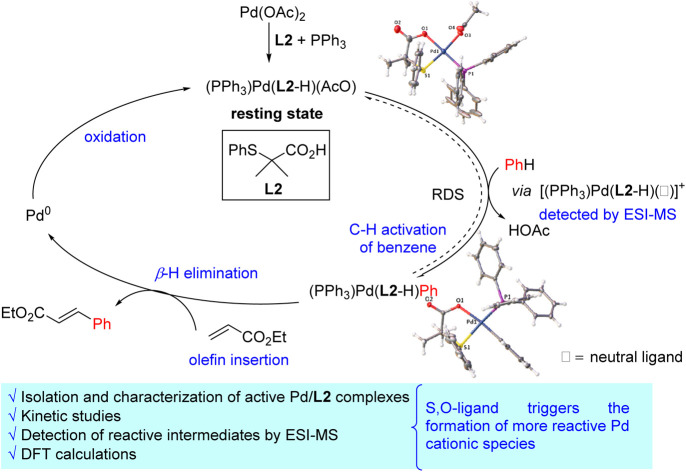

## Introduction

Over the past 20 years, transition-metal-catalyzed C–H functionalization reactions have become a powerful synthetic tool in organic synthesis due to their high atom efficiency compared with traditional methods.^1^ The majority of the reported methodologies rely on the use of directing groups to increase the reactivity and selectivity of the process.^2^ The C–H functionalization of non-directed substrates offers the possibility for orthogonal selectivity as well as broader substrate scope beyond those bearing directing groups. However, these processes suffer from low yields and low levels of site-selectivity even using high catalyst loading, an excess of the arene and harsh reaction conditions. It was not until recently that the discovery of new ligands has allowed to develop efficient transformations.^3^

**Scheme 1:**
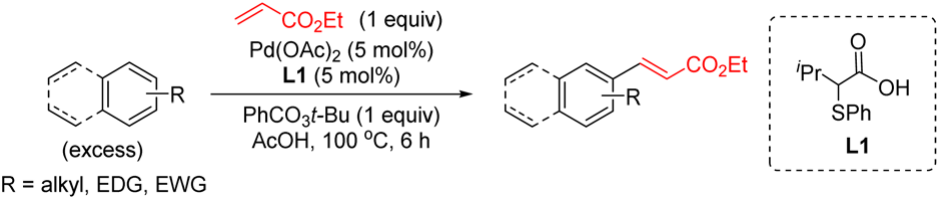
Pd/S,O-ligand-catalyzed C–H olefination of non-directed arenes.

Mono-protected amino acids ligands (MPAA),^4^ pyridine-based ligands^5^ or the combinations of both^6^ as well as bis(carboxylate) anions^7^ have been identified as effective ligands for these transformations. Additionally, we have discovered a new type of S,O-ligand, namely thioether carboxylic acid, that enables palladium-catalyzed C–H olefination of simple arenes, thiophenes, anisole, and aniline derivatives (Scheme 1).^8^ The new catalytic system based on Pd/S,O-ligand is also compatible with the introduction of other functionalities^8*i*,*j*^ and has inspired other research groups to incorporate the S,O-ligand into a heterogeneous catalyst.^9^

A unique feature of the Pd/S,O-ligand catalytic system is its high catalytic activity, enabling the functionalization of substrates that are unreactive using other catalysts. Despite the potential applications of the Pd/S,O-ligand catalyst in C–H functionalization reactions, the mechanistic role of the S,O-ligand is unknown. A detailed understanding of the role of this type of ligands in the reaction mechanism will be essential for future developments.

Herein, we describe a detailed mechanistic investigation of the role of the S,O-ligand in Pd-catalyzed C–H functionalization reactions of aromatic compounds. We have isolated and characterized by X-ray diffraction analysis several complexes before and after C–H activation and evaluated their catalytic activities. NMR and kinetic studies reveal that the C–H activation is rate limiting. Moreover, these studies suggest that the S,O-ligand triggers the formation of Pd cationic species. Additionally, a cationic Pd-complex was detected by ESI-MS and its reactivity in C–H activation processes was studied. DFT calculations corroborate the feasibility of the cationic pathway in the C–H activation of benzene as well as reveal the higher reactivity of cationic complexes in these processes. These investigations provide insights into the role of the S,O-ligand in promoting Pd-catalyzed C–H functionalization reactions. We expect that our findings will not only serve to guide future ligand development in the growing field of non-directed C–H functionalization reactions but also in further applications of this type of ligands in metal-catalysis.

## Results and discussion

### Identification of active Pd complexes

**Scheme 2:**
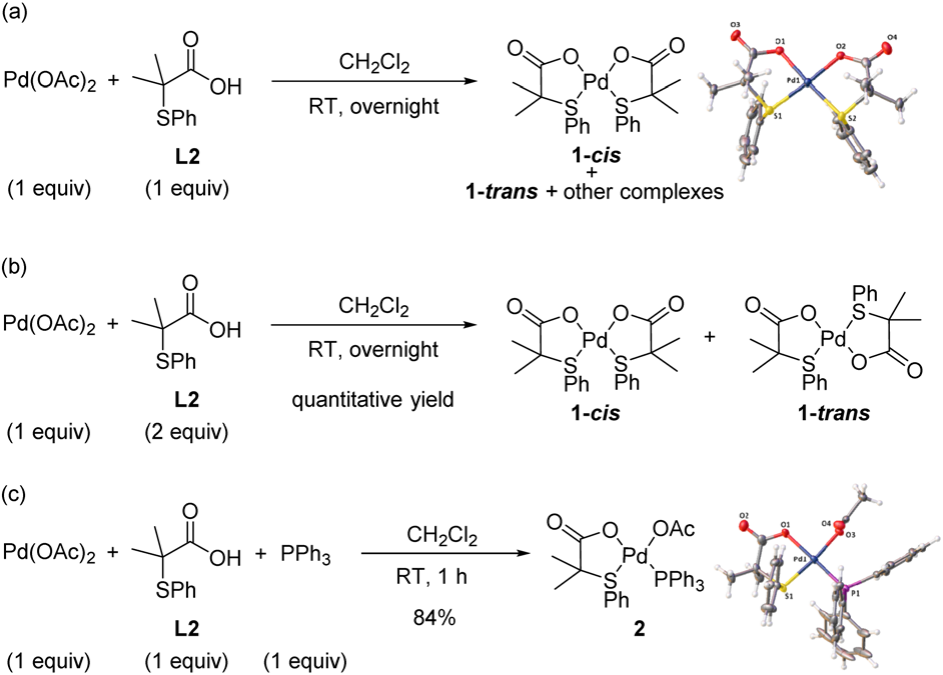
Synthesis of Pd/S,O-ligand complexes.

Initially, we concentrated our efforts on the identification of Pd complexes bearing an S,O-ligand. To facilitate mechanistic studies, we performed our experiments using the 2-methyl-2-(phenylthio)propanoic acid ligand (L2) that shows similar performance in the C–H olefination of simple arenes as the 3-methyl-2-(phenylthio)butanoic acid (L1) previously used.^8*a*^ With this purpose, we reacted Pd(OAc)_2_ with L2 in a 1 : 1 ratio in CH_2_Cl_2_ at room temperature overnight (Scheme 2a). From the ^1^H NMR spectrum of the crude reaction mixture, several complexes were detected. Fortunately, we were able to unequivocally characterize by X-ray analysis the major Pd complex 1-*cis* bearing two S,O-ligands in a *cis* geometry.^8*a*,10^ When the reaction was performed using 2 equiv. of ligand L2, Pd-complexes 1-*cis* and 1-*trans* were obtained in quantitative yield in a 2 : 1 ratio, respectively (Scheme 2b). In order to isolate the Pd complex with only one S,O-ligand attached, we performed the reaction in the presence of PPh_3_ using a 1 : 1 : 1 ratio of Pd : L2 : PPh_3_ in CH_2_Cl_2_. To our delight, complex 2 bearing one S,O-ligand, one PPh_3_ and one acetate ligand was isolated in 84% yield and fully characterized by X-ray analysis (Scheme 2c).^11^

**Fig. 1:**
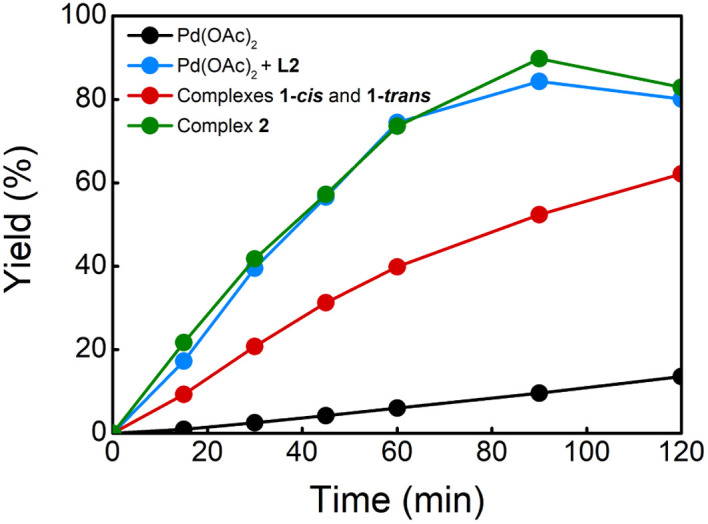
Kinetic profiles of C–H olefination of benzene.

With these complexes in hand, we performed several experiments to evaluate their catalytic activities (Table 1). We executed the reaction of benzene with ethyl acrylate under the optimal reaction conditions with and without ligand L2 (entries 1 and 2).^8*a*^ The reaction without ligand provided, after 2 h, the olefinated product in 21% yield and in the presence of L2, the olefinated products 3 were obtained in 78% yield. When we performed the same reaction using 5 mol% of complex 1 (*cis* and *trans*) instead of Pd(OAc)_2_, the olefinated product was obtained in 53% yield (entry 3), indicating that complex 1 (*cis* and *trans*) might accelerate the C–H olefination reaction. To see the effect of PPh_3_ in the reaction, we tested the reaction using 5 mol% of a catalyst based on a 1 : 1 : 1 ratio of Pd : L2 : PPh_3_. This reaction provided the desired products in 79% yield which is comparable to the result of the reaction without PPh_3_ (entries 2 and 4). To our delight, the reaction using 5 mol% of complex 2 gave products 3 in 77% yield (entry 5), suggesting that this complex is involved in the catalytic reaction in the presence of PPh_3_. Additionally, the kinetic profiles of the reactions using different Pd catalysts were also performed (Fig. 1). The reactions using a 1 : 1 ratio of Pd : L2 and complex 2 as catalyst provided comparable curves suggesting that PPh_3_ does not have a critical role in the reaction. The reaction using complex 1 showed an increase in the reaction rate compared with the reaction without ligand. However, the kinetic profile shows that the reaction is slower than when complex 2 or a 1 : 1 ratio of Pd : L2 is used.

**Table 1:** Reactivity of Pd catalyst in the C–H olefination of benzene^a^

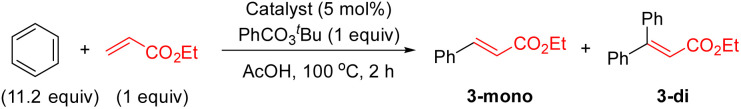		
Entry	Catalyst	NMR yield (3-mono + 3-di)
1	Pd(OAc)_2_	21% (21% + 0%)
2	Pd(OAc)_2_ + ligand L2 (1 : 1)	78% (74% + 4%)
3	Complexes 1-*cis* and 1-*trans*	53% (53% + 0%)
4	Pd(OAc)_2_ + ligand L2 + PPh3 (1 : 1 : 1)	79% (78% + 1%)
5	Complex 2	77% (75% + 2%)

a Yield was determined by ^1^H NMR analysis of the crude mixture using CH_2_Br_2_ as internal standard.

**Scheme 3:**
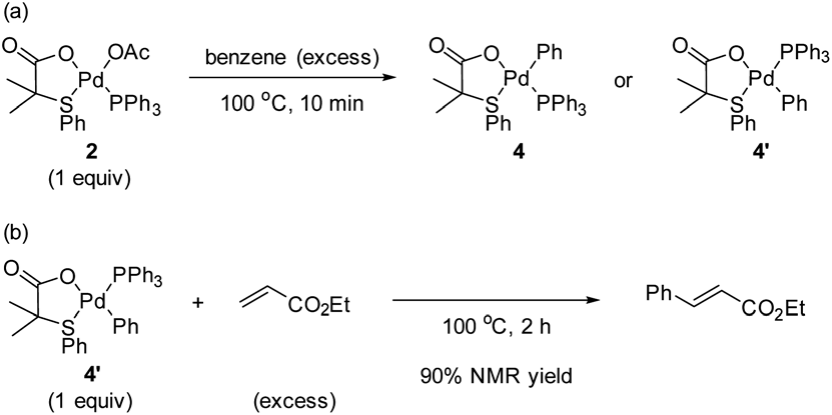
Reaction of (a) Pd complex 2 with benzene and (b) Pd complex 4′ with ethyl acrylate.

With the intention to identify a Pd complex after the C–H activation step, we carried out the reaction of complex 2 with benzene in a pressure tube at 100 °C. The reaction after 2 h provided a complex mixture where complex 4/4′ could not be identified. Therefore, we repeated the reaction and stopped after 10 min when the formation of Pd black was observed (Scheme 3a). From the ^1^H NMR spectrum of the reaction mixture, we tentatively assigned the peaks from 6.4–6.6 ppm to the formation of complexes 4 and/or 4′, which come after the C–H activation step, along with many other peaks that we could not identify. From the ^31^P NMR spectrum, mainly three equally intense peaks which belong to triphenylphosphine oxide, complexes 2 and 4 (or 4′) were observed. Unfortunately, from the reaction mixture, we were not able to isolate complex 4 (or 4′).

To confirm the formation of complex 4 (or 4′), we decided to synthesize 4′*via* a different synthetic route following a literature procedure.^12^ The structure of complex 4′ was confirmed by X-ray analysis that crystallizes in a non-centrosymmetric space group (see ESI†).^13^ Then, we compared the ^1^H and ^31^P NMR spectra of 4′ with the one obtained from the reaction of 2 with benzene. We found that both spectra match, confirming that 4′ was formed in the reaction of complex 2 with benzene. Next, the reaction of complex 4′ with an excess of ethyl acrylate furnished the olefinated product in 90% ^1^H NMR yield (Scheme 3b). From these results, we suggest that complexes 2 and 4′ are active catalysts in the reaction.

### Identification of Pd complexes during the catalytic reaction

**Fig. 2:**
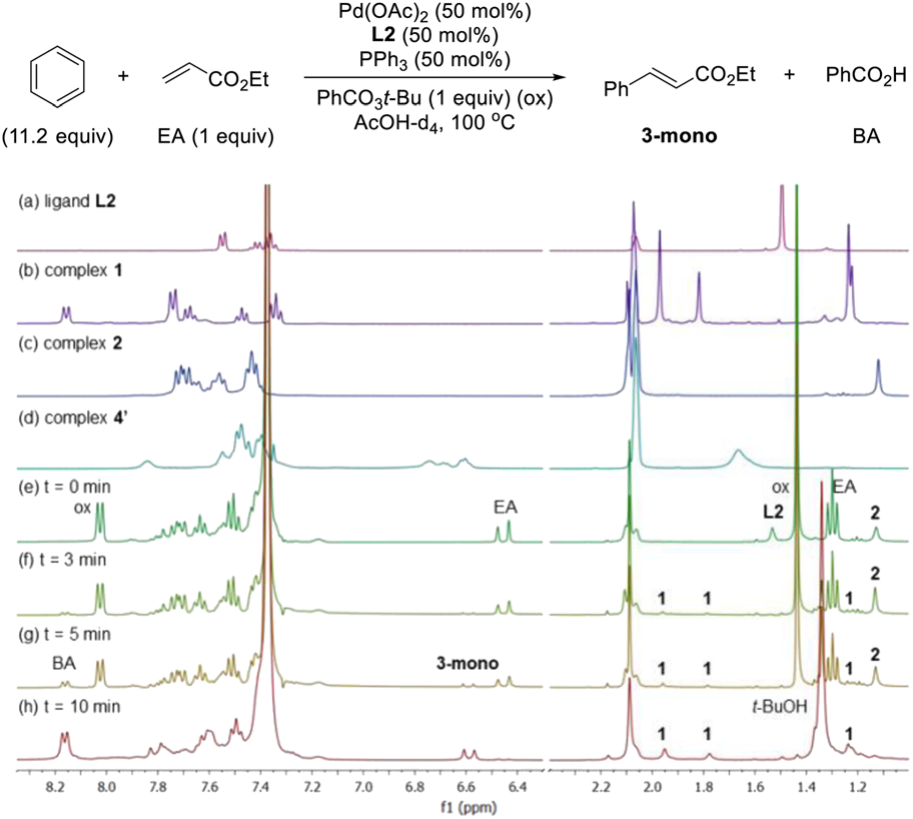
^1^H NMR spectra of (a) ligand L2, (b) complexes 1-*cis* and 1-*trans*, (c) complex 2, (d) complex 4′ in AcOH-d_4_. C–H Olefination of benzene and ethyl acrylate using a 1 : 1 : 1 ratio of Pd : L2 : PPh_3_ as catalyst in AcOH-d_4_ monitored by ^1^H NMR spectroscopy at (e) 0 min, (f) 3 min, (g) 5 min and (h) 10 min. ox = oxidant, EA = ethyl acrylate, BA = benzoic acid.

**Fig. 3:**
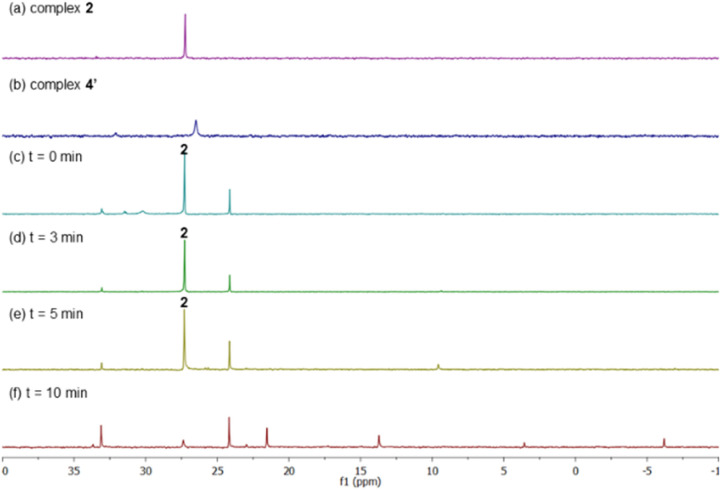
^31^P NMR spectra of (a) complex 2 and (b) complex 4′ in AcOH-d_4_. C–H Olefination of benzene and ethyl acrylate using a 1 : 1 : 1 ratio of Pd : L2 : PPh_3_ as catalyst in AcOH-d_4_ monitored by ^31^P NMR spectroscopy at (c) 0 min, (d) 3 min, (e) 5 min and (f) 10 min.

After having several Pd complexes characterized, we attempted to identify the complexes that were formed during the catalytic reaction. The reaction was performed in an NMR tube at 100 °C and was followed at different times by measuring the ^1^H and ^31^P NMR spectra at room temperature. We performed the reaction under standard conditions but using 50 mol% of the catalyst and deuterated acetic acid as a solvent. The comparison of ^1^H NMR spectra of ligand L2, Pd complexes 1, 2 and 4′ and the reaction at different times (0, 3, 5 and 10 min) are shown in Fig. 2. Before the reaction started (0 min), ligand L2 and complex 2 were clearly identified from their characteristic peaks of the methyl groups. After 3 and 5 min, we observed mainly complex 2 in the ^1^H NMR spectra. After 10 min, the reaction was completed and the signals of complex 2 were almost insignificant. After 3 min, we started to observe trace amounts of complex 1 and we did not detect any formation of complex 4′ during the reaction. The ^1^H NMR data were consistent with ^31^P NMR spectra as shown in Fig. 3. Mainly the peak at 27 ppm, which belongs to complex 2, was observed from 0 to 5 min and the intensity of this peak dramatically decreased after 10 min. The formation of complex 4′ (^31^P NMR at 26 ppm) was not detected during the reaction; however, other unidentified phosphorus species were formed. These results indicate that complex 2 is the resting state of the catalyst.

**Fig. 4:**
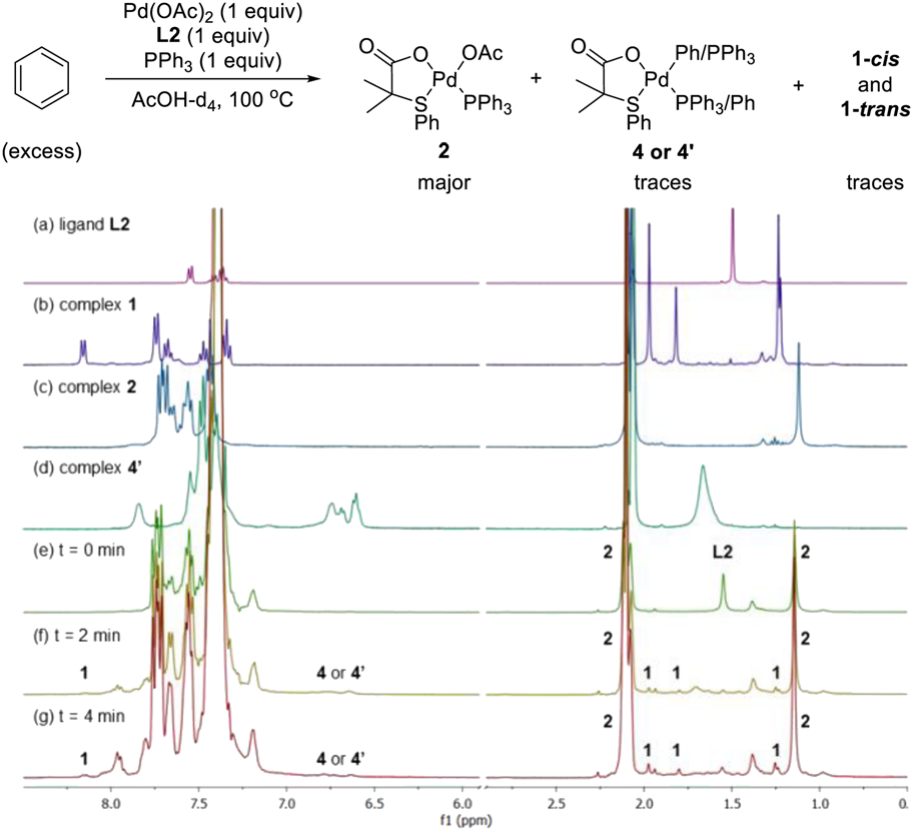
^1^H NMR spectra of (a) ligand L2, (b) complexes 1-*cis* and 1-*trans*, (c) complex 2 and (d) complex 4′ in AcOH-d_4_. The reaction of benzene with Pd(OAc)_2_, ligand L2 and PPh_3_ in AcOH-d_4_ monitored by ^1^H NMR spectroscopy at (e) 0 min, (f) 2 min and (g) 4 min.

**Fig. 5:**
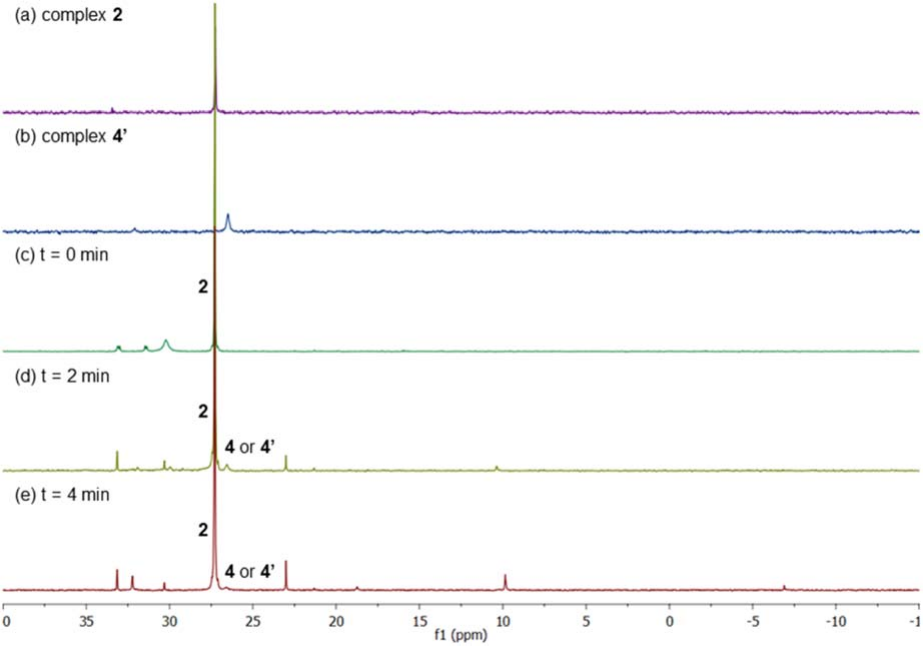
^31^P NMR spectra of (a) complex 2 and (b) complex 4′ in AcOH-d_4_. The reaction of benzene with Pd(OAc)_2_, ligand L2 and PPh_3_ in AcOH-d_4_ monitored by ^31^P NMR spectroscopy at (c) 0 min, (d) 2 min and (e) 4 min.

Next, we examined the reaction using benzene, Pd(OAc)_2_, ligand L2 and PPh_3_ in AcOD-d_4_ in the absence of ethyl acrylate in an NMR tube at 100 °C, recording the NMR data at the indicated time at room temperature. As shown in Fig. 4, at 0 min, ligand L2 and complex 2 were detected. After 2 and 4 min, complex 2 was the main complex observed, traces of complex 1 were detected and traces of complex 4′ were identified from the peaks around 6.5–7 ppm. ^31^P NMR data corroborated that complex 2 (27 ppm) was the main complex during the reaction and that traces of complex 4′ (26 ppm) were formed (Fig. 5).

## Kinetic investigations

### Order of the reaction

**Fig. 6:**
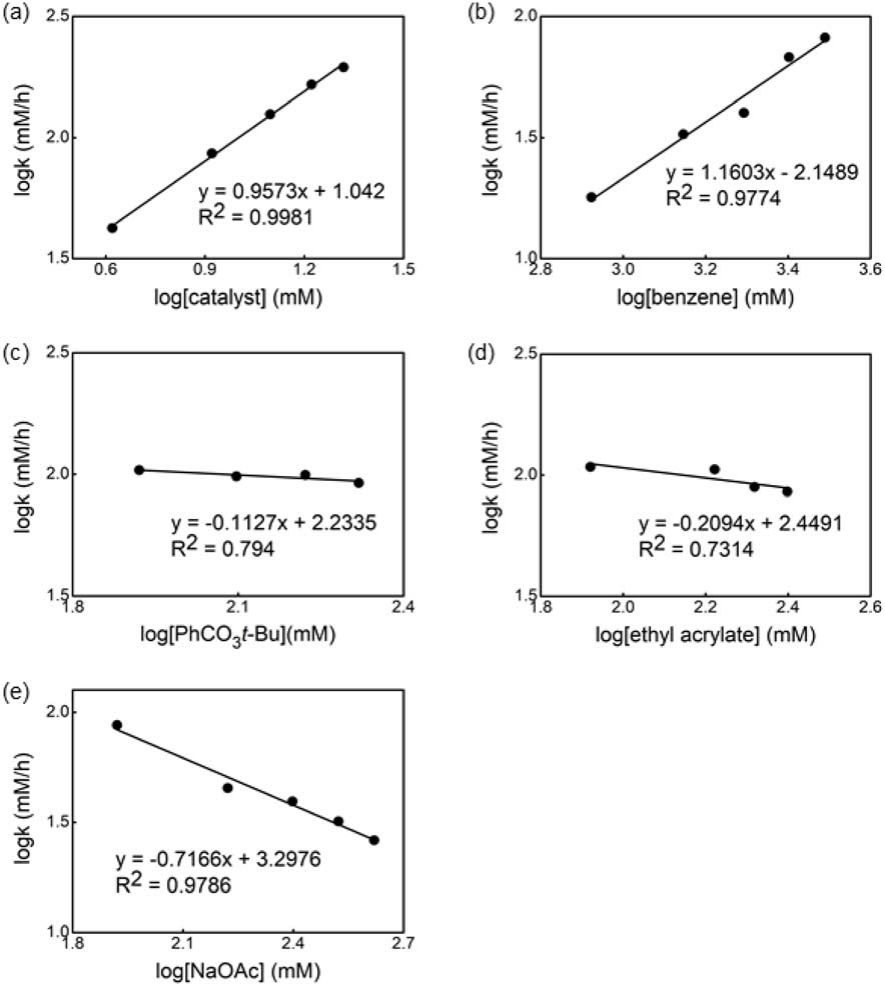
Dependence of the observed rate constant on the concentration of (a) catalyst, (b) benzene, (c) PhCO_3_*t*-Bu, (d) ethyl acrylate and (e) NaOAc for the reaction of Pd(OAc)_2_, ligand L1, *tert*-butyl peroxybenzoate, ethyl acrylate and benzene in AcOH at 100 °C.

The kinetic order of each reagent was determined in the reaction in the presence of ligand L1 by using the initial rate method.^14^Fig. 6 shows the plot of the logarithm of the reaction rate against the concentration of the reagent. A straight line with a slope of nearly one was measured for Pd-catalyst and benzene, revealing a first order in these reagents (Fig. 6a and b). Non-significant change in the reaction rate was observed using different concentrations of oxidant and olefin (Fig. 6c and d), indicating that the migratory insertion and oxidation of Pd(0) occur after the RDS. Nevertheless, the small negative fractional order in both cases can be explained by the formation of an off-cycle palladium complex bearing these reagents as a neutral ligand.^15^

Based on a previously reported observation of an inverse first-order dependence of the rate on [AcO^−^] in the C–H olefination of heteroarenes using a monodentate thioether ligand,^16^ we decided to determine the order of the reaction in NaOAc concentration and an inverse 0.7 order dependence was found (Fig. 6e).^17^ To explain the inverse order observed, we considered two different possibilities: (1) the association of acetate to form off-cycle [Pd(L1)(OAc)_2_]^−^ species and (2) a reversible dissociation of acetate from [LPd(L1)(OAc)] to form cationic palladium species. To distinguish between these two possibilities, we added an excess of NaOAc to a 1 to 1 mixture of L2 and Pd(OAc)_2_ in AcOD-d^4^ to investigate whether the formation of the off-cycle [Pd(S,O-L)(OAc)_2_]^−^ was the cause of the inverse order on acetate. After heating the mixture at 100 °C, we observed by ^1^H NMR that the relative intensity of peaks that belong to different Pd species changed but no major new Pd species were detected (see ESI†). Therefore, the observed inverse order with respect to NaOAc is not the result of the formation of off-cycle [Pd(S,O-L)(OAc)_2_]^−^ species. Taking into account these results, it seems reasonable to propose that more reactive cationic palladium species are formed during the reaction and prior the RDS.^16,18^

**Fig. 7:**
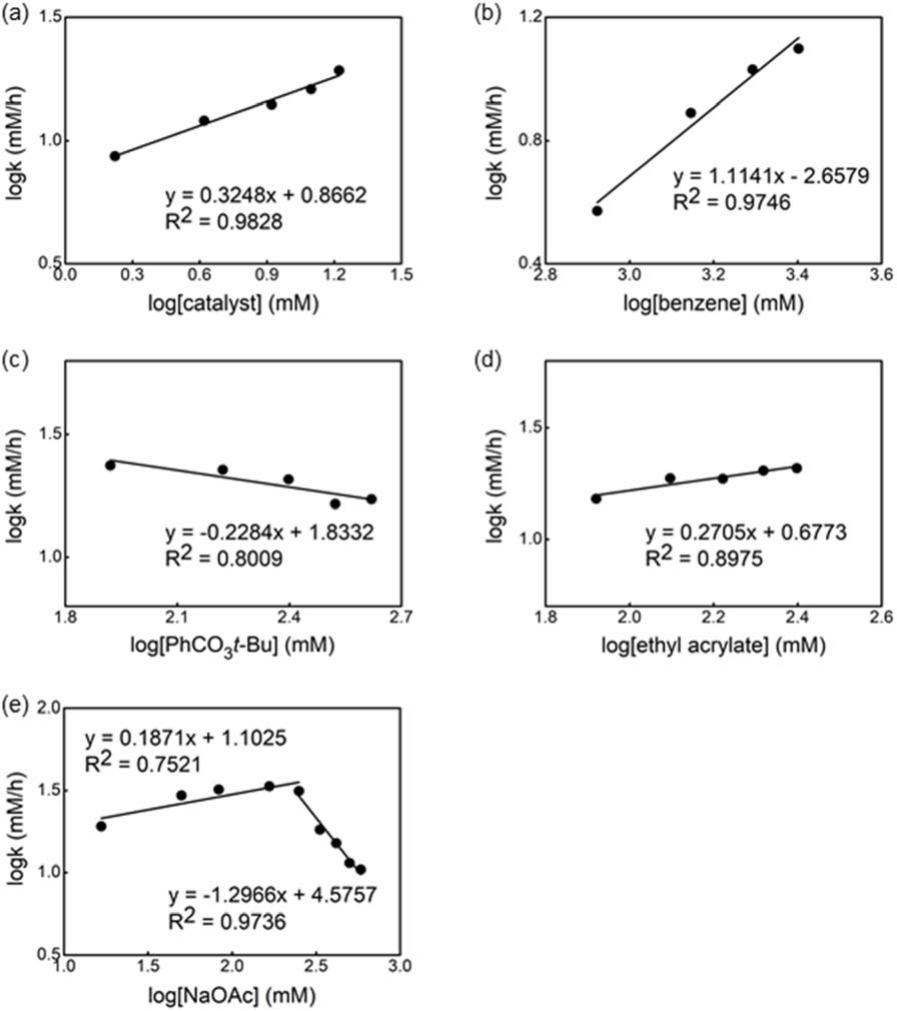
Dependence of the observed rate constant on the concentration of (a) catalyst, (b) benzene, (c) PhCO_3_*t*-Bu, (d) ethyl acrylate and (e) NaOAc for the reaction of Pd(OAc)_2_, *tert*-butyl peroxybenzoate, ethyl acrylate and benzene in AcOH at 100 °C.

Next, we investigated the order of each reagent in the reaction without ligand (Fig. 7). We observed a 0.3 order in the catalyst and the first order in the benzene concentration (Fig. 7a and b), suggesting a trimeric precatalyst and one benzene associated with the catalyst prior to the RDS.^19^ Again, near zero order in both oxidant and olefin were observed (Fig. 7c and d), indicating that olefin insertion and oxidation of Pd(0) occur after the RDS. Zero order in the acetate concentration is observed at lower concentrations of NaOAc and changed to the inverse 1.2 order dependence when the concentration of acetate was increased (Fig. 7e). We postulated that at higher concentrations of acetate the off-cycle [Pd(OAc)_3_]^−^ is formed in the reaction.

From these kinetic experiments, we suggest that the C–H activation is the RDS in both cases, with and without ligand and that the presence of the S,O-ligand triggers the formation of cationic species, which are expected to be more reactive.^18^ We also proposed that the reaction in the presence of the S,O-ligand occurs *via* catalytically active monomeric species which are the main species in solution.

### KIE and H/D exchange experiments

**Scheme 4:**
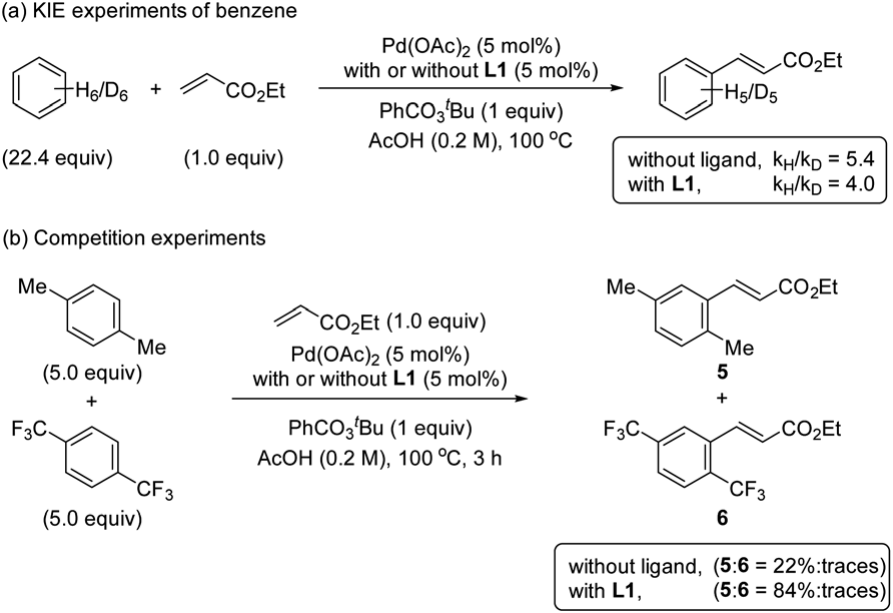
Mechanistic studies (a) KIE experiments of benzene and (b) competition experiments.

The KIE values were examined by performing reactions with benzene and benzene-d_6_ in two different reaction flasks.^20^ The reaction in the presence of ligand L1 provided a *k*_H_/*k*_D_ ratio of 4.0 and in the absence of ligand provided a *k*_H_/*k*_D_ ratio of 5.4 (Scheme 4a). The observed primary KIE corroborates together with the kinetic and NMR studies that the C–H bond cleavage is the RDS in the reaction with and without the S,O-ligand. Additionally, the reversibility of the C–H activation step by performing H/D scrambling experiments using AcOD-d_4_ as a deuterium source was evaluated (see ESI†). We used mesitylene as a diagnostic arene to facilitate the interpretation of the ^1^H NMR spectra. The reaction with ligand L1 provided the olefinated product and deuterated mesitylene in 17% after 6 h. In the reaction without ligand, by increasing the amount of the catalyst and the reaction time, the formation of 25% deuterated mesitylene was observed. Based on these experimental results we suggest that the C–H activation is *per se* reversible in both cases, with and without ligand.

### Competition experiments

To get more insights into the mechanism of the C–H activation step, we performed one-pot intermolecular competition experiments between an electron-rich arene, *p*-xylene, and an electron-poor arene, 1,4-bis(trifluoromethyl)benzene, with and without the ligand (Scheme 4b). We found that in both cases electron-rich arene reacted preferentially. These results together with the C–H bond cleavage being the RDS are consistent with an asynchronous concerted metalation deprotonation (CMD)^21,22^ or a base-assisted internal electrophilic-type substitution (BIES) mechanism.^23,24^

### Detection of reactive palladium complexes by ESI-MS and their reactivities in C–H activation processes

**Fig. 8:**
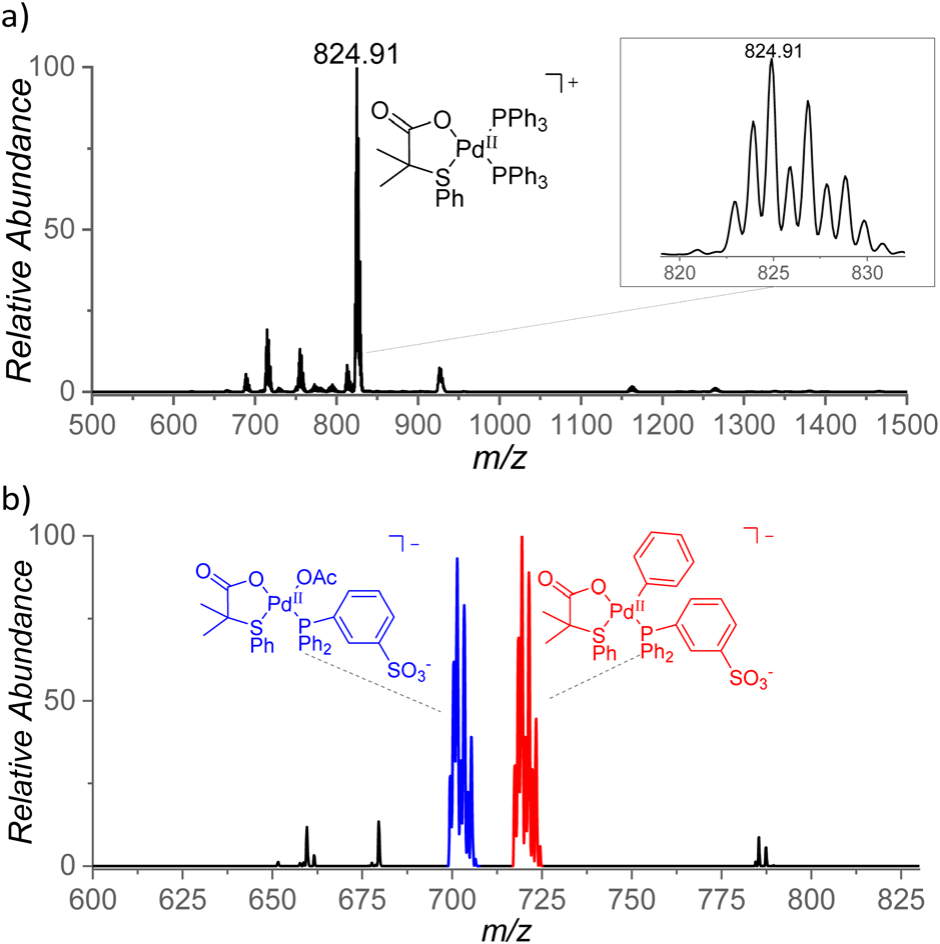
(a) ESI mass spectrum of a mixture of Pd(OAc)_2_, L2, and Ph_3_P in benzene and (b) ESI mass spectrum of Na[(PPh_2_Ph^SO3−^)Pd(L2)(OAc)] in benzene, heated for 5 min at 100 °C.

With the aim to identify cationic reactive intermediates in solution, we analyzed the system by electrospray ionization mass spectrometry (ESI-MS).^25^ Electrospray ionization of a solution of Pd(OAc)_2_ and S,O-ligand L2 in benzene led to the detection of larger palladium clusters with a combination of L2 and AcO^−^ ligands (Fig. S14 in the ESI†). The addition of PPh_3_ to the mixture resulted in the disappearance of the clusters and in the dominant formation of monomeric [(PPh_3_)_2_Pd(L2)]^+^ complex (*m*/*z* 825, Fig. 8a).

The key intermediates [(PPh_3_)Pd(L2)(Ph)] are neutral and thus not detectable by ESI-MS; therefore, we performed the experiments also with the negatively charged ligand PPh_2_Ph^SO3−^ (Ph^SO3−^ = 3-phenylsulfonate). The addition of the remote charge to the phosphine ligand allowed us to detect [(PPh_2_Ph^SO3−^)Pd(L2)(OAc)]^−^ and [(PPh_2_Ph^SO3−^)Pd(L2)_2_]^−^ ions (*m*/*z* 701 and *m*/*z* 836, respectively, Fig. S15†). The key intermediate [(PPh_2_Ph^SO3−^)Pd(L2)(Ph)]^−^ was detected only after heating of the [(PPh_2_Ph^SO3−^)Pd(L2)(OAc)]Na complex in benzene for 5 min at 100 °C (*m*/*z* 719, Fig. 8b) which points to a substantial energy barrier for the C–H activation reaction. The assignment of [(PPh_2_Ph^SO3−^)Pd(L2)(Ph)]^−^ was confirmed by repeating the experiment in deuterated benzene and by high-resolution measurements (Fig. S16 and S17†). The [(PPh_2_Ph^SO3−^)Pd(L2)(Ph)]^−^ can have two isomers (4/4′). We attempted to identify these isomers by ion mobility separation. The ion mobility profile is related to the shape of the ions and therefore it is expected to be different for the 4 and 4′ isomers. However, the ion mobility experiment indicated only one major isomer of the detected [(PPh_2_Ph^SO3−^)Pd(L2)(X)]^−^ complexes with X = Cl, OAc, and Ph. (Fig. S18†).

**Fig. 9:**
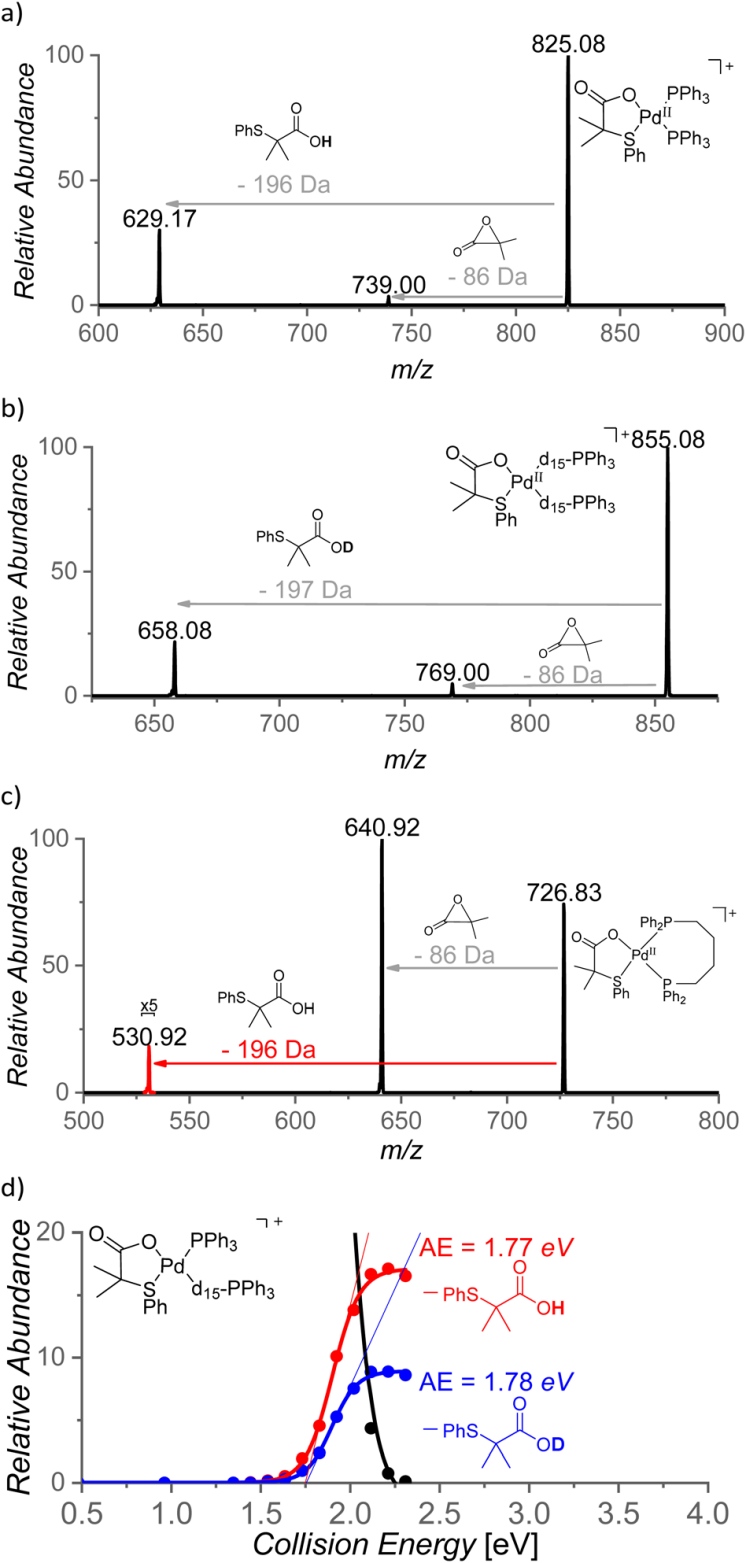
CID mass spectra of (a) [(PPh_3_)_2_Pd(L2)]^+^ (*m*/*z* = 825), (b) [(P(C_6_D_5_)_3_)_2_Pd(L2)]^+^ (*m*/*z* = 855), (c) [(dppb)Pd(L2)]^+^ (*m*/*z* = 727) and (d) energy resolved CID of mixed phosphine complex [(PPh_3_)(d_15_-PPh_3_)Pd(L2)]^+^ (*m*/*z* = 840).

The role of the S,O-ligand in the C–H activation process was further studied by intramolecular reactivity of the [(PPh_3_)_2_Pd(L2)]^+^ complex. This complex eliminates the neutral L2 ligand upon collisional activation (196 Da) suggesting that L2 engaged in C–H activation of a phenyl ring of the PPh_3_ ligand (Fig. 9a). This C–H activation process was confirmed by an experiment with [(PPh_3_-d_15_)_2_Pd(L2)]^+^ that indeed eliminated monodeuterated S,O-ligand L2D (197 Da) (Fig. 9b). The kinetic isotope effect of this process can be determined from the ratio of the L2-H : L2-D eliminations from mixed complex [(PPh_3_)(PPh_3_-d_15_)Pd(L2)]^+^ (*m*/*z* = 840) and it corresponds to KIE = ∼2. The energy demand of this process is 41 kcal mol^−1^ (∼1.8 eV, Fig. 9d).

The steric requirements for the C–H activation process can be explored by changing the bite angles between the ligands in [(PPh_3_)_2_Pd(L2)]^+^. Hence, the PPh_3_ ligands were exchanged by diphosphine ligands: dppm [1,1-bis(diphenylphosphino)methane], dppe [1,1-bis(diphenylphosphino)ethane], dppp [1,1-bis(diphenylphosphino)propane] and dppb [1,1-bis(diphenylphosphino)butane] (Fig. 9c). Complexes [(dppm)Pd(L2)]^+^ and [(dppe)Pd(L2)]^+^ do not show any C–H activation process and instead reveal a degradation of the S,O-ligand (a loss of 86 Da). On contrary, the complexes with the larger and more flexible ligands dppp and dppb show the C–H activation reaction (Fig. 9c and S21(iii)†). Hence, the C–H activation reaction requires proximity of the phenyl ring and the S,O-ligand which points towards a tight transition structure for this process.

### DFT calculations

**Fig. 10:**
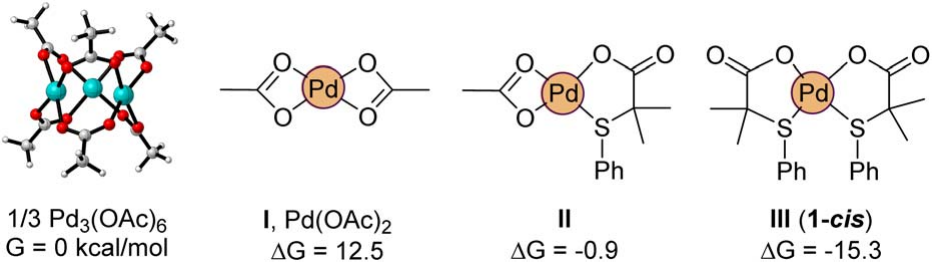
Computed relative stability of trimeric and monomeric palladium acetate with L2 containing complexes.

Density Functional Theory calculations [M06/def2tzvpp (IEF-PCM, acetic acid) with Gaussian software] were also carried out to get more insight into the species involved in the mechanism,^26^ and more specifically, about the role played by the S,O ligand L2 in the reactivity enhancement. We considered that the observed reactivity is the result of the sum of different factors. First, we evaluated the possibility that the ligand is helping the formation of catalytically relevant monomeric palladium species. Indeed, palladium acetate is known to exist as a trimer, Pd_3_(OAc)_6_, which persists even in solution.^27^ In our hands, the dissociation of the trimer into the monomeric acetate is greatly disfavored, uphill by 12.5 kcal mol^−1^ (per palladium unit, Fig. 10). To our delight, the exchange of an acetate by ligand L2 stabilizes the monomeric complex by 13.4 kcal mol^−1^ (II). Following this trend, a second equivalent of L2 completely reverts the trimer/monomer equilibrium, ensuring the efficient dissociation of Pd_3_(OAc)_6_ with formation of complex 1-*cis* (III), as found experimentally (Scheme 2).

**Fig. 11:**
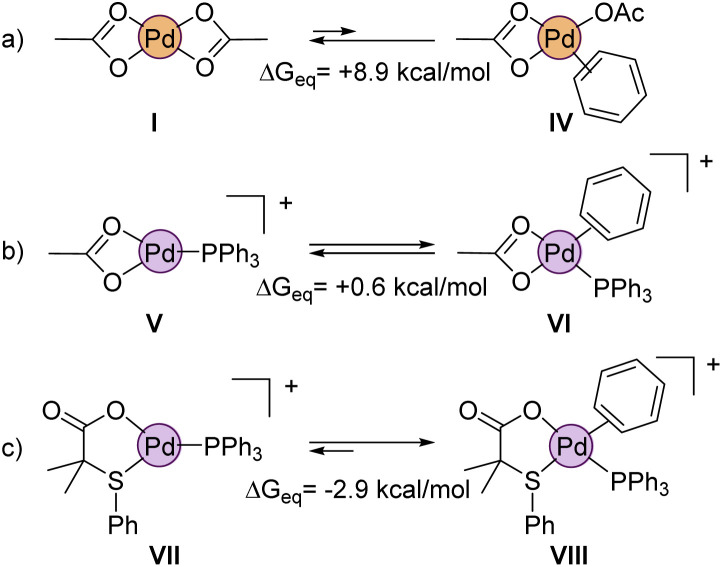
Relative affinity towards benzene coordination of neutral and cationic complexes.

We also wanted to prove theoretically the higher reactivity of the cationic complexes that are formed during the reaction, as suggested by the experimental results. In this regard, a crucial step in non-directed C–H activations is the coordination of the aromatic substrate to the palladium center, which is inherently difficult. Indeed, the calculations show that benzene hardly coordinates to neutral Pd(OAc)_2_ (IV, Δ*G*_eq_ = +8.9 kcal mol^−1^, Fig. 11a). Obviously, the situation would be quite different in a cationic complex like V (Fig. 11b), which is coordinatively unsaturated. But even in this equilibrium, the formation of the benzene coordinated complex VI is just isoenergetic with V. Noteworthy, complex VII, which contains our ligand L2 (instead of acetate) shows a much larger affinity towards the benzene coordination, forming VIII, which is *ca.* 3 kcal mol^−1^ lower in energy than VII.

**Fig. 12:**
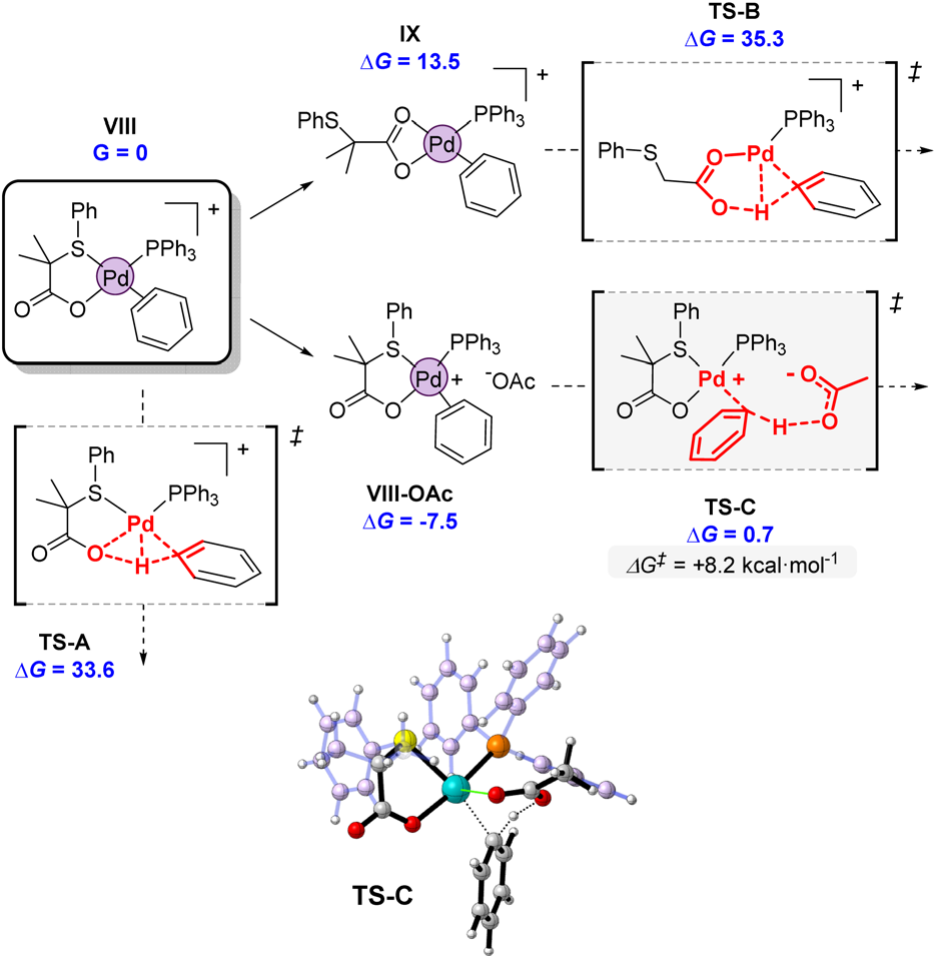
Computed transition states for the C–H activation promoted by the internal S,O ligand (TS-A and TS-B) or an external acetate (TS-C). Energies are in kcal mol^−1^ referred to VIII as *G* = 0.

Considering the intermediacy of complex VIII as an active species in the potential energy surface, we next evaluated different alternatives for a plausible C–H bond cleavage (Fig. 12).^28^ In line to what has been proposed in other catalytic systems, we computed the involvement of the internal S,O-ligand in 4- or 6-membered transition states, and an external acetate anion present in the reaction medium.^29^ As expected, the participation of the O atom of the S,O-ligand in a 4-membered transition state (TS-A) was computed to demand a high activation energy (Δ*G*^‡^ = 33.6 kcal mol^−1^), due to the strained disposition of the Pd–O–H–C atoms in the structure. This type of TS has been seldom proposed in some CMD mechanisms but it has never been confirmed computationally.^30^ A much more favored situation was expected in TS-B, which is a conventional 6-membered CMD transition state.^31^ However, in this mechanism, the coordination pattern of the S,O-ligand must completely switch from VIII to IX, *via* decoordination of the S atom and formation of an acetate-type coordination to palladium. This change in coordination involves an increase of 13.5 kcal mol^−1^ in the energy of IX. Furthermore, the cationic character of the palladium center weakens the C–H bonds of benzene, but at the same time it decreases the basicity of the acetate fragment, rendering a final activation energy of 35.3 kcal mol^−1^ for TS-B. This value is again too high to be feasible at the experimental conditions. Furthermore, TS-B would make difficult to rationalize the distinguishing reactivity of our S,O-ligand compared to a standard acetate, since the S atom does not directly participate in the coordination during the transition state. We note, that both TS-A and TS-B activation energies agree well with large C–H activation energies observed in the gas-phase unimolecular reactions (*vide supra*).

**Scheme 5:**
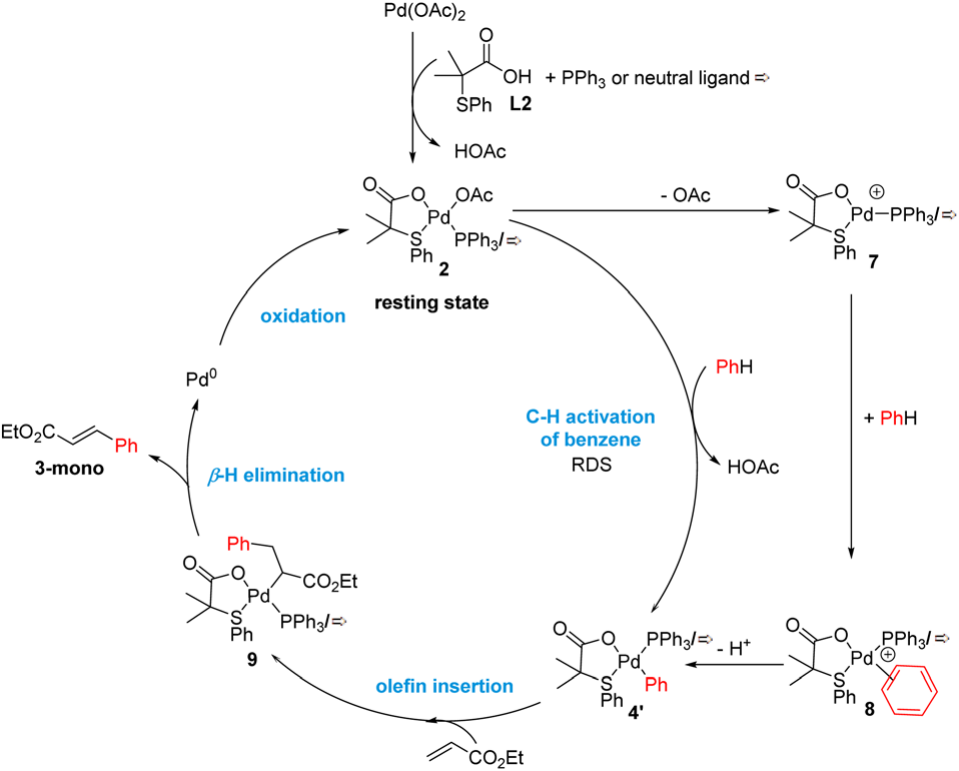
Proposed catalytic cycle.

Alternatively, bimolecular mechanism through the activation of the C–H bond by an external acetate was found to be strikingly favored, as indicated by the very low activation energy of the transition state TS-C. The incoming acetate unit can initially form an ion pair as in VIII-OAc, stabilizing the complex in 7.5 kcal mol^−1^. From there, the activation energy of the C–H bond cleavage amounts to only 8.2 kcal mol^−1^, one of the lowest values computed for a C–H bond cleavage. The participation of an external base has been hypothesized and computed by different authors,^32^ but it is not in general the preferred option. In neutral systems, it presents the drawback that the deprotonation renders an anionic palladium complex. Therefore, we believe that in our case, the cationic palladium system VIII is perfectly suited for the participation of an external base, since after deprotonation, a more stable neutral complex like 4 would be formed (Scheme 5). Furthermore, the reactive cationic palladium promotes the cleavage of aromatic C–H bonds by increasing their acidity, while the incoming acetate maintains its full basic strength.

### Proposed catalytic cycle in the presence of S,O-ligand

By combining all the experimental data, we propose the mechanism outlined in Scheme 5 for the C–H olefination of arenes in the presence of the S,O-ligand. We draw the intermediates with PPh_3_ as ligand since we isolated and identified many of these complexes in the catalytic reaction and similar mechanism is expected to occur with and without PPh_3_. Nevertheless, in the case of performing the reaction without PPh_3_, complexes bearing an innocent ligand or polymeric species might be formed. First, Pd(OAc)_2_ reacts with ligand L2 and PPh_3_ forming complex 2, which we isolated and demonstrated that is catalytically active. We observed by NMR that complex 2 is the catalyst resting state, indicating that C–H activation is the RDS. This is in agreement with the order of the reaction and the large KIE values observed. After the C–H activation step, complex 4′ is formed. We propose that the C–H activation takes place *via* cationic monomeric palladium species 7, which are formed from complex 2 after losing an AcO^−^ ligand. The formation of cationic species is supported by the inverse order in AcO^−^. Additionally, a monomeric cationic [(PPh_3_)_2_Pd(L2)]^+^ complex, which is related with the structure of palladium species 7, was detected by ESI-MS and its reactivity in intramolecular C–H activation processes proved. DFT calculations corroborate the higher reactivity of cationic complexes in the C–H activation of benzene. After the C–H activation step, complex 4 reacts with ethyl acrylate providing complex 9 which then undergoes β-H elimination to provide the olefinated compound and Pd(0) species. The oxidation of these species by an oxidant completes the catalytic cycle. From the comparison of the experiments performed with and without ligand, we propose that the S,O-ligand triggers the formation of cationic palladium species that are more reactive than the neutral species in the C–H activation step, which is in both cases the RDS.

## Conclusions

This article describes a detailed mechanistic study on the role of the S,O-ligand in the Pd-catalyzed C–H olefination reactions of non-directed arenes. Several monomeric Pd/S,O-ligand complexes before and after the C–H activation step have been isolated and fully characterized and their reactivities have been evaluated. NMR and kinetics studies, and KIE values indicate that the C–H activation step is RDS in both cases with and without S,O-ligand. Moreover, these studies suggest that the S,O-ligand triggers the formation of Pd cationic species. Additionally, a cationic Pd-complex has been detected by ESI-MS and its reactivity in C–H activation processes studied. DFT calculations corroborate the higher reactivity of cationic complexes in C–H activation processes.

Together, these studies shed light on the features of the Pd/S,O-ligand catalyst that make it so effective in promoting Pd-catalyzed C–H functionalization reactions. We expect that the insights presented herein will provide the foundations for the rational design of novel and more active catalyst for C–H activation processes.

## Data availability

The ESI† is available and contains experimental procedures, compounds characterization and computational studies.

## Author contributions

K. N. performed all the reactions, NMR and kinetic experiments. E. G. B. performed the DFT calculations. E. O. resolved the crystal structures. J. M and J. R. performed the MS experiments. M. A. F.-I. supervised the project. K. N and M. A. F.-I. wrote the manuscript with input from all the authors.

## Conflicts of interest

There are no conflicts to declare.

## Supplementary Material

SC-014-D2SC06840K-s001

SC-014-D2SC06840K-s002
