# Supplementary material for: Mechanistic studies of the palladium-catalyzed S,O-ligand promoted C–H olefination of aromatic compounds
Source: Chem Sci. 2023 Feb 16;14(11):2943–53. doi: 10.1039/d2sc06840k (PMC10016329; doi:10.1039/d2sc06840k)
Supplement: SC-014-D2SC06840K-s001 [file SC-014-D2SC06840K-s001.pdf]

Electronic Supplementary Material (ESI) for Chemical Science.
This journal is © The Royal Society of Chemistry 2023

Supporting information for

Mechanistic Studies of Palladium-Catalyzed S,O-Ligand promoted C–H Olefination of Aromatic compounds

Kananat Naksomboon,[1] Enrique Gómez-Bengoa,[2] Jaya Mehara,[3] Jana Roithová,[3] Edwin Otten,[4] and M. Ángeles Fernández-Ibáñez*[1]

[1] Van't Hoff Institute for Molecular Sciences, University of Amsterdam, Science Park 904, 1098 XH Amsterdam, The Netherlands.

[2] Department of Organic Chemistry I, Universidad País Vasco, UPV/EHU, Apdo. 1072, 20080, San Sebastian, Spain.

[3] Institute for Molecules and Materials, Radboud University, Heyendaalseweg 135, 6525 AJ Nijmegen, The Netherlands.

[4] Stratingh Institute for Chemistry, University of Groningen, Nijenborgh 4, 9747 AG Groningen, The Netherlands.

*Corresponding author, e-mail: M.A.FernandezIbanez@uva.nl

Table of contents

1. General information S3
2. Identification of active palladium complexes S3
3. Identification of palladium complexes during the catalytic reaction S15
4. Kinetic investigations S15
5.NMR Spectra S30
6. Mass spectrometric measurements S37
7. DFT calculations S42
8. References S53

1. General information

Chromatography: Silicycle Silica Flash P60 size 40–63 µm (230–400 mesh), TLC: Merck silica gel 60 (0.25mm). Visualization of the TLC was performed by UV, phosphomolybdic acid and $KMnO_4$ staining. Mass spectra were recorded on a AccuTOF GC v 4g, JMS-T100GCV mass spectrometers. ^{1}H, ^{13}C and ^{31}P NMR were recorded on a Bruker DRX 500, Bruker AMX 400 and Bruker DRX 300. Chemical shift values are reported in ppm with the solvent resonance as the internal standard ($CDCl_3$: δ 7.26 for ^{1}H, δ 77.16 for ^{13}C, C_6D_6: δ 7.16 for ^{1}H, δ 128.06 for ^{13}C, THF-d_8: δ 3.58 for ^{1}H, δ 67.57 for ^{13}C; AcOD-d_4: δ 2.04 for ^{1}H). Data are reported as follows: chemical shifts, multiplicity (s = singlet, d = doublet, dd = doublet of doublets, t = triplet, q = quartet, bs = broad singlet, m = multiplet), coupling constants (Hz) and integration. Infrared spectra were recorded on a Bruker IFS 28 FT-spectrophotometer and wavelengths are reported in cm^{-1}. GC analysis was performed on a Shimadzu GC-2010 Plus Gas Chromatograph using SH-Rxi-5HT (30 m, 0.25 mm, D 0.25 µm) column. All reagents and solvents that were not mentioned were used as received. $Pd(OAc)_2$ was purchased from Strem. 3-Methyl-2-(phenylthio)butanoic acid (**L1**) and 2-methyl-2-(phenylthio)propanoic acid (**L2**) were prepared according to the literature.[1]

2. Identification of active palladium complexes

<u>Procedure for the synthesis of complexes 1-*cis* and 1-*trans*</u>

A solution of $Pd(OAc)_2$ (22.5 mg, 0.1 mmol, 1 equiv) and ligand **L2** (39.3 mg, 0.2 mmol, 2 equiv) in CH_2Cl_2 (3 mL) was stirred at room temperature overnight. The reaction was filtrated through a pad of Celite and evaporated to dryness to afford palladium complexes **1-*cis*** and **1-*trans*** as an organge solid (50.3 mg, quantitative yield). Single crystal suitable for X-ray crystallography was obtained by crystallization with a two-solvent system ($CHCl_3$/*n*-heptane).

Complexes 1-*cis* and 1-*trans*

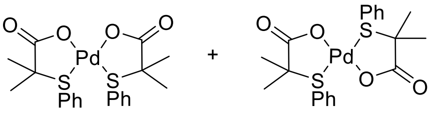

1-*cis* **1-*trans***

^{1}H NMR (500 MHz, $CDCl_3$) δ = 8.01 – 7.87 (m, $4H_{cis+trans}$), 7.65 – 7.16 (m, $16H_{cis+trans}$), 1.92 (s, $6H_{cis}$), 1.75 (s, $6H_{trans}$), 1.22 (s, $6H_{cis}$), 1.20 (s, $6H_{trans}$); IR ν = 1649, 1287, 1177, 746, 686, 494 cm^{-1}; HRMS (FD) calcd for $C_{20}H_{22}O_4PdS_2$ $[M]^+$: 495.9994; found: 495.9972.

Crystal structure information of complex **1-*cis***

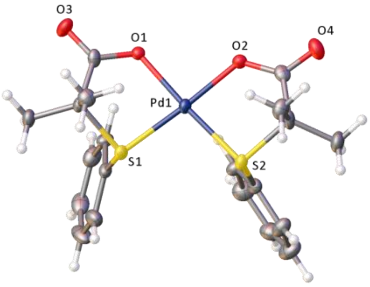

CCDC Number	1567101
Empirical formula	$C_{20}H_{22}O_4S_2Pd$
Formula weight	496.94
Temperature/K	N/A
Crystal system	triclinic
Space group	P-1

a/Å	9.0235(3)
b/Å	10.1775(4)
c/Å	11.8976(5)
α/°	81.443(2)
β/°	73.982(2)
γ/°	76.298(2)
Volume/Å^3	1016.25(7)
Z	2
ρ_{calc}g/cm^3	1.6239
μ/mm^{-1}	1.141
F(000)	502.7
Crystal size/mm^3	0.479 x 0.178 x 0.114
Radiation	MoKα (λ = 0.71073)
2Θ range for data collection/°	5.22 to 50.08
Index ranges	-10 ≤ h ≤ 10, -12 ≤ k ≤ 12, -14 ≤ l ≤ 14
Reflections collected	15109
Independent reflections	3577 [R_{int} = 0.0346, R_{sigma} = 0.0279]
Data/ restraints/ parameters	3577/0/248
Goodness-of-fit on F^2	0.913
Final R indexes [I>2σ (I)]	R_1 = 0.0266, wR_2 = 0.0916
Final R indexes [all data]	R_1 = 0.0347, wR_2 = 0.1103
Largest diff. peak/hole / e Å^{-3}	0.62/-0.68

Table S1. Fractional atomic coordinates (×10^4) and equivalent isotropic displacement parameters (Å^2×10^3) for **1-*cis***. U_{eq} is defined as 1/3 of of the trace of the orthogonalised U_{IJ} tensor.

**Atom**	***x***	***y***	***z***	**U(eq)**
Pd1	2968.6(3)	5559.8(2)	3054.7(2)	17.75(13)
S1	4291.4(10)	3988.7(8)	4169.7(7)	18.6(2)
S2	4356.2(10)	7195.2(8)	2872.8(7)	18.7(2)
O1	1637(3)	4182(2)	3167(2)	17.1(5)
O2	1734(3)	6870(2)	2021(2)	19.5(5)
O3	916(3)	2283(3)	4021(2)	32.3(6)
O4	1186(3)	8939(3)	1183(2)	33.1(6)
C1	1681(4)	3143(3)	3934(3)	20.7(7)
C2	2698(4)	3032(3)	4809(3)	19.5(7)
C15	5905(4)	6954(3)	1552(3)	19.9(7)
C12	2791(4)	8625(3)	2526(3)	24.1(8)
C3	3388(4)	1558(3)	5161(3)	25.1(8)
C4	1698(4)	3787(4)	5868(3)	26.9(8)
C5	5722(4)	2955(3)	3131(3)	20.5(7)
C6	5393(4)	2532(4)	2194(3)	24.7(8)
C11	1850(4)	8129(4)	1830(3)	21.8(7)
C16	6007(4)	6009(4)	793(3)	27.3(8)
C17	7315(5)	5768(4)	-138(4)	35.3(9)

C10	7248(4)	2603(4)	3294(3)	31.4(9)
C14	3489(5)	9825(4)	1879(4)	32.2(9)
C7	6579(4)	1726(4)	1428(3)	30.5(9)
C20	7094(5)	7671(4)	1377(3)	30.6(9)
C19	8389(5)	7442(5)	423(4)	40.6(10)
C13	1696(4)	8957(4)	3739(3)	29.2(8)
C8	8088(4)	1358(4)	1589(4)	35.5(10)
C18	8499(5)	6491(5)	-340(4)	37.2(10)
C9	8426(5)	1805(5)	2508(4)	39.1(10)

Procedure for the synthesis of complex 2

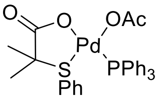

2

A solution of $Pd(OAc)_2$ (56.1 mg, 0.25 mmol, 1 equiv), ligand **L2** (49.0 mg, 0.25 mmol, 1 equiv) and PPh_3 (65.6 mg, 0.25 mmol, 1 equiv) in CH_2Cl_2 (8 mL) was stirred at room temperature for 1 h. The reaction was filtrated through a pad of Celite and evaporated to dryness to afford palladium complex **2** as an orange solid (131.5 mg, 84% yield). Single crystal suitable for X-ray crystallography was obtained by crystallization with a two-solvent system (THF/*n*-heptane). ^{1}H NMR (400 MHz, $CDCl_3$) δ = 7.59 (dd, *J* = 12.1, 7.7 Hz, 8H), 7.44 (t, *J* = 7.4 Hz, 4H), 7.33 – 7.27 (m, 8H), 2.02 (s, 3H), 1.75 (bs, 3H), 1.10 (s, 3H); ^{13}C NMR (101 MHz, $CDCl_3$) δ = 179.7, 179.7, 134.2, 134.1, 134.0, 131.7, 131.7, 131.6, 129.9, 128.7, 128.6, 127.0, 126.4, 126.4, 63.1, 27.7, 25.6, 21.7; ^{31}P NMR (162 MHz, $CDCl_3$) δ = 26.45 (s); IR ν = 3055, 2925, 1645, 1436, 1302, 1098, 691, 536 cm^{-1}; HRMS (FD) calcd for $C_{30}H_{29}O_4PPdS$ $[M]^+$: 622.0559; found: 622.0543.

Crystal structure information of complex **2**

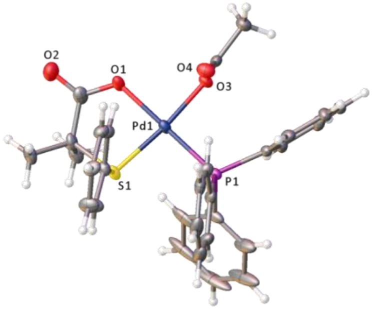

CCDC number	1969711
Empirical formula	$C_{30}H_{29}O_4PPdS$
Formula weight	622.96
Temperature/K	100(2)
Crystal system	monoclinic
Space group	$P2_1/c$
a/Å	10.1765(4)
b/Å	28.3513(11)
c/Å	9.7423(4)
α/°	90
β/°	106.9510(10)
γ/°	90
Volume/Å^3	2688.70(19)

Z	4
ρ_{calc}g/cm^3	1.539
μ/mm^{-1}	0.862
F(000)	1272.0
Crystal size/mm^3	0.23 x 0.17 x 0.05
Radiation	MoKα (λ = 0.71073)
2Θ range for data collection/°	5.85 to 52.912
Index ranges	-12 ≤ h ≤ 12, -34 ≤ k ≤ 35, -12 ≤ l ≤ 12
Reflections collected	35241
Independent reflections	5489 [R_{int} = 0.0620, R_{sigma} = 0.0381]
Data/ restraints/ parameters	5489/0/337
Goodness-of-fit on F^2	1.149
Final R indexes [I>2σ (I)]	R_1 = 0.0440, wR_2 = 0.0808
Final R indexes [all data]	R_1 = 0.0585, wR_2 = 0.0852
Largest diff. peak/hole / e Å^{-3}	0.92/-1.06

Table S2. Fractional atomic coordinates (×10^4) and equivalent isotropic displacement parameters (Å^2×10^3) for **2**. U_{eq} is defined as 1/3 of of the trace of the orthogonalised U_{IJ} tensor.

**Atom**	***x***	***y***	***z***	**U(eq)**
Pd1	6811.8(3)	6472.5(2)	6676.7(3)	17.49(8)
S1	5116.3(9)	6820.7(3)	4935.4(10)	20.5(2)
P1	8060.0(10)	6296.9(3)	5194.7(10)	17.7(2)
O1	5622(3)	6614.2(10)	8006(3)	25.8(6)
O2	3730(3)	6951.1(11)	8232(3)	36.6(7)
O3	8240(3)	6190.6(9)	8402(3)	20.3(5)
O4	7013(3)	5531.6(10)	7732(3)	27.3(6)
C1	4576(4)	6888.1(14)	7570(4)	27.2(9)
C2	4411(4)	7168.2(14)	6177(4)	24.4(9)
C3	5349(4)	7607.4(15)	6502(5)	30.8(10)
C30	8892(4)	5533.7(15)	9900(4)	29.2(9)
C29	7943(4)	5754.4(14)	8565(4)	20.0(8)
C4	2925(4)	7300.9(15)	5440(5)	31.3(10)
C5	3878(4)	6370.2(13)	4242(4)	19.9(8)
C6	3603(4)	6012.9(13)	5088(4)	20.8(8)
C7	2664(4)	5667.3(14)	4460(4)	24.4(9)
C8	2016(4)	5674.3(15)	2990(4)	26.0(9)
C9	2300(4)	6029.1(16)	2145(4)	32.4(10)
C10	3225(4)	6380.2(15)	2775(4)	27.8(9)
C11	7000(4)	5950.2(14)	3708(4)	20.9(8)
C12	6300(4)	5564.4(14)	4053(4)	21.1(8)
C13	5397(4)	5304.9(15)	2966(5)	27.4(9)
C14	5172(4)	5433.5(17)	1551(5)	35.1(11)
C15	5840(4)	5817.3(19)	1198(4)	38.4(12)
C17	9607(4)	5966.2(13)	6097(4)	18.6(8)
C16	6761(4)	6079.5(16)	2277(4)	28.4(9)

C18	9627(4)	5478.9(14)	6128(4)	22.6(8)
C19	10784(4)	5244.9(15)	6967(4)	24.3(9)
C20	11910(4)	5493.3(15)	7787(4)	25.9(9)
C21	11906(4)	5978.2(15)	7754(4)	26.3(9)
C22	10751(4)	6216.1(14)	6902(4)	23.7(8)
C23	8683(4)	6797.3(16)	4414(5)	32.4(11)
C24	8351(5)	7248.5(16)	4722(5)	42.2(13)
C25	8856(6)	7636(2)	4147(7)	69(2)
C26	9659(7)	7566(3)	3261(8)	94(3)
C27	9986(5)	7120(3)	2945(7)	82(3)
C28	9520(4)	6726(2)	3531(5)	48.0(14)

Reactivity of Pd complexes in the C–H olefination of benzene

Pd catalyst (12.5 µmol, 5 mol%), *tert*-butyl peroxybenzoate (47 µL, 0.25 mmol, 1 equiv), ethyl acrylate (27 µL, 0.25 mmol, 1 equiv), benzene (0.25 mL, 2.8 mmol, 11.2 equiv) and AcOH (1.25 mL, 0.2 M) were added into a pressure tube. The pressure tube was sealed with a screw cap and the reaction was placed in a 100 °C pre-heated oil bath and stirred for 2 h. The resulting mixture was diluted with EtOAc, filtered through a plug of Celite and concentrated under reduced pressure. The ^{1}H NMR yield was determined by adding CH_2Br_2 (17.5 µL, 0.25 mmol, 1 equiv) as an internal standard.

Ethyl cinnamate (3-mono)

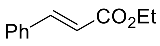

3-mono

^{1}H NMR spectra data matched with the spectra data reported in the literature for this compound.[2] ^{1}H NMR (300 MHz) δ = 7.69 (d, *J* = 16.0 Hz, 1H), 7.54 - 7.51 (m, 2H), 7.40 - 7.36 (m, 3H), 6.44 (d, *J* = 16.0 Hz, 1H), 4.27 (q, *J* = 7.1 Hz, 2H), 1.34 (t, *J* = 7.1 Hz, 3H).

Ethyl 3,3-diphenylacrylate (3-di)

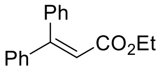

3-di

^{1}H NMR spectra data matched with the spectra data reported in the literature for this compound.[3] ^{1}H NMR (400 MHz) δ = 7.42 – 7.33 (m, 8H), 7.26 – 7.23 (m, 2H), 6.40 (s, 1H), 4.07 (q, *J* = 7.2 Hz, 2H), 1.14 (t, *J* = 7.1 Hz, 3H).

Table S3. The amount of catalyst.

Entry	Amount of catalyst
1	$Pd(OAc)_2$ 2.8 mg
2	$Pd(OAc)_2$ 2.8 mg and **L2** 2.5 mg
3	$Pd(OAc)_2$ 2.8 mg and **L2** 4.9 mg (25 µmol, 10 mol%)
4	Complexes **1-*cis*** and **1-*trans*** 6.2 mg
5	$Pd(OAc)_2$ 2.8 mg, **L2** 2.5 mg and PPh_3 3.3 mg
6	Complex **2** 7.8 mg

Kinetic profile of the C–H olefination of benzene with different Pd-catalyst

Pd catalyst (5 mol%), *t*-butyl peroxybenzoate (1 equiv), ethyl acrylate (1 equiv), benzene (11.2 equiv) and AcOH (0.2 M) were added into a pressure tube. The pressure tube was sealed with a crimp cap with septa and the reaction was placed in a 100 °C pre-heated oil bath. The reaction

was followed during the indicated time by sampling 50 µL. PhCl (10 µL) was added in each sample as an internal standard for quantitative GC analysis. The reaction mixture was diluted with EtOAc (1 mL). The organic layer was quenched with saturated aqueous $NaHCO_3$ solution (1 mL). The organic layer was filtered through a plug of anh. $MgSO_4$ and analyzed by GC.

$Pd(OAc)_2$ (5.6 mg, 25 µmol, 5 mol%), *t*-butyl peroxybenzoate (94 µL, 0.5 mmol, 1 equiv), ethyl acrylate (53 µL, 0.5 mmol, 1 equiv), benzene (0.5 mL, 5.6 mmol, 11.2 equiv) and AcOH (2.5 mL, 0.2 M) were added into a pressure tube. The pressure tube was sealed with a crimp cap with septa and the reaction was placed in a 100 °C pre-heated oil bath. The reaction was followed during the indicated time by sampling 50 µL. PhCl (10 µL) was added in each sample as an internal standard for quantitative GC analysis. The reaction mixture was diluted with EtOAc (1 mL). The organic layer was quenched with saturated aqueous $NaHCO_3$ solution (1 mL). The organic layer was filtered through a plug of anh. $MgSO_4$ and analyzed by GC.

Parallel reaction using $Pd(OAc)_2$ and ligand **L2** was performed to compare the kinetic profile. $Pd(OAc)_2$ (5.6 mg, 25 µmol, 5 mol%), ligand **L2** (4.9 mg, 25 µmol, 5 mol%), *t*-butyl peroxybenzoate (94 µL, 0.5 mmol, 1 equiv), ethyl acrylate (53 µL, 0.5 mmol, 1 equiv), benzene (0.5 mL, 5.6 mmol, 11.2 equiv) and AcOH (2.5 mL, 0.2 M) were added into a pressure tube. The pressure tube was sealed with a crimp cap with septa and the reaction was placed in a 100 °C pre-heated oil bath. The reaction was followed during the indicated time by sampling 50 µL. PhCl (10 µL) was added in each sample as an internal standard for quantitative GC analysis. The reaction mixture was diluted with EtOAc (1 mL). The organic layer was quenched with saturated aqueous $NaHCO_3$ solution (1 mL). The organic layer was filtered through a plug of anh. $MgSO_4$ and analyzed by GC.

Parallel reaction using complexes **1-*cis*** and **1-*trans*** was performed to compare the kinetic profile. Complexes **1-*cis*** and **1-*trans*** (12.4 mg, 25 µmol, 5 mol%), *t*-butyl peroxybenzoate (94 µL, 0.5 mmol, 1 equiv), ethyl acrylate (53 µL, 0.5 mmol, 1 equiv), benzene (0.5 mL, 5.6 mmol, 11.2 equiv) and AcOH (2.5 mL, 0.2 M) were added into a pressure tube. The pressure tube was sealed with a crimp cap with septa and the reaction was placed in a 100 °C pre-heated oil bath. The reaction was followed during the indicated time by sampling 50 µL. PhCl (10 µL) was added in each sample as an internal standard for quantitative GC analysis. The reaction mixture was diluted with EtOAc (1 mL). The organic layer was quenched with saturated aqueous $NaHCO_3$ solution (1 mL). The organic layer was filtered through a plug of anh. $MgSO_4$ and analyzed by GC.

Parallel reaction using complex **2** was performed to compare the kinetic profile. Complex **2** (15.6 mg, 25 µmol, 5 mol%), *t*-butyl peroxybenzoate (94 µL, 0.5 mmol, 1 equiv), ethyl acrylate (53 µL, 0.5 mmol, 1 equiv), benzene (0.5 mL, 5.6 mmol, 11.2 equiv) and AcOH (2.5 mL, 0.2 M) were added into a pressure tube. The pressure tube was sealed with a crimp cap with septa and the reaction was placed in a 100 °C pre-heated oil bath. The reaction was followed during the indicated time by sampling 50 µL. PhCl (10 µL) was added in each sample as an internal standard for quantitative GC analysis. The reaction mixture was diluted with EtOAc (1 mL). The organic layer was quenched with saturated aqueous $NaHCO_3$ solution (1 mL). The organic layer was filtered through a plug of anh. $MgSO_4$ and analyzed by GC.

Table S4. Kinetic profile of the Pd-catalyzed C–H olefination of benzene.

Entry	Reaction time	GC yield			
		$Pd(OAc)_2$	$Pd(OAc)_2$ + ligand L2	Complexes 1-*cis* and 1-*trans*	Complex 2
1	15 min	1%	17%	9%	22%
2	30 min	3%	40%	21%	42%
3	45 min	4%	57%	31%	57%
4	1 h	6%	74%	40%	74%
5	1.5 h	10%	84%	52%	90%
6	2 h	14%	80%	62%	83%

Note: The reactions were performed at the same time to compare the kinetic profiles.

Procedure for the reaction of complex 2 and benzene

Complex **2** (30 mg, 48.0 µmol, 1 equiv) and benzene (0.25 mL) were added into a pressure tube. The pressure tube was sealed with a screw cap and the reaction was placed in a 100 °C pre-heated oil bath and stirred for 10 minutes. The reaction was cooled down to room temperature. The resulting mixture was diluted with CH_2Cl_2, filtered through a plug of Celite and concentrated under reduced pressure. The resulting crude was measured by ^1H NMR.

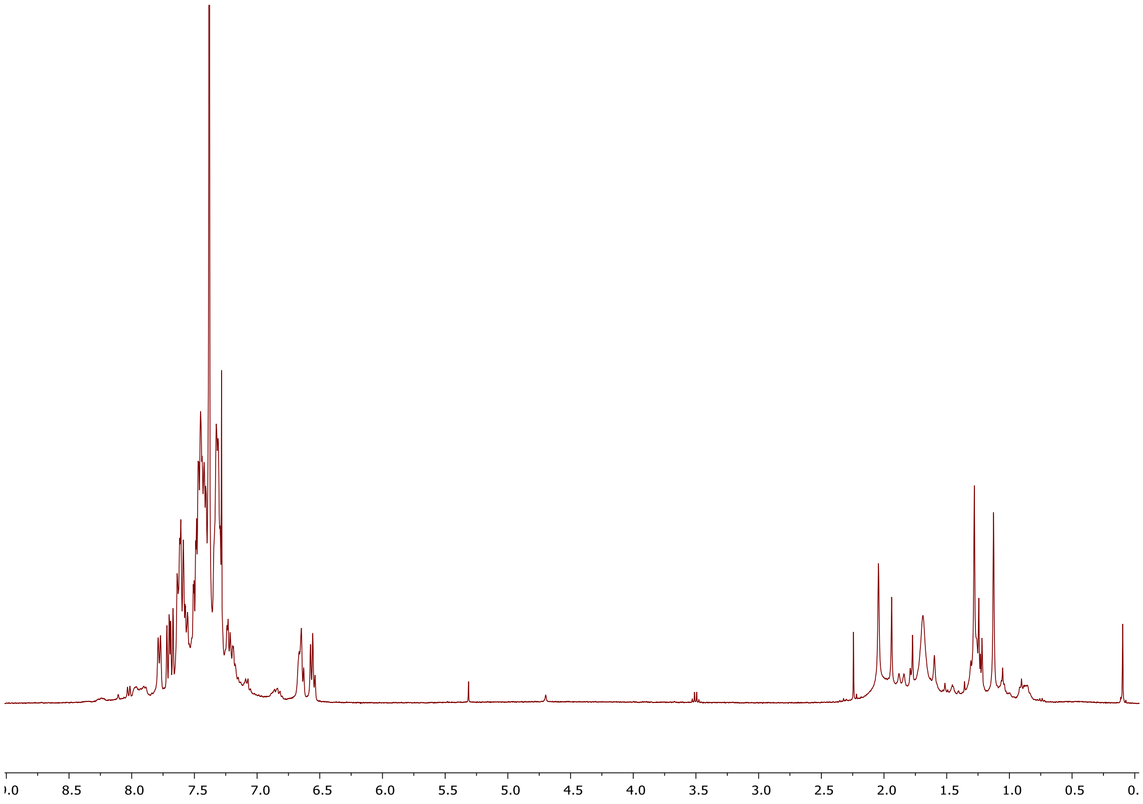

Procedure for the synthesis of complex 4’

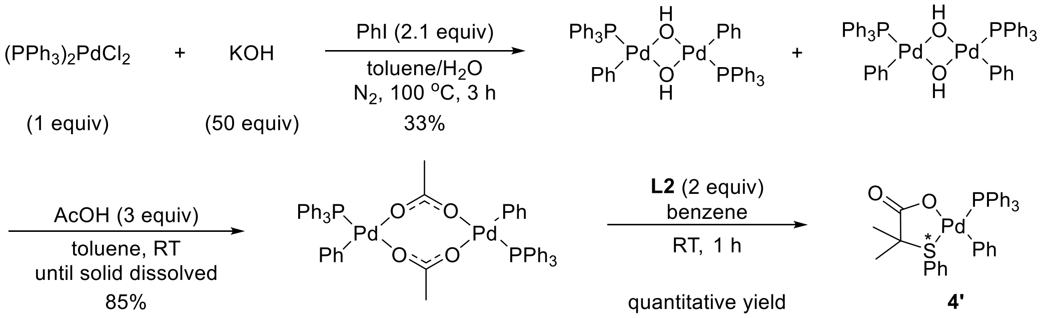

Complex [PhPd(PPh₃)(μ-OH)]₂

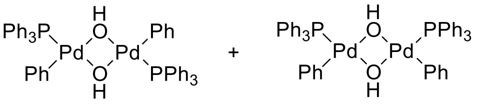

Complex [PhPd(PPh$_3$)(μ-OH)]$_2$ was prepared following the procedure described in the literature.[4] Degassed toluene (20 mL) and degassed water (4 mL) were added to Pd(PPh$_3$)$_2$Cl$_2$ (1.00 g, 1.42 mmol, 1 equiv) and KOH (4.00g, 71 mmol, 50 equiv) under nitrogen atmosphere followed by PhI (0.34 mL, 3.00 mmol, 2.1 equiv). The reaction was heated at 100 °C for 3 h with vigorous stirred. The hot solution was filtered through a short pad of Celite. The filtrated was separated and extracted with toluene (3 x 20 mL). The combined organic layers were dried over anh. MgSO$_4$, filtered and concentrated under reduced pressure to obtain a yellow solid. The resulting solid was triturated with acetone (5 mL) overnight and the obtained solid was filtered and washed with acetone (2 x 3 mL) to obtain a white solid (0.44 g, 33% yield). ^{1}H and ^{31}P NMR spectra data matched with the spectra data reported in the literature for this compound.[4a] ^{1}H NMR (400 MHz, CDCl$_3$) δ = 7.50 – 7.46 (m, 10H), 7.41 – 7.31 (m, 8H), 7.26 – 7.19 (m, 12H), 7.06 – 7.04 (m, 4H), 6.68 – 6.60 (m, 6H); ^{31}P NMR (162 MHz, CDCl$_3$) δ = 33.54 (s, *trans*), 32.85 (s, *cis*).

Complex [PhPd(PPh₃)(μ-OAc)]₂

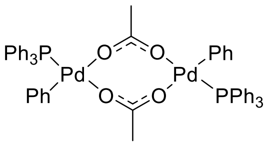

Complex [PhPd(PPh$_3$)(μ-OAc)]$_2$ was prepared following the procedure described in the literature.[4a] AcOH (60 μL, 1.05 mmol, 3 equiv) was added to the suspension of complex [PhPd(PPh$_3$)(μ-OH)]$_2$ (324 mg, 0.35 mmol, 1 equiv) in toluene (10 mL). The reaction was stirred at room temperature until solid was completely dissolved. The reaction was then concentrated under reduced pressure. The solid was washed with petroleum ether (3 x 5 mL) and dried under vacuum to obtain a yellow powder (300 mg, 85% yield). ^{1}H and ^{31}P NMR spectra data matched with the spectra data reported in the literature for this compound.[4a] ^{1}H NMR (400 MHz, C$_6$D$_6$) δ = 7.77 (bs, 4H), 7.52 – 7.47 (m, 12H), 6.93 – 6.83 (m, 24H), 1.59 (s, 6H); ^{31}P NMR (162 MHz, C$_6$D$_6$) δ = 29.97 (s).

Complex 4’

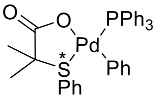

4'

Complex **4’** was prepared following a similar procedure to the one described in the literature.[4a] Complex [PhPd(PPh$_3$)(μ-OAc)]$_2$ (100 mg, 0.1 mmol, 1 equiv) and ligand **L2** (39.3 mg, 0.2 mmol, 2 equiv) were stirred in benzene at room temperature. After 1 h, the solvent was removed under reduced pressure to obtain a pale yellow solid (130 mg, quantitative yield). Single crystal suitable for X-ray crystallography was obtained by crystallization with a two-solvent system (THF/*n*-heptane). ^{1}H NMR (400 MHz, THF-d$_8$) δ = 7.78 (d, *J* = 6.8 Hz, 2H), 7.52 – 7.37 (m, 12H), 7.30 – 7.28 (m, 6H),

6.64 (d, J = 6.6 Hz, 2H), 6.53 (t, J = 6.8 Hz, 1H), 6.45 (t, J = 7.2 Hz, 2H), 1.54 (s, 6H); ^{13}C NMR (101 MHz, THF-d_8) δ = 179.1, 146.8 (d, J = 7.2 Hz), 136.7 (d, J = 3.8 Hz), 135.5, 135.4, 135.2, 131.7, 131.5 (d, J = 1.9 Hz), 131.2, 131.2, 130.5, 130.4, 129.4, 129.3, 129.2, 128.1, 123.5, 59.5, 27.7; ^{31}P NMR (162 MHz, THF-d_8) δ = 24.48 (s); IR ν = 1620, 1303, 1097, 735, 690, 529 cm^{-1}; HRMS (FD) calcd for $C_{34}H_{31}O_2PPdS$ [M]$^+$: 640.0817; found: 640.1009.

Crystal structure information of complex **4'A**

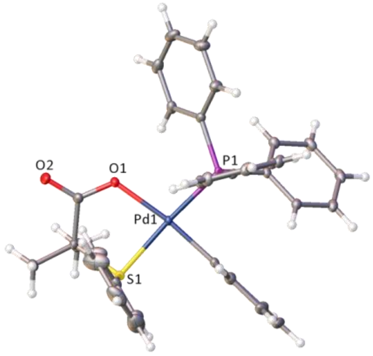

CCDC number	1969712
Empirical formula	$C_{34}H_{31}O_2PPdS$
Formula weight	641.02
Temperature/K	100(2)
Crystal system	orthorhombic
Space group	$P2_12_12_1$
a/Å	9.7129(4)
b/Å	11.8850(4)
c/Å	24.9669(9)
α/°	90
β/°	90
γ/°	90
Volume/Å^3	2882.12(19)
Z	4
ρ_{calc}g/cm^3	1.477
μ/mm^{-1}	0.802
F(000)	1312.0
Crystal size/mm^3	0.330 x 0.300 x 0.120
Radiation	MoKα (λ = 0.71073)
2Θ range for data collection/°	5.976 to 59.268
Index ranges	-13 ≤ h ≤ 13, -16 ≤ k ≤ 16, -34 ≤ l ≤ 34
Reflections collected	53234
Independent reflections	8114 [R_{int} = 0.0236, R_{sigma} = 0.0172]
Data/ restraints/ parameters	8114/0/354
Goodness-of-fit on F^2	1.086
Final R indexes [I>2σ (I)]	R_1 = 0.0163, wR_2 = 0.0389
Final R indexes [all data]	R_1 = 0.0173, wR_2 = 0.0392
Largest diff. peak/hole / e Å^{-3}	0.29/-0.51
Flack parameter	-0.013(4)

Table S5. Fractional atomic coordinates (×10[4]) and equivalent isotropic displacement parameters (Å^2×10^3) for **4'A**. U_{eq} is defined as 1/3 of of the trace of the orthogonalised U_{IJ} tensor.

**Atom**	***x***	***y***	***z***	**U(eq)**
Pd1	7138.3(2)	5911.4(2)	6231.0(2)	9.25(3)
S1	7842.2(5)	7332.0(3)	6825.9(2)	12.71(8)
P1	6104.5(5)	4611.4(4)	5696.5(2)	9.27(8)
O1	8217.0(14)	4842.8(11)	6758.2(5)	14.1(3)
O2	9287.0(18)	4660.2(12)	7540.1(6)	23.8(3)
C1	6177(2)	7070.0(15)	5790.7(7)	11.9(3)
C2	4805(2)	7374.3(17)	5875.9(8)	15.5(4)
C3	4196(2)	8214.7(17)	5564.9(9)	20.5(4)
C4	4943(2)	8746.9(17)	5163.3(9)	22.6(4)
C5	6318(2)	8461.6(17)	5083.0(8)	19.7(4)
C6	6930.7(19)	7637.3(15)	5399.7(7)	14.6(3)
C7	8857(2)	5238.0(16)	7166.1(7)	13.6(3)
C8	9178(2)	6520.9(16)	7179.9(8)	14.0(4)
C9	10474(2)	6726.3(17)	6839.7(9)	20.3(4)
C10	9409(3)	6977.5(18)	7742.9(8)	22.1(4)
C11	6327(2)	7311.7(17)	7230.8(8)	17.2(4)
C12	5997(3)	6439(2)	7575.5(11)	34.3(6)
C13	4743(3)	6467(2)	7853.1(13)	44.9(8)
C14	3829(3)	7345(2)	7778.5(11)	36.1(6)
C15	4160(3)	8190(3)	7433.0(11)	36.7(6)
C16	5414(2)	8192(2)	7161.0(10)	27.3(5)
C17	4301.9(19)	4480.3(15)	5887.2(7)	11.1(3)
C18	4010.4(19)	4552.0(17)	6436.5(7)	14.6(4)
C19	2696(2)	4324.6(16)	6624.9(7)	17.1(4)
C20	1650.2(17)	4042.4(17)	6268.0(8)	16.9(3)
C21	1926.8(17)	3975.3(16)	5723.4(7)	15.7(3)
C22	3254.8(17)	4189.2(16)	5532.0(7)	12.9(3)
C23	6158.1(18)	4803.3(15)	4972.0(7)	11.3(3)
C24	5378.9(19)	5653.2(15)	4727.1(7)	13.2(3)
C25	5459.8(19)	5819.3(17)	4175.8(7)	15.9(3)
C26	6316(2)	5154.7(17)	3860.1(7)	17.7(4)
C27	7099(2)	4316.2(15)	4101.8(7)	17.0(3)
C28	7021.4(18)	4139.3(16)	4653.6(7)	14.0(3)
C29	6717.5(19)	3176.0(15)	5789.0(7)	11.9(3)
C30	8105.6(19)	2986.7(16)	5900.5(7)	15.5(4)
C31	8587(2)	1894.0(18)	5974.6(8)	20.1(4)
C32	7691(2)	993.8(18)	5940.7(8)	22.3(4)
C33	6309(2)	1172.7(17)	5820.5(9)	22.1(4)
C34	5824(2)	2257.6(17)	5744.8(8)	17.8(4)

Crystal structure information of complex **4’B**

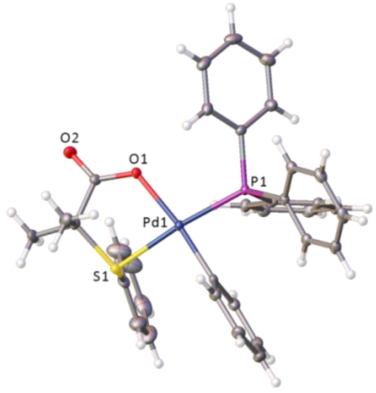

CCDC number	1969713
Empirical formula	$C_{34}H_{31}O_2PPdS$
Formula weight	641.02
Temperature/K	100(2)
Crystal system	orthorhombic
Space group	$P2_12_12_1$
a/Å	9.6976(5)
b/Å	11.8942(7)
c/Å	24.9749(15)
α/°	90
β/°	90
γ/°	90
Volume/Å^3	2880.7(3)
Z	4
ρ_{calc}g/cm^3	1.478
μ/mm^{-1}	0.802
F(000)	1312.0
Crystal size/mm^3	0.12 x 0.10 x 0.06
Radiation	MoKα (λ = 0.71073)
2Θ range for data collection/°	5.974 to 54.388
Index ranges	-12 ≤ h ≤ 12, -15 ≤ k ≤ 15, -32 ≤ l ≤ 32
Reflections collected	55231
Independent reflections	6397 [R_{int} = 0.0281, R_{sigma} = 0.0159]
Data/ restraints/ parameters	6397/0/354
Goodness-of-fit on F^2	1.085
Final R indexes [I>2σ (I)]	R_1 = 0.0155, wR_2 = 0.0380
Final R indexes [all data]	R_1 = 0.0164, wR_2 = 0.0385
Largest diff. peak/hole / e Å^{-3}	0.25/-0.43
Flack parameter	-0.030(4)

Table S6. Fractional atomic coordinates (×10^4) and equivalent isotropic displacement parameters (Å^2×10^3) for **4’B**. U_{eq} is defined as 1/3 of of the trace of the orthogonalised U_{IJ} tensor.

Atom	*x*	*y*	*z*	U(eq)
Pd1	7139.5(2)	5911.0(2)	3769.0(2)	9.73(4)

S1	7843.5(6)	7331.0(4)	3174.0(2)	13.23(10)
P1	6105.3(5)	4612.0(5)	4303.5(2)	9.65(10)
O1	8218.2(16)	4841.1(13)	3242.2(6)	14.3(3)
O2	9289(2)	4659.6(14)	2459.9(7)	24.1(4)
C1	6178(2)	7069.4(18)	4210.3(9)	12.4(4)
C2	4806(2)	7374(2)	4124.2(9)	15.9(4)
C3	4198(2)	8214(2)	4435.6(10)	21.2(5)
C4	4943(3)	8744(2)	4836.7(10)	22.8(5)
C5	6317(3)	8460(2)	4915.9(10)	20.4(5)
C6	6932(2)	7637.5(18)	4601.7(9)	14.9(4)
C7	8858(2)	5237.9(18)	2833.3(9)	13.6(4)
C8	9179(2)	6522.0(19)	2819.5(9)	14.8(4)
C9	10479(2)	6727(2)	3159.6(10)	21.0(5)
C10	9410(3)	6977(2)	2256.4(10)	22.5(5)
C11	6326(2)	7309(2)	2769.3(9)	17.7(5)
C12	5414(3)	8191(2)	2837.7(11)	28.2(6)
C13	4161(3)	8190(3)	2566.0(12)	36.7(7)
C14	3829(3)	7343(3)	2221.2(12)	36.0(7)
C15	4743(4)	6466(3)	2146.1(14)	45.4(9)
C16	5993(3)	6439(2)	2424.5(12)	34.7(7)
C17	6717(2)	3177.2(18)	4212.4(8)	12.5(4)
C18	5824(2)	2257(2)	4255.2(9)	17.7(5)
C19	6307(3)	1172(2)	4179.5(10)	22.4(5)
C20	7691(3)	992(2)	4060.1(9)	22.7(5)
C21	8586(3)	1894(2)	4026.0(9)	20.6(5)
C22	8105(2)	2985.2(19)	4098.7(9)	15.8(5)
C23	6158(2)	4803.6(18)	5028.3(8)	12.1(4)
C24	5380(2)	5655.6(18)	5273.0(9)	14.0(4)
C25	5461(2)	5819(2)	5823.6(9)	16.6(4)
C26	6317(2)	5155.2(19)	6139.7(9)	18.0(5)
C27	7100(3)	4316.4(18)	5897.5(8)	17.4(4)
C28	7021(2)	4138.7(19)	5347.2(8)	14.4(4)
C29	4299(2)	4479.4(18)	4112.7(9)	11.8(4)
C30	4012(2)	4552(2)	3564.6(9)	15.3(4)
C31	2699(2)	4324.7(19)	3375.6(9)	18.0(5)
C32	1650(2)	4042(2)	3731.2(9)	17.1(4)
C33	1929(2)	3975.8(19)	4276.4(9)	16.2(4)
C34	3253(2)	4190.1(19)	4467.7(8)	13.6(4)

Procedure for the reaction of complex 4' and ethyl acrylate

Complex **4'** (32 mg, 50.0 µmol, 1 equiv) and ethyl acrylate (0.25 mL, 2.35 mmol) were added into a pressure tube. The pressure tube was sealed with a screw cap and the reaction was placed in a 100 °C pre-heated oil bath and stirred for 2 h. The reaction was cooled down to room temperature. The resulting mixture was diluted with EtOAc, filtered through a plug of Celite and concentrated under reduced pressure. The ^{1}H NMR yield was determined by adding CH_2Br_2 (6.0

μL, 85.0 μmol, 1.7 equiv) as an internal standard. The reaction provided the olefinated product in 90% ^{1}H NMR yield.

3. Identification of palladium complexes during the catalytic reaction

Procedure for the C–H olefination of benzene (the reaction is performed in an NMR tube)

$Pd(OAc)_2$ (11.2 mg, 0.05 mmol, 50 mol%), ligand **L2** (9.8 mg, 0.05 mmol, 50 mol%), PPh_3 (13.1 mg, 0.05 mmol, 50 mol%), *tert*-butyl peroxybenzoate (19 μL, 0.1 mmol, 1 equiv), ethyl acrylate (11 μL, 0.1 mmol, 1 equiv), benzene (0.1 mL, 1.12 mmol, 11.2 equiv) and AcOD-d_4 (0.5 mL) were added into an NMR tube. The NMR tube was sealed with a screw cap. The reaction was placed in a 100 °C pre-heated oil bath and followed during the indicated time. ^{1}H and ^{31}P NMR spectra were collected at room temperature.

Procedure for the reaction of $Pd(OAc)_2$, ligand L2, PPh_3 and benzene (the reaction is performed in an NMR tube)

$Pd(OAc)_2$ (11.2 mg, 0.05 mmol, 1 equiv), ligand **L2** (9.8 mg, 0.05 mmol, 1 equiv), PPh_3 (13.1 mg, 0.05 mmol, 1 equiv), benzene (0.1 mL) and AcOD-d_4 (0.5 mL) were added into an NMR tube. The NMR tube was sealed with a screw cap. The reaction was placed in a 100 °C pre-heated oil bath and followed during the indicated time. ^{1}H and ^{31}P NMR spectra were collected at room temperature.

4. Kinetic investigations

Procedure for kinetic order of the reaction

$Pd(OAc)_2$, ligand **L1**, *tert*-butyl peroxybenzoate, ethyl acrylate, benzene, NaOAc and AcOH (1.25 mL) were added into a pressure tube. The pressure tube was sealed with a crimp cap with septa and the reaction was placed in a 100 °C pre-heated oil bath. The reaction was followed during the indicated time by sampling 50 μL. PhCl (10 μL) was added in each sample as an internal standard for quantitative GC analysis. The reaction mixture was diluted with EtOAc (0.5 mL). The organic layer was quenched with saturated aqueous $NaHCO_3$ solution (0.5 mL). The organic layer was filtered through a plug of anh. $MgSO_4$ and analyzed by GC.

Order of catalyst with ligand L1

General procedure was followed using $Pd(OAc)_2$ (2.5–12.5 mol%), ligand **L1** (0.0843 M in AcOH) (2.5–12.5 mol%), *tert*-butyl peroxybenzoate (47 μL, 0.25 mmol, 1 equiv), ethyl acrylate (27 μL, 0.25 mmol, 1 equiv), benzene (0.25 mL, 0.28 mmol, 11.2 equiv) and AcOH (1.25 mL, 0.2 M). The reaction was sampling at 4, 8, 12, 16 and 20 min.

Table S7. Amount of catalyst.

Entry	Amount of $Pd(OAc)_2$	Amount of ligand L1	AcOH (mL)	[catalyst] (mM)
1	1.4 mg, 6.25 μmol, 2.5 mol%	75 μL, 6.25 μmol, 2.5 mol%	1.175	4.17
2	2.8 mg, 12.50 μmol, 5 mol%	150 μL, 12.50 μmol, 5 mol%	1.1	8.33
3	4.2 mg, 18.75 μmol, 7.5 mol%	225 μL, 18.75 μmol, 7.5 mol%	1.025	12.50
4	5.6 mg, 25.00 μmol, 10 mol%	300 μL, 25.00 μmol, 10 mol%	0.95	16.67
5	7.0 mg, 31.25 μmol, 12.5 mol%	375 μL, 31.25 μmol, 12.5 mol%	0.875	20.83

Table S8. Data used to determine the rate of the reactions with different concentrations of catalyst with ligand **L1**.

Entry	[catalyst] (mM)	[product] (mM)				
		**0.07 h**	**0.13 h**	**0.2 h**	**0.26 h**	**0.33 h**
1	4.17	0.56	1.79	4.94	9.08	13.62
2	8.33	0.84	3.96	11.49	18.78	27.25
3	12.50	1.29	6.24	15.29	26.79	40.18
4	16.67	1.26	8.65	22.63	38.34	50.82
5	20.83	2.00	11.03	25.21	45.23	60.66

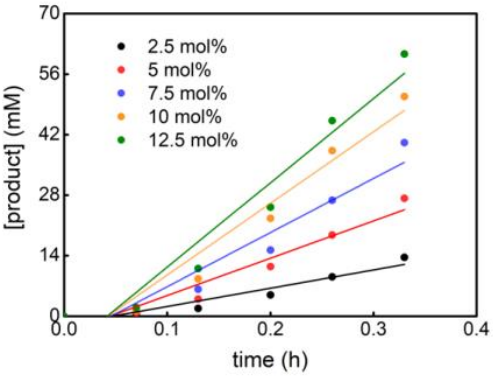

Figure S1. Plot of time (h) versus [product] (mM) with different concentrations of catalyst with ligand **L1** to determine the rate of the reactions.

Table S9. Data used to determine the order of catalyst with ligand **L1**.

Entry	[catalyst] (mM)	*k* (mM/h)	R^2	log[catalyst] (mM)	log*k* (mM/h)
1	4.17	42.26	0.9165	0.62	1.63
2	8.33	86.24	0.9349	0.92	1.94
3	12.50	125.01	0.9274	1.10	2.10
4	16.67	165.40	0.9438	1.22	2.22
5	20.83	194.96	0.9419	1.32	2.29

Order of benzene with ligand L1

General procedure was followed using $Pd(OAc)_2$ (2.8 mg, 12.5 µL, 5 mol%), ligand **L1** (0.0843 M in AcOH) (150 µL, 12.5 µmol, 5 mol%), *tert*-butyl peroxybenzoate (47 µL, 0.25 mmol, 1 equiv), ethyl acrylate (27 µL, 0.25 mmol, 1 equiv), benzene (6.7–24.7 equiv), C_6F_6 and AcOH (1.1 mL, 0.2 M). The reaction was sampling at 5, 10, 15, 20 and 25 min.

Table S10. Amount of benzene.

Entry	Amount of benzene	Amount of C_6F_6	[benzene] (mM)
1	0.15 mL, 1.675 mmol, 6.7 equiv	0.6 mL	837.50
2	0.25 mL, 2.8 mmol, 11.2 equiv	0.5 mL	1400.00
3	0.35 mL, 3.925 mmol, 15.7 equiv	0.4 mL	1962.50
4	0.45 mL, 5.05 mmol, 20.2 equiv	0.3 mL	2525.00
5	0.55 mL, 6.175 mmol, 24.7 equiv	0.2 mL	3087.50

Table S11. Data used to determine the rate of the reactions with different concentrations of benzene with ligand **L1**.

Entry	[benzene] (mM)	[product] (mM)				
		0.08 h	0.17 h	0.25 h	0.33 h	0.42 h
1	837.50	0	1.44	3.35	4.90	7.18
2	1400.00	1.01	2.10	5.43	9.39	13.47
3	1962.50	1.19	2.93	6.48	11.86	16.39
4	2525.00	0.84	4.15	10.78	19.72	27.10
5	3087.50	1.40	4.92	12.33	23.50	33.16

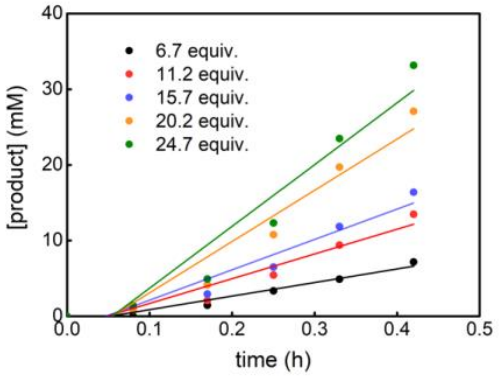

Figure S2. Plot of time (h) versus [product] (mM) with different concentrations of benzene with ligand **L1** to determine the rate of the reactions.

Table S12. Data used to determine the order of benzene with ligand **L1**.

Entry	[benzene] (mM)	*k* (mM/h)	R^2	log[benzene] (mM)	log*k* (mM/h)
1	837.50	17.93	0.9528	2.92	1.25
2	1400.00	32.69	0.9347	3.15	1.51
3	1962.50	40.07	0.9397	3.29	1.60
4	2525.00	67.80	0.9329	3.40	1.83
5	3087.50	81.71	0.9256	3.49	1.91

Order of oxidant with ligand L1

General procedure was followed using $Pd(OAc)_2$ (2.8 mg, 12.5 µL, 5 mol%), ligand **L1** (0.0843 M in AcOH) (150 µL, 12.5 µmol, 5 mol%), *tert*-butyl peroxybenzoate (0.5–1.25 equiv), ethyl acrylate (27 µL, 0.25 mmol, 1 equiv), benzene (0.25 mL, 2.8 mmol, 11.2 equiv) and AcOH (1.1 mL, 0.2 M). The reaction was sampling at 5, 10, 15, 20 and 25 min.

Table S13. Amount of oxidant.

Entry	Amount of oxidant	[oxidant] (mM)
1	24 µL, 0.125 mmol, 0.5 equiv	83.33
2	36 µL, 0.188 mmol, 0.75 equiv	125.00
3	47 µL, 0.25 mmol, 1 equiv	166.67
4	59 µL, 0.313 mmol, 1.25 equiv	208.33

Table S14. Data used to determine the rate of the reactions with different concentrations of oxidant with ligand **L1**.

Entry	[oxidant] (mM)	[product] (mM)				
		0.08 h	0.17 h	0.25 h	0.33 h	0.42 h
1	83.33	1.49	8.86	20.05	33.13	39.79
2	125.00	1.74	7.63	18.34	29.21	38.82
3	166.67	1.23	7.44	18.30	29.36	39.17
4	208.33	1.14	6.97	16.17	27.35	36.43

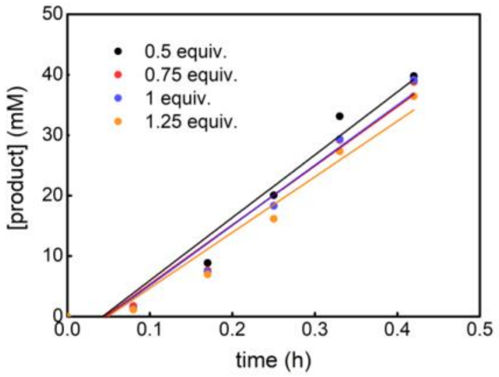

Figure S3. Plot of time (h) versus [product] (mM) with different concentrations of oxidant with ligand **L1** to determine the rate of the reactions.

Table S15. Data used to determine the order of oxidant with ligand **L1**.

Entry	[oxidant] (mM)	*k* (mM/h)	R^2	log[oxidant] (mM)	log*k* (mM/h)
1	83.33	103.99	0.9583	1.92	2.02
2	125.00	97.96	0.9572	2.10	1.99
3	166.67	99.31	0.9541	2.22	2.00
4	208.33	92.11	0.9511	2.32	1.96

Order of olefin with ligand L1

General procedure was followed using $Pd(OAc)_2$ (2.8 mg, 12.5 µL, 5 mol%), ligand **L1** (0.0843 M in AcOH) (150 µL, 12.5 µmol, 5 mol%), *tert*-butyl peroxybenzoate (47 µL, 0.25 mmol, 1 equiv), ethyl acrylate (0.5–1.5 equiv), benzene (0.25 mL, 2.8 mmol, 11.2 equiv) and AcOH (1.1 mL, 0.2 M). The reaction was sampling at 5, 10, 15, 20 and 25 min.

Table S16. Amount of olefin.

Entry	Amount of olefin	[olefin] (mM)
1	14 µL, 0.125 mmol, 0.5 equiv	83.33
2	27 µL, 0.25 mmol, 1 equiv	166.67
3	34 µL, 0.313 mmol, 1.25 equiv	208.33
4	41 µL, 0.375 mmol, 1.5 equiv	250.00

Table S17. Data used to determine the rate of the reactions with different concentrations of olefin with ligand **L1**.

Entry	[olefin] (mM)	[product] (mM)				
		0.08 h	0.17 h	0.25 h	0.33 h	0.42 h
1	83.33	1.35	9.53	20.12	33.14	42.25
2	166.67	1.20	9.34	18.05	30.97	42.25
3	208.33	1.03	7.86	16.46	25.77	35.76
4	250.00	1.55	6.83	17.11	25.43	34.36

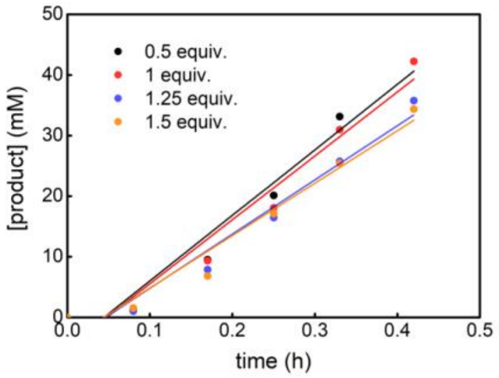

Figure S4. Plot of time (h) versus [product] (mM) with different concentrations of olefin with ligand **L1** to determine the rate of the reactions.

Table S18. Data used to determine the order of olefin with ligand **L1**.

Entry	[olefin] (mM)	*k* (mM/h)	R^2	log[olefin] (mM)	log*k* (mM/h)
1	83.33	108.20	0.9612	1.92	2.03
2	166.67	105.56	0.9553	2.22	2.02
3	208.33	89.32	0.9607	2.32	1.95
4	250.00	85.56	0.9605	2.40	1.93

Order of NaOAc with ligand L1

General procedure was followed using $Pd(OAc)_2$ (2.8 mg, 12.5 µL, 5 mol%), ligand **L1** (0.0843 M in AcOH) (150 µL, 12.5 µmol, 5 mol%), *tert*-butyl peroxybenzoate (47 µL, 0.25 mmol, 1 equiv), ethyl acrylate (27 µL, 0.25 mmol, 1 equiv), benzene (0.25 mL, 2.8 mmol, 11.2 equiv), NaOAc (0.5–2.5 equiv) and AcOH (1.1 mL, 0.2 M). The reaction was sampling at 5, 10, 15, 20 and 25 min.

Table S19. Amount of NaOAc.

Entry	Amount of NaOAc	[NaOAc] (mM)
1	10.3 mg, 0.125 mmol, 0.5 equiv	83.33
2	20.5 mg, 0.25 mmol, 1 equiv	166.67
3	30.8 mg, 0.375 mmol, 1.5 equiv	250.00
4	41.0 mg, 0.5 mmol, 2 equiv	333.33
5	51.3 mg, 0.625 mmol, 2.5 equiv	416.67

Table S20. Data used to determine the rate of the reactions with different concentrations of NaOAc with ligand **L1**.

Entry	[NaOAc] (mM)	[product] (mM)				
		0.08 h	0.17 h	0.25 h	0.33 h	0.42 h
1	83.33	3.81	13.38	20.99	28.27	35.10
2	166.67	2.33	6.85	9.55	14.69	18.55
3	250.00	2.09	5.47	8.49	13.14	15.86
4	333.33	1.80	4.89	7.06	10.07	13.32
5	416.67	1.68	3.74	6.50	9.34	10.21

Figure S5. Plot of time (h) versus [product] (mM) with different concentrations of NaOAc with ligand **L1** to determine the rate of the reactions.

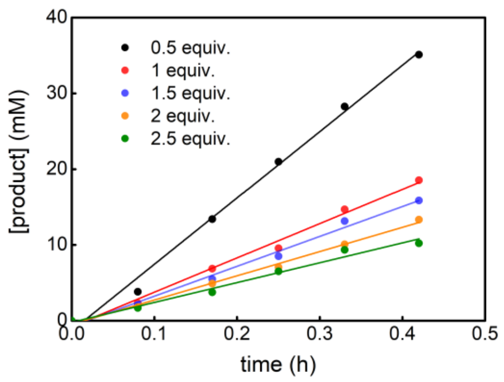

Table S21. Data used to determine the order of NaOAc with ligand **L1**.

Entry	[NaOAc] (mM)	*k* (mM/h)	R^2	log[NaOAc] (mM)	log*k* (mM/h)
1	83.33	87.56	0.9936	1.92	1.94
2	166.67	45.24	0.9912	2.22	1.66
3	250.00	39.39	0.9894	2.40	1.60
4	333.33	31.96	0.9948	2.52	1.50
5	416.67	26.17	0.9821	2.62	1.42

Order of catalyst without ligand

General procedure was followed using $Pd(OAc)_2$ (1–10 mol%), *tert*-butyl peroxybenzoate (47 µL, 0.25 mmol, 1 equiv), ethyl acrylate (27 µL, 0.25 mmol, 1 equiv), benzene (0.25 mL, 0.28 mmol, 11.2 equiv) and AcOH (1.25 mL, 0.2 M). The reaction was sampling at 30, 45, 60, 75 and 90 min.

Table S22. Amount of catalyst.

Entry	Amount of $Pd(OAc)_2$	[catalyst] (mM)
1	0.6 mg, 2.50 µmol, 1 mol%	1.67
2	1.4 mg, 6.25 µmol, 2.5 mol%	4.17
3	2.8 mg, 12.50 µmol, 5 mol%	8.33
4	4.2 mg, 18.75 µmol, 7.5 mol%	12.50
5	5.6 mg, 25.00 µmol, 10 mol%	16.67

Table S23. Data used to determine the rate of the reactions with different concentrations of catalyst without ligand.

Entry	[catalyst] (mM)	[product] (mM)				
		0.5 h	0.75 h	1 h	1.25 h	1.5 h
1	1.67	4.47	5.90	7.62	11.44	12.86
2	4.17	5.13	7.33	10.66	14.48	18.16
3	8.33	6.18	8.89	12.06	16.61	21.44
4	12.50	7.13	10.87	13.81	19.35	24.65
5	16.67	8.03	12.87	17.74	22.30	29.29

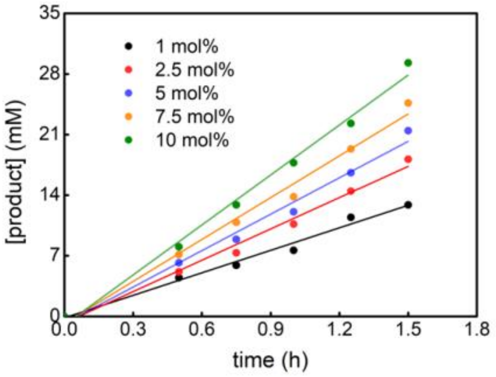

Figure S6. Plot of time (h) versus [product] (mM) with different concentrations of catalyst without ligand to determine the rate of the reactions.

Table S24. Data used to determine the order of catalyst without ligand.

Entry	[catalyst] (mM)	*k* (mM/h)	R^2	log[catalyst] (mM)	log*k* (mM/h)
1	1.67	8.66	0.9849	0.22	0.94
2	4.17	12.06	0.9878	0.62	1.08
3	8.33	14.01	0.9857	0.92	1.15
4	12.50	16.13	0.9876	1.10	1.21
5	16.67	19.22	0.9917	1.22	1.28

Order of benzene without ligand

General procedure was followed using $Pd(OAc)_2$ (2.8 mg, 12.5 µL, 5 mol%), *tert*-butyl peroxybenzoate (47 µL, 0.25 mmol, 1 equiv), ethyl acrylate (27 µL, 0.25 mmol, 1 equiv), benzene (6.7–24.7 equiv), C_6F_6 and AcOH (1.1 mL, 0.2 M). The reaction was sampling at 30, 45, 60, 75 and 90 min.

Table S25. Amount of benzene.

Entry	Amount of benzene	Amount of C_6F_6	[benzene] (mM)
1	0.15 mL, 1.675 mmol, 6.7 equiv	0.6 mL	837.50
2	0.25 mL, 2.8 mmol, 11.2 equiv	0.5 mL	1400.00
3	0.35 mL, 3.925 mmol, 15.7 equiv	0.4 mL	1962.50
4	0.45 mL, 5.05 mmol, 20.2 equiv	0.3 mL	2525.00

Table S26. Data used to determine the rate of the reactions with different concentrations of benzene without ligand.

Entry	[benzene] (mM)	[product] (mM)				
		0.5 h	0.75 h	1 h	1.25 h	1.5 h
1	837.50	1.94	2.88	3.98	4.67	5.57
2	1400.00	3.49	5.07	7.44	9.78	11.40
3	1962.50	4.35	7.34	9.37	12.27	16.52
4	2525.00	6.28	8.57	11.95	14.92	19.30

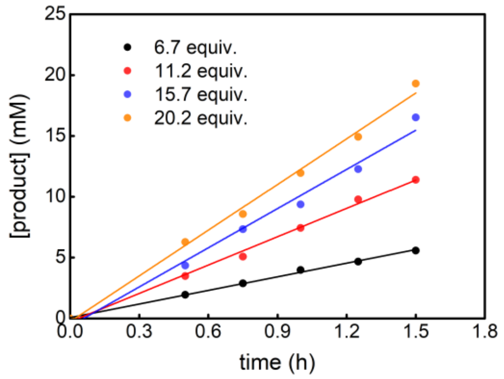

Figure S7. Plot of time (h) versus [product] (mM) with different concentrations of benzene without ligand to determine the rate of the reactions.

Table S27. Data used to determine the order of benzene without ligand.

Entry	[benzene] (mM)	*k* (mM/h)	R^2	log[benzene] (mM)	log*k* (mM/h)
1	837.50	3.73	0.9976	2.92	0.57
2	1400.00	7.77	0.995	3.15	0.89
3	1962.50	10.72	0.9855	3.29	1.03
4	2525.00	12.53	0.994	3.40	1.10

Order of oxidant without ligand

General procedure was followed using $Pd(OAc)_2$ (2.8 mg, 12.5 μL, 5 mol%), *tert*-butyl peroxybenzoate (0.5–2.5 equiv), ethyl acrylate (27 μL, 0.25 mmol, 1 equiv), benzene (0.25 mL, 2.8 mmol, 11.2 equiv) and AcOH (1.1 mL, 0.2 M). The reaction was sampling at 30, 45, 60, 75 and 90 min.

Table S28. Amount of oxidant.

Entry	Amount of oxidant	[oxidant] (mM)
1	24 μL, 0.125 mmol, 0.5 equiv	83.33
2	47 μL, 0.25 mmol, 1 equiv	166.67
3	71 μL, 0.375 mmol, 1.5 equiv	250.00
4	94 μL, 0.5 mmol, 2 equiv	333.33
5	118 μL, 0.625 mmol, 2.5 equiv	416.67

Table S29. Data used to determine the rate of the reactions with different concentrations of oxidant without ligand.

Entry	[oxidant] (mM)	[product] (mM)				
		0.5 h	0.75 h	1 h	1.25 h	1.5 h
1	83.33	7.04	13.39	19.86	27.17	34.91
2	166.67	7.06	13.29	19.70	25.51	33.94
3	250.00	6.73	11.66	17.98	23.73	30.85
4	333.33	6.26	10.57	14.82	19.70	24.46
5	416.67	6.71	10.51	15.61	20.45	25.61

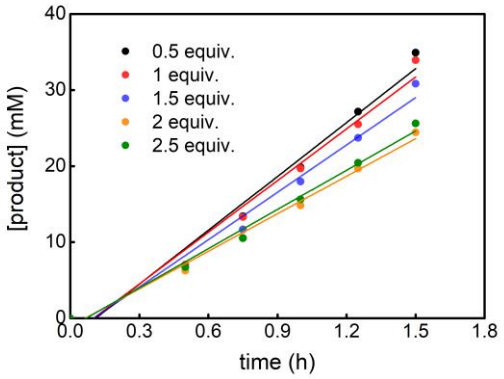

Figure S8. Plot of time (h) versus [product] (mM) with different concentrations of oxidant without ligand to determine the rate of the reactions.

Table S30. Data used to determine the order of oxidant without ligand.

Entry	[oxidant] (mM)	*k* (mM/h)	R^2	log[oxidant] (mM)	log*k* (mM/h)
1	83.33	23.62	0.9758	1.92	1.37
2	166.67	22.68	0.9780	2.22	1.36
3	250.00	20.73	0.9784	2.40	1.32
4	333.33	16.47	0.9912	2.52	1.22
5	416.67	17.20	0.9896	2.62	1.24

Order of olefin without ligand

General procedure was followed using $Pd(OAc)_2$ (2.8 mg, 12.5 µL, 5 mol%), *tert*-butyl peroxybenzoate (47 µL, 0.25 mmol, 1 equiv), ethyl acrylate (0.5–1.5 equiv), benzene (0.25 mL, 2.8 mmol, 11.2 equiv) and AcOH (1.1 mL, 0.2 M). The reaction was sampling at 30, 45, 60, 75 and 90 min.

Table S31. Amount of olefin.

Entry	Amount of olefin	[olefin] (mM)
1	14 µL, 0.125 mmol, 0.5 equiv	83.33
2	20 µL, 0.188 mmol, 0.75 equiv	125.00
3	27 µL, 0.25 mmol, 1 equiv	166.67
4	34 µL, 0.313 mmol, 1.25 equiv	208.33
5	41 µL, 0.375 mmol, 1.5 equiv	250.00

Table S32. Data used to determine the rate of the reactions with different concentrations of olefin without ligand.

Entry	[olefin] (mM)	[product] (mM)				
		0.5 h	0.75 h	1 h	1.25 h	1.5 h
1	83.33	6.93	9.35	13.80	18.01	23.15
2	125.00	6.60	10.65	16.30	21.57	28.10
3	166.67	6.10	10.23	15.26	21.20	28.05
4	208.33	5.97	10.13	16.03	22.39	30.65
5	250.00	6.27	11.42	15.69	23.90	31.21

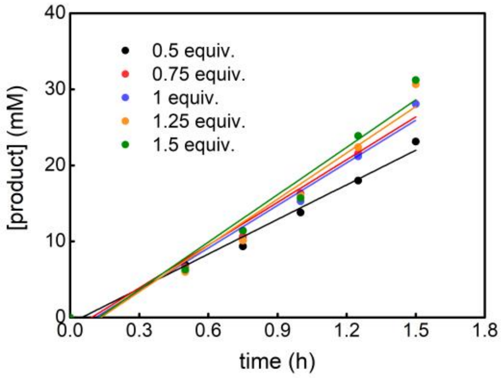

Figure S9. Plot of time (h) versus [product] (mM) with different concentrations of olefin without ligand to determine the rate of the reactions.

Table S33. Data used to determine the order of olefin without ligand.

Entry	[olefin] (mM)	*k* (mM/h)	R^2	log[olefin] (mM)	log*k* (mM/h)
1	83.33	15.18	0.9884	1.92	1.18
2	125.00	18.75	0.9802	2.10	1.27
3	166.67	18.65	0.9716	2.22	1.27
4	208.33	20.30	0.9583	2.32	1.31
5	250.00	20.80	0.9620	2.40	1.32

Order of NaOAc without ligand

General procedure was followed using $Pd(OAc)_2$ (2.8 mg, 12.5 μL, 5 mol%), *tert*-butyl peroxybenzoate (47 μL, 0.25 mmol, 1 equiv), ethyl acrylate (27 μL, 0.25 mmol, 1 equiv), benzene (0.25 mL, 2.8 mmol, 11.2 equiv), NaOAc (0.1–3.5 equiv) and AcOH (1.25 mL, 0.2 M). The reaction was sampling at 30, 45, 60, 75 and 90 min.

Table S34. Amount of NaOAc.

Entry	Amount of NaOAc	[NaOAc] (mM)
1	2.1 mg, 0.025 mmol, 0.1 equiv	16.67
2	6.2 mg, 0.075 mmol, 0.3 equiv	50.00
3	10.3 mg, 0.125 mmol, 0.5 equiv	83.33
4	20.5 mg, 0.25 mmol, 1 equiv	166.67
5	30.8 mg, 0.375 mmol, 1.5 equiv	250.00
6	41.0 mg, 0.5 mmol, 2 equiv	333.33

7	51.3 mg, 0.625 mmol, 2.5 equiv	416.67
8	61.5 mg, 0.75 mmol, 3 equiv	500.00
9	71.8 mg, 0.875 mmol, 3.5 equiv	583.33

Table S35. Data used to determine the rate of the reactions with different concentrations of NaOAc without ligand.

Entry	[NaOAc] (mM)	[product] (mM)				
		0.5 h	0.75 h	1 h	1.25 h	1.5 h
1	16.67	8.16	12.66	16.19	21.96	29.78
2	50.00	10.49	16.54	22.99	39.06	41.76
3	83.33	13.48	21.06	28.64	39.15	47.95
4	166.67	17.37	24.11	32.55	43.19	50.12
5	250.00	14.93	22.67	33.76	39.31	46.06
6	333.33	9.66	13.82	18.38	23.74	27.14
7	416.67	7.88	12.35	14.86	19.60	22.59
8	500.00	6.17	9.16	11.54	14.31	17.44
9	583.33	5.41	7.85	11.18	12.20	16.05

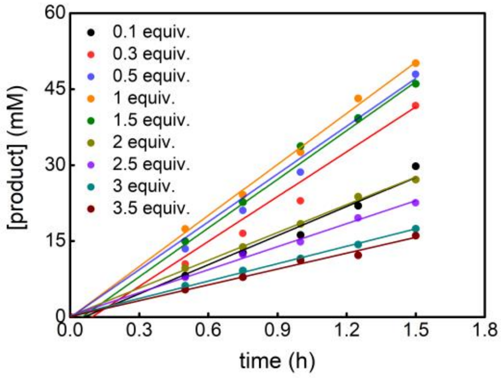

Figure S10. Plot of time (h) versus [product] (mM) with different concentrations of NaOAc without ligand to determine the rate of the reactions.

Table S36. Data used to determine the order of NaOAc without ligand.

Entry	[NaOAc] (mM)	*k* (mM/h)	R^2	log[NaOAc] (mM)	log*k* (mM/h)
1	16.67	19.15	0.9807	1.22	1.28
2	50.00	29.53	0.9575	1.70	1.47
3	83.33	32.10	0.9926	1.92	1.51
4	166.67	33.62	0.9976	2.22	1.53
5	250.00	31.43	0.9945	2.40	1.50
6	333.33	18.29	0.9982	2.52	1.26
7	416.67	15.12	0.9964	2.62	1.18
8	500.00	11.45	0.9985	2.70	1.06
9	583.33	10.42	0.9911	2.77	1.02

Procedure for the reaction using an excess of NaOAc with the aim to detect [L2Pd(OAc)$_2$]$^-$ (the reaction is performed in an NMR tube)

$Pd(OAc)_2$ (2.2 mg, 0.01 mmol, 1 equiv), **L2** (2.0 mg, 0.01 mmol, 1 equiv) and AcOD-d_4 (0.5 mL) were added into an NMR tube. The NMR tube was sealed with a screw cap and the reaction was placed in a 100 °C pre-heated oil bath for 20 minutes until solid was dissolved. The reaction was cooled down to room temperature and the ^{1}H NMR spectrum was collected. Then, NaOAc (16.4 mg, 0.2 mmol, 20 equiv) was added to the NMR tube. The NMR tube was again placed in a 100 °C pre-heated oil bath for 2 h. The reaction was cooled down to room temperature and the ^{1}H NMR spectrum was collected.

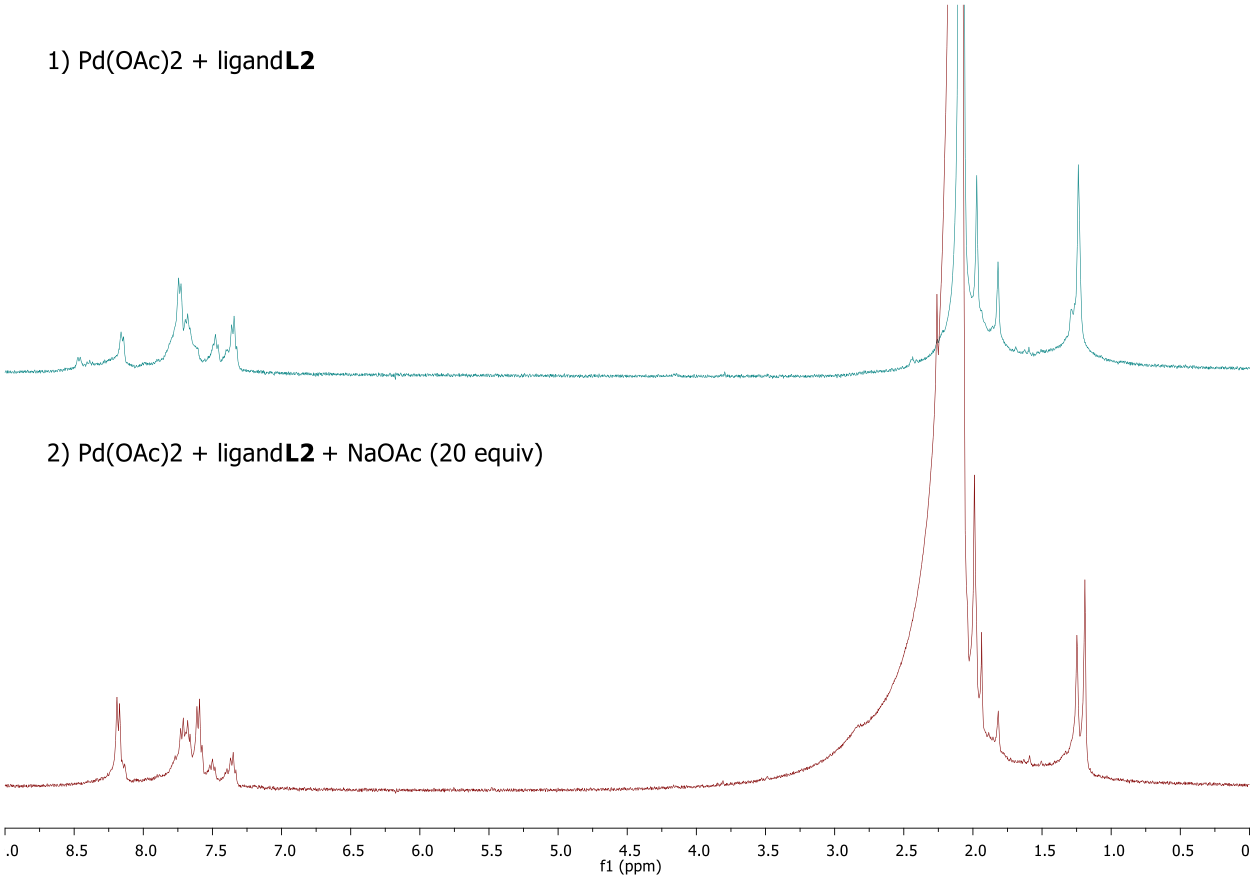

Figure S11. ^{1}H NMR spectra of the mixture of $Pd(OAc)_2$ and ligand **L2** in AcOD-d_4, 1) before and 2) after the addition of 20 equiv of NaOAc.

KIE for Pd-catalyzed C–H olefination of benzene with ligand L1

$Pd(OAc)_2$ (5.6 mg, 25.0 µmol, 5 mol%), a stock solution of ligand **L1** (0.0846 M in AcOH) (300 µL, 25.0 µmol, 5 mol%), *tert*-butyl peroxybenzoate (94 µL, 0.5 mmol, 1 equiv), ethyl acrylate (53 µL, 0.5 mmol, 1 equiv), benzene (0.5 mL, 5.6 mmol, 11.2 equiv) or benzene-d_6 (0.5 mL, 5.6 mmol, 11.2 equiv) and AcOH (2.2 mL, 0.2 M) were added into a pressure tube. The pressure tube was sealed with a crimp cap with septa and the reaction was placed in a 100 °C pre-heated oil bath. The reaction was followed during the time (5, 10, 15, 30 and 45 min) by sampling 100 µL. PhCl (20 µL) was added in each sample as an internal standard for quantitative GC analysis. The reaction mixture was diluted with EtOAc (0.5 mL). The organic layer was quenched with saturated aqueous $NaHCO_3$ solution (0.5 mL). The organic layer was filtered through a plug of anh. $MgSO_4$ and analyzed by GC.

Table S37. Data used to determine KIE for C–H olefination of benzene with ligand **L1**.

**Entry**	**Time (h)**	**[product] (mM)**	
		**Benzene**	**Benzene-d$_6$**
1	0.08	2.63	1.10
2	0.17	10.16	2.93
3	0.25	19.39	5.50
4	0.50	47.31	12.60
5	0.75	71.08	17.60

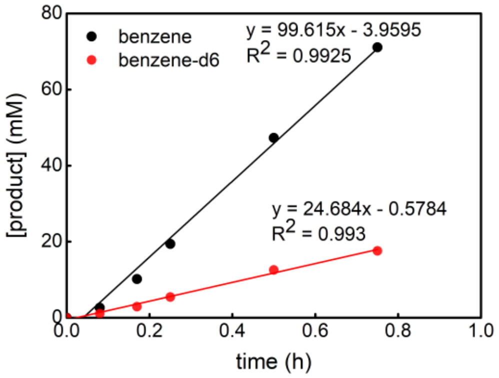

Figure S12. Plot of time (h) versus [product] (mM) to determine KIE for C–H olefination of benzene with ligand **L1**.

<u>**KIE for Pd-catalyzed C–H olefination of benzene without ligand**</u>

$Pd(OAc)_2$ (5.6 mg, 25.0 µmol, 5 mol%), *tert*-butyl peroxybenzoate (94 µL, 0.5 mmol, 1 equiv), ethyl acrylate (53 µL, 0.5 mmol, 1 equiv), benzene (0.5 mL, 5.6 mmol, 11.2 equiv) or benzene-d$_6$ (0.5 mL, 5.6 mmol, 11.2 equiv) and AcOH (2.5 mL, 0.2 M) were added into a pressure tube. The pressure tube was sealed with a crimp cap with septa and the reaction was placed in a 100 °C pre-heated oil bath. The reaction was followed during the time (5, 10, 15, 30 and 45 min) by sampling 100 µL. PhCl (20 µL) was added in each sample as an internal standard for quantitative GC analysis. The reaction mixture was diluted with EtOAc (0.5 mL). The organic layer was quenched with saturated aqueous $NaHCO_3$ solution (0.5 mL). The organic layer was filtered through a plug of anh. $MgSO_4$ and analyzed by GC.

Table S38. Data used to determine KIE for C–H olefination of benzene without ligand.

**Entry**	**Time (h)**	**[product] (mM)**	
		**Benzene**	**Benzene-d$_6$**
1	0.08	0.99	0.74
2	0.17	2.17	1.13
3	0.25	2.77	1.25
4	0.50	7.03	1.82
5	0.75	12.85	2.66

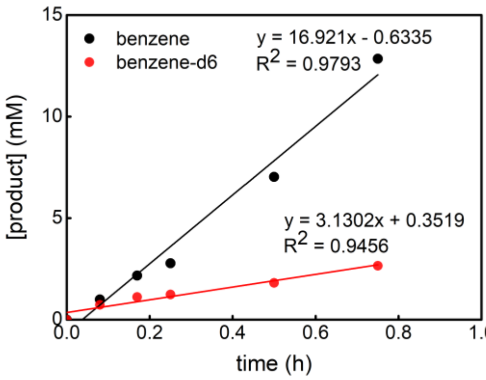

Figure S13. Plot of time (h) versus [product] (mM) to determine KIE for C–H olefination of benzene without ligand.

Procedure for one-pot intermolecular competition experiment of *p*-xylene and 1,4-bis(trifluoromethyl)benzene with ligand L1

$Pd(OAc)_2$ (2.8 mg, 12.5 µmol, 5 mol%), a stock solution of ligand **L1** (0.0846 M in DCE) (150 µL, 12.5 µmol, 5 mol%), *tert*-butyl peroxybenzoate (47 µL, 0.25 mmol, 1 equiv), ethyl acrylate (27 µL, 0.25 mmol, 1 equiv), *p*-xylene (0.15 mL, 1.25 mmol, 5 equiv), 1,4-bis(trifluoromethyl)benzene (0.19 mL, 1.25 mmol, 5 equiv) and AcOH (1.10 mL, 0.2 M) were added into a pressure tube. The pressure tube was sealed with a screw cap and the reaction was placed in a 100 °C pre-heated oil bath and stirred for 3 h. The resulting mixture was diluted with EtOAc, filtered through a plug of Celite and concentrated under reduced pressure. The ^{1}H NMR yield was determined by adding CH_2Br_2 (17.5 µL, 0.25 mmol, 1 equiv) as an internal standard. The reaction provided the olefinated product in 84% ^{1}H NMR yield (**5**:**6** = 84%:traces).

Procedure for one-pot intermolecular competition experiment of *p*-xylene and 1,4-bis(trifluoromethyl)benzene without ligand

$Pd(OAc)_2$ (2.8 mg, 12.5 µmol, 5 mol%), *tert*-butyl peroxybenzoate (47 µL, 0.25 mmol, 1 equiv), ethyl acrylate (27 µL, 0.25 mmol, 1 equiv), *p*-xylene (0.15 mL, 1.25 mmol, 5 equiv), 1,4-bis(trifluoromethyl)benzene (0.19 mL, 1.25 mmol, 5 equiv) and AcOH (1.25 mL, 0.2 M) were added into a pressure tube. The pressure tube was sealed with a screw cap and the reaction was placed in a 100 °C pre-heated oil bath and stirred for 3 h. The resulting mixture was diluted with EtOAc, filtered through a plug of Celite and concentrated under reduced pressure. The ^{1}H NMR yield was determined by adding CH_2Br_2 (17.5 µL, 0.25 mmol, 1 equiv) as an internal standard. The reaction provided the olefinated product in 22% ^{1}H NMR yield (**5**:**6** = 22%:traces).

Procedure for the H/D exchange experiment with ligand L1 (the reaction is performed in an NMR tube)

$Pd(OAc)_2$ (1.1 mg, 5.0 µmol, 5 mol%), ligand **L1** (1.1 mg, 5.0 µmol, 5 mol%), *tert*-butyl peroxybenzoate (19 µL, 0.1 mmol, 1 equiv), ethyl acrylate (11 µL, 0.1 mmol, 1 equiv), mesitylene (0.14 mL, 1.0 mmol, 10 equiv) and AcOD-d_4 (0.5 mL, 0.2 M) were mixed in a vial. The solution was transferred into an NMR tube, sealed with a screw cap and ^{1}H NMR spectrum was collected. The NMR tube was placed in a 100 °C pre-heated oil bath for 6 h. The reaction was cooled down to room temperature and the ^{1}H NMR spectrum was collected.

Procedure for the H/D exchange experiment without ligand (the reaction is performed in an NMR tube)

$Pd(OAc)_2$ (2.2 mg, 10.0 µmol, 10 mol%), *tert*-butyl peroxybenzoate (19 µL, 0.1 mmol, 1 equiv), ethyl acrylate (11 µL, 0.1 mmol, 1 equiv), mesitylene (0.14 mL, 1.0 mmol, 10 equiv) and AcOD-d_4 (0.5 mL, 0.2 M) were mixed in a vial. The solution was transferred into an NMR tube, sealed with a screw cap and ^{1}H NMR spectrum was collected. The NMR tube was placed in a 100 °C pre-heated oil bath for 16 h. The reaction was cooled down to room temperature and the ^{1}H NMR spectrum was collected.

Procedure for the reaction of ligand L2 and oxidant

Ligand **L2** (19.6 mg, 0.1 mmol, 1 equiv), *tert*-butyl peroxybenzoate (0.38 mL, 2.0 mmol, 20 equiv), benzene (2 mL, excess) and AcOH (10 mL) were added into a pressure tube. The pressure tube was sealed with a screw cap and the reaction was placed in a 100 °C pre-heated oil bath and stirred for 2 h. The reaction was cooled down to room temperature and concentrated under reduced pressure. The resulting crude was basified (2 M aqueous NaOH solution) until pH = 14. The aqueous layer was washed with CH_2Cl_2 (3 x 10 mL). The aqueous layer was acidified (6 M aqueous HCl solution) until pH = 1 and extracted with CH_2Cl_2 (3 x 15 mL). The combined organic layers were dried over anh. $MgSO_4$, filtered, concentrated under reduced pressure and analyzed by ^{1}H NMR.

Procedure for the reaction of complex 1 and oxidant

Complex **1** (15.5 mg, 0.03 mmol, 1 equiv) and *tert*-butyl peroxybenzoate (22 µL, 0.12 mmol, 4 equiv) were added into a pressure tube. The pressure tube was sealed with a screw cap and the reaction was placed in a 100 °C pre-heated oil bath and stirred for 2 h. The reaction was cooled down to room temperature, diluted with CH_2Cl_2, filtered through a plug of Celite, concentrated under reduced pressure and analyzed by ^{1}H NMR.

5. NMR spectra

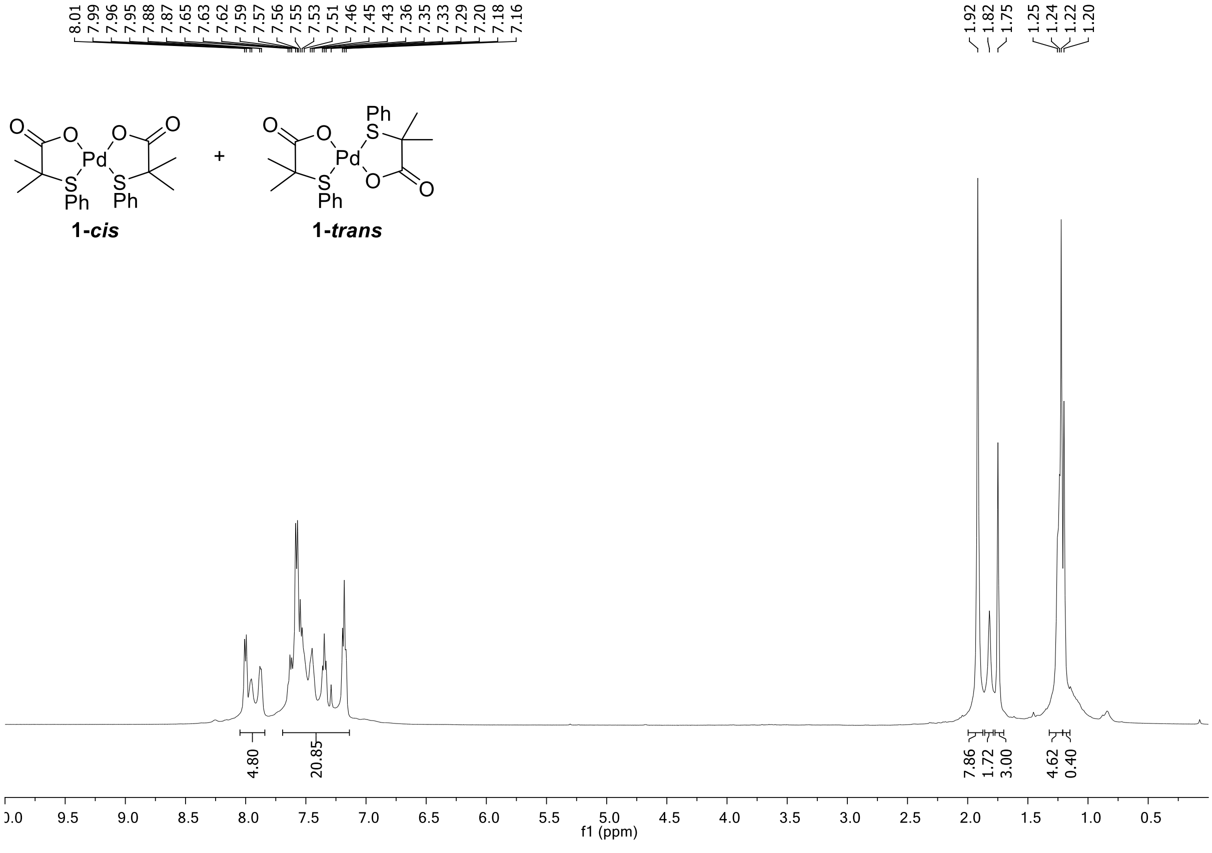

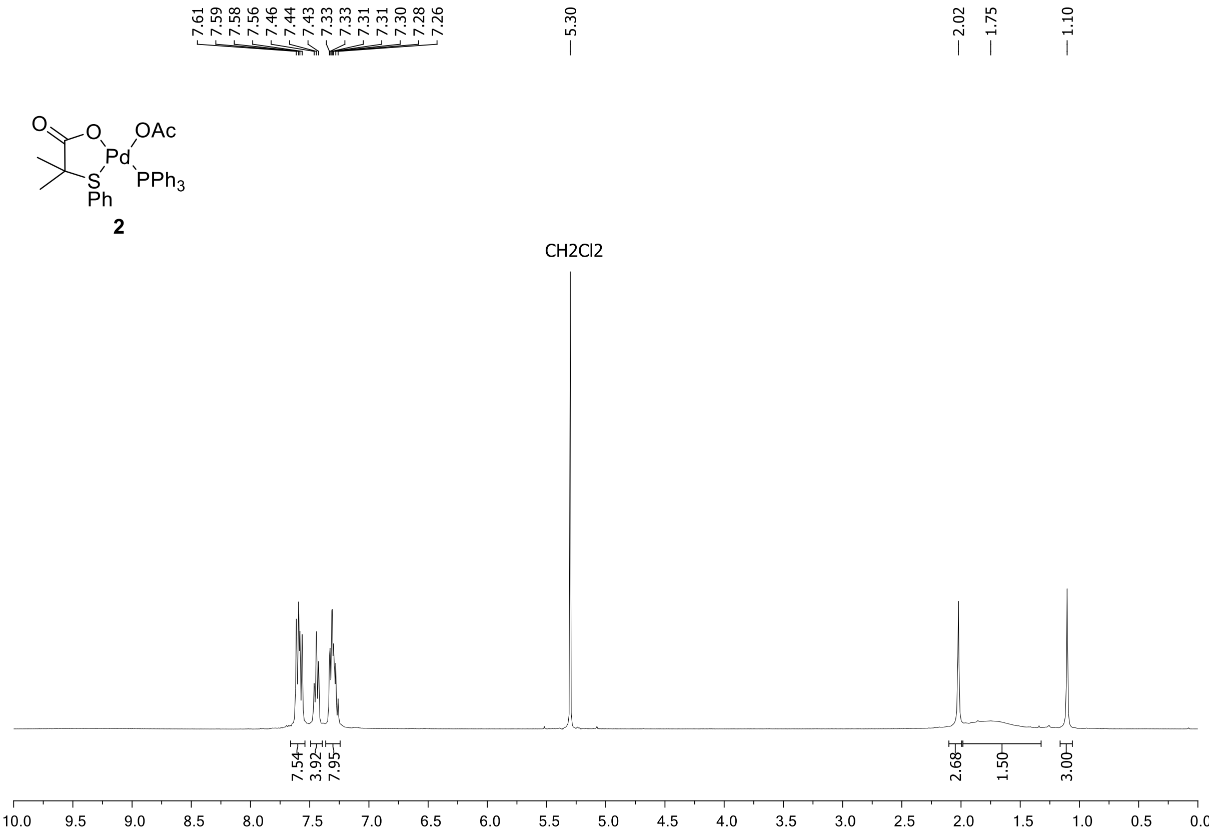
7.61 7.59 7.58 7.56 7.46 7.44 7.43 7.33 7.33 7.31 7.31 7.30 7.28 7.26
5.30
2.02
1.75
1.10
O
O
OAc
Pd
S
PPh3
Ph
2
CH2Cl2
7.54
3.92
7.95
2.68
1.50
3.00
10.0 9.5 9.0 8.5 8.0 7.5 7.0 6.5 6.0 5.5 5.0 4.5 4.0 3.5 3.0 2.5 2.0 1.5 1.0 0.5

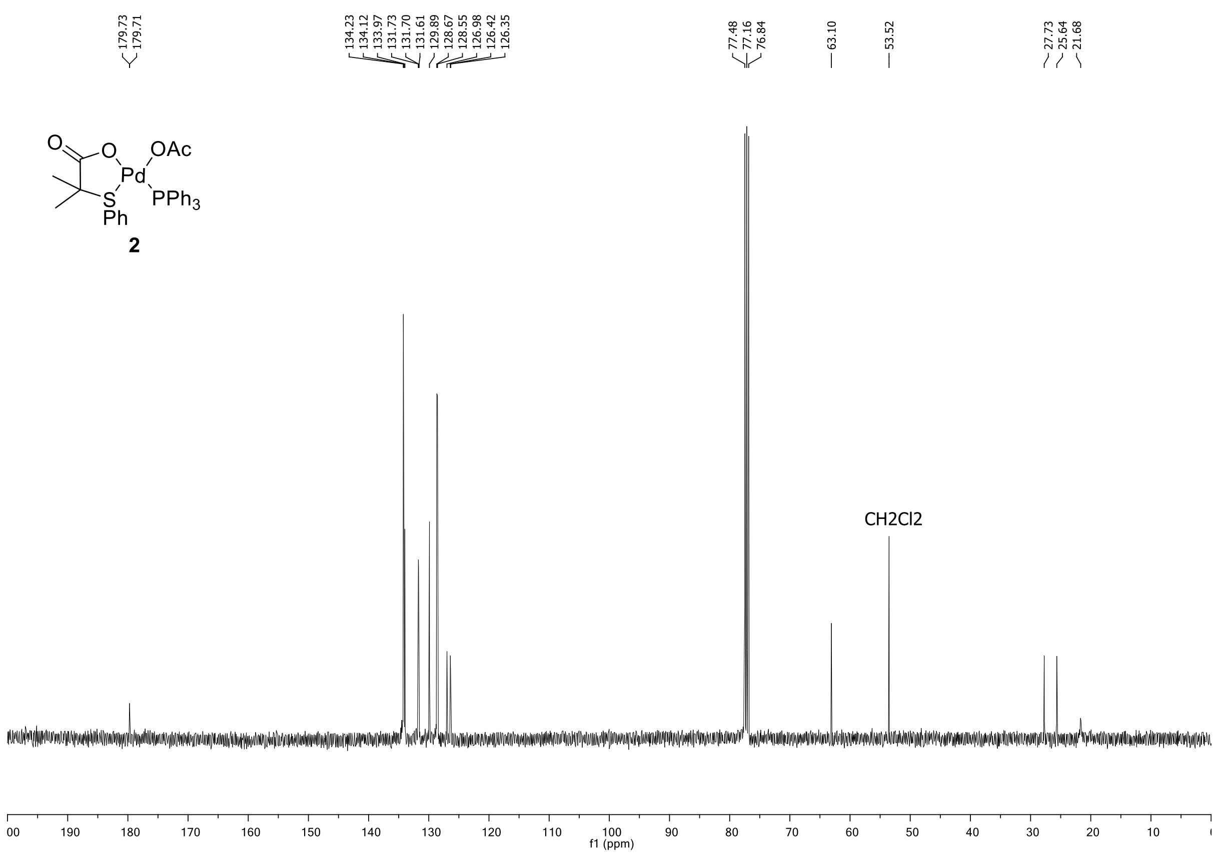
179.73 179.71
134.23 134.12 133.97 131.73 131.70 131.61 129.89 128.67 128.55 126.98 126.42 126.35
77.48 77.16 76.84
63.10
53.52
27.73 25.64 21.68
O
O
OAc
Pd
S
PPh3
Ph
2
CH2Cl2
190 180 170 160 150 140 130 120 110 100 90 80 70 60 50 40 30 20 10
f1 (ppm)

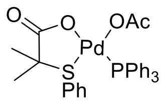
O
OAc
Pd
S
PPh3
Ph

2

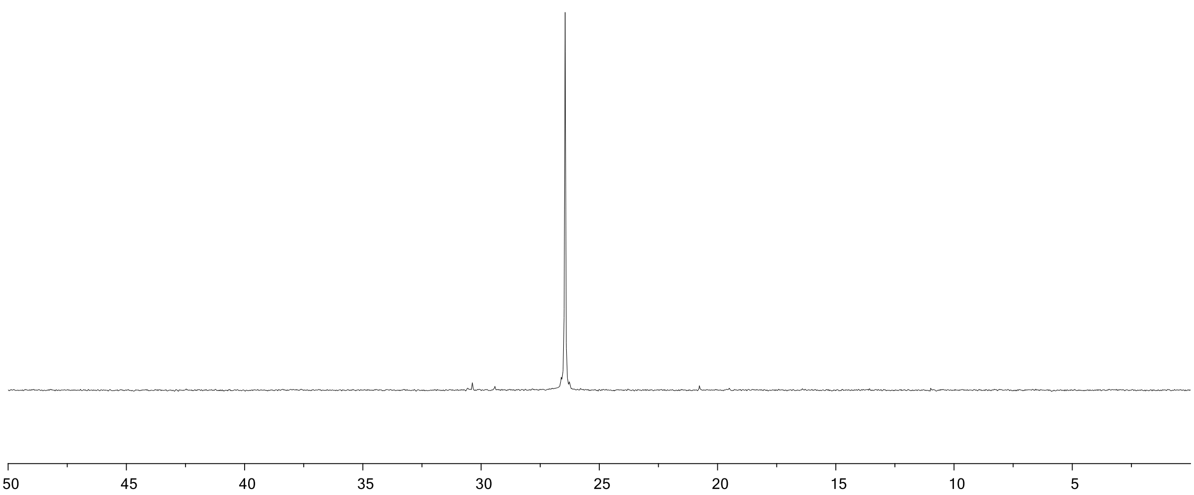
26.45
50
45
40
35
30
25
20
15
10
5

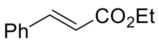
Ph
CO2Et

3-mono

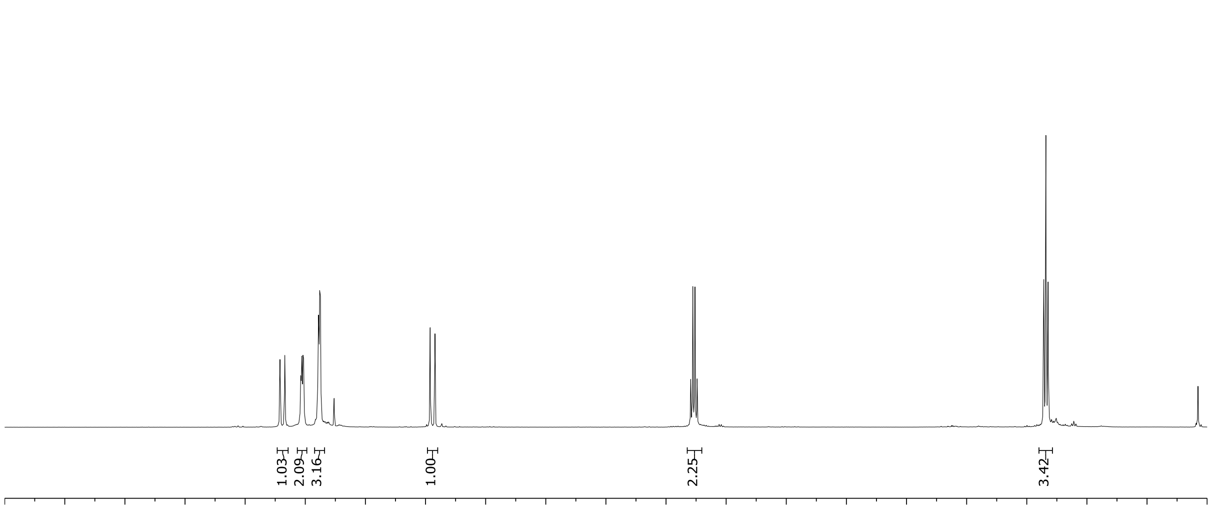
7.71
7.67
7.54
7.54
7.53
7.53
7.53
7.52
7.51
7.51
7.40
7.39
7.39
7.39
7.38
7.37
7.36
7.26
6.46
6.42
4.29
4.28
4.26
4.24
1.36
1.34
1.32
1.03
2.09
3.16
1.00
2.25
3.42

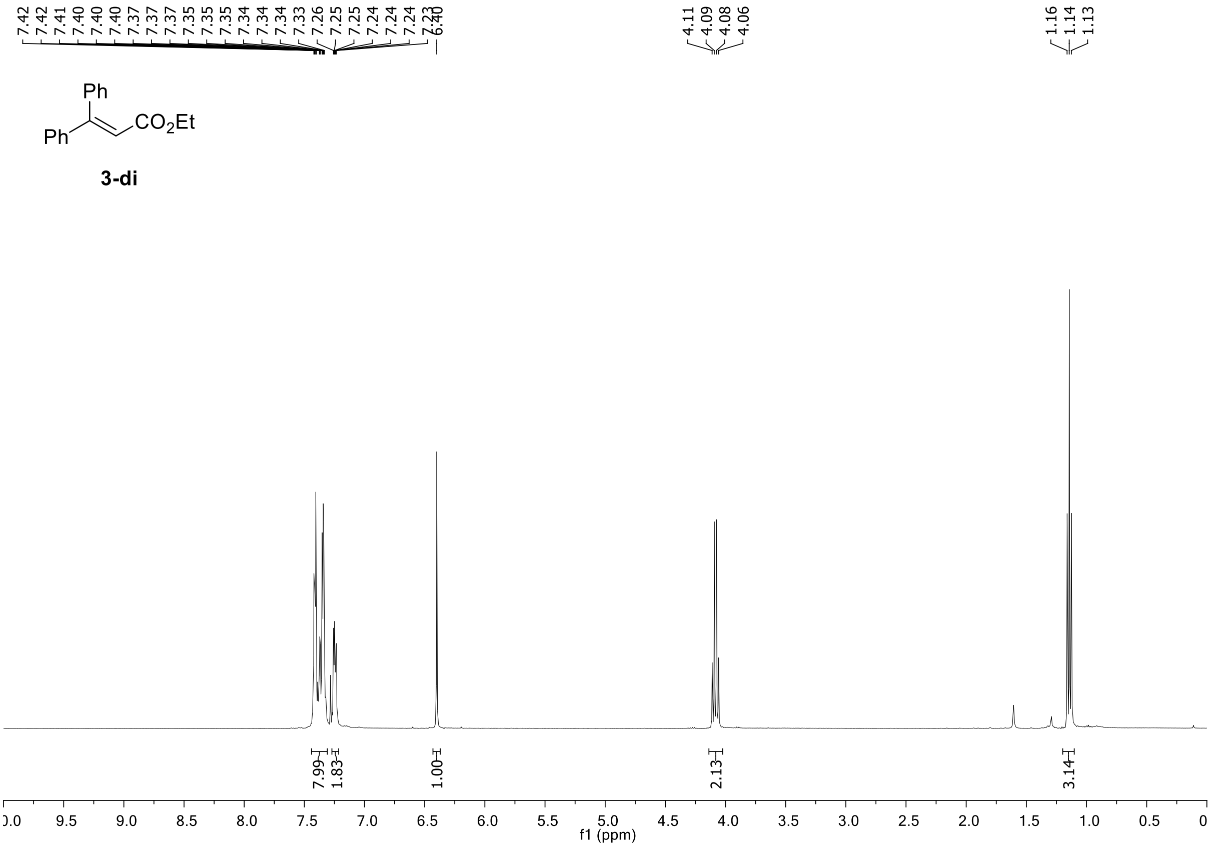
3-di
f1 (ppm)

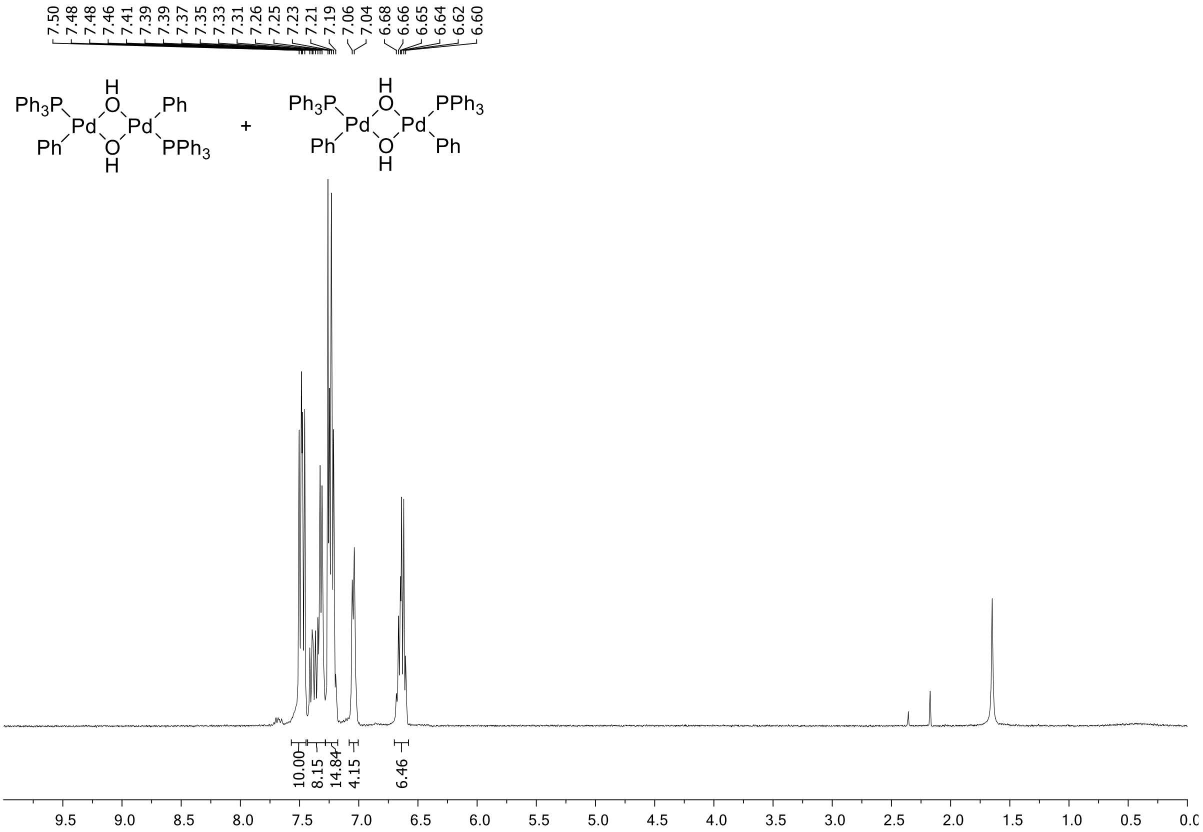
+

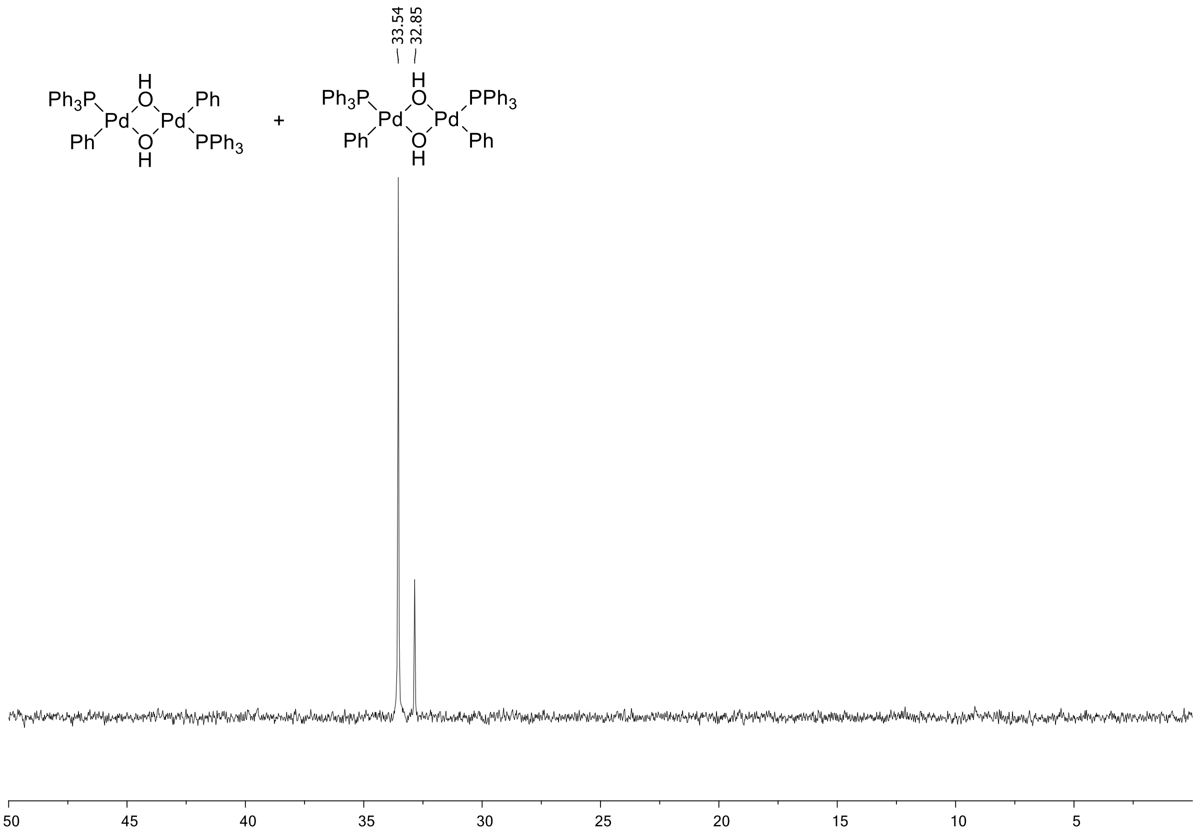
33.54
32.85
Ph3P Pd O Pd Ph
Ph O PPh3
H H
+
Ph3P Pd O Pd PPh3
Ph O Ph
50 45 40 35 30 25 20 15 10 5

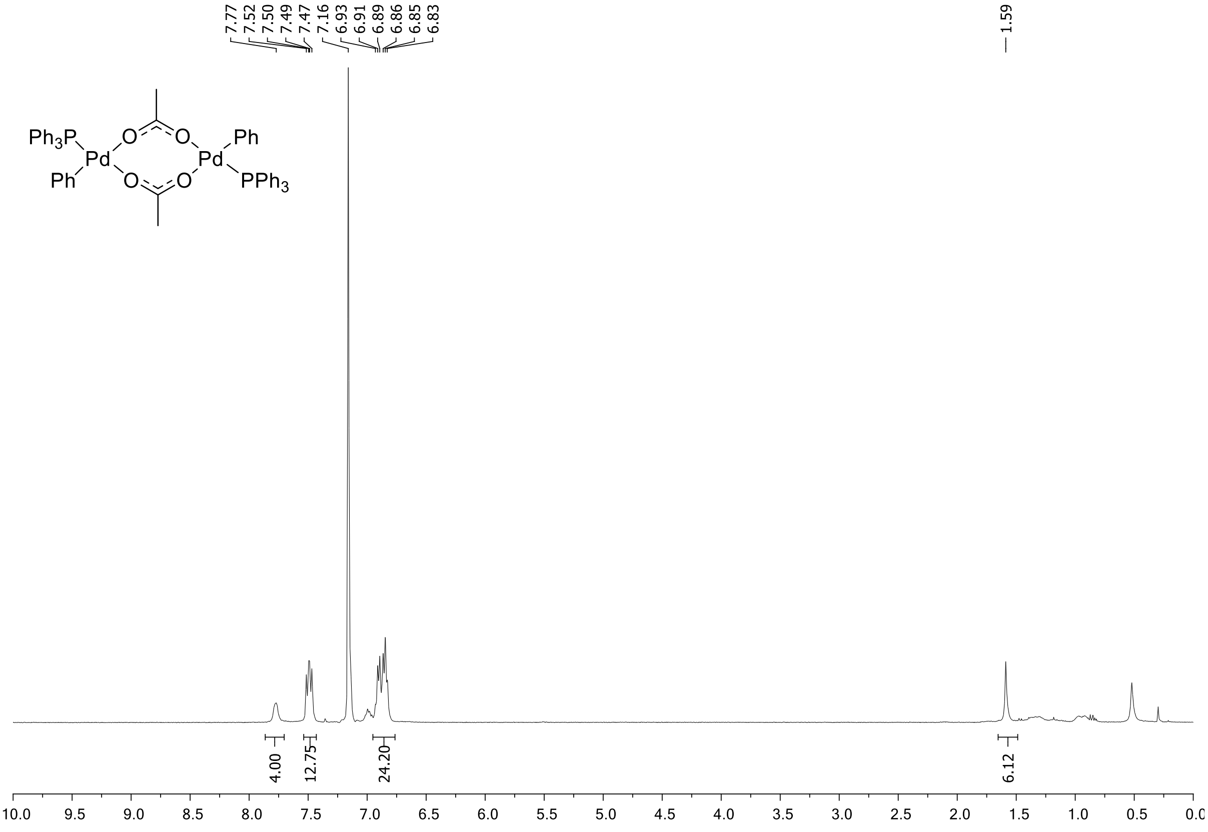
7.77 7.52 7.50 7.49 7.47 7.16 6.93 6.91 6.89 6.86 6.85 6.83
1.59
Ph3P Pd O O Ph
Ph O O Pd PPh3
4.00
12.75
24.20
6.12
10.0 9.5 9.0 8.5 8.0 7.5 7.0 6.5 6.0 5.5 5.0 4.5 4.0 3.5 3.0 2.5 2.0 1.5 1.0 0.5 0.0

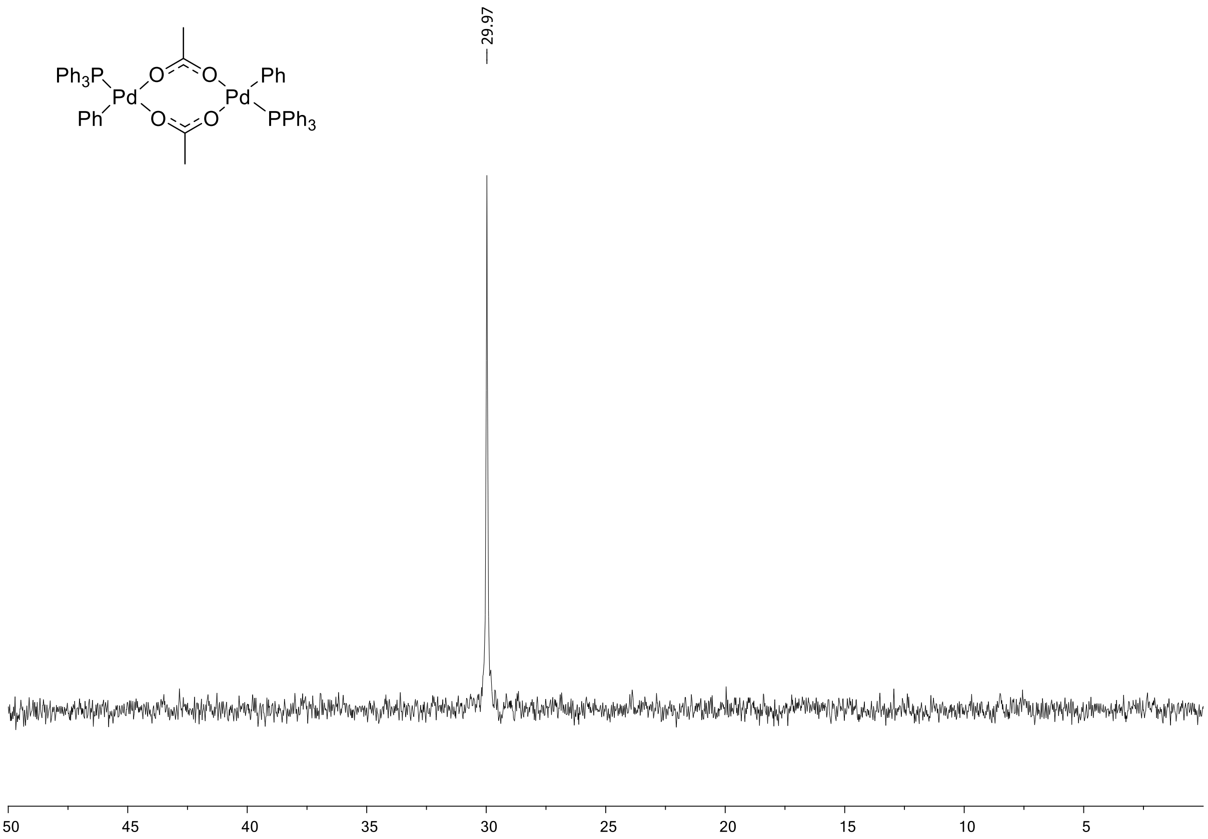
29.97
50 45 40 35 30 25 20 15 10 5

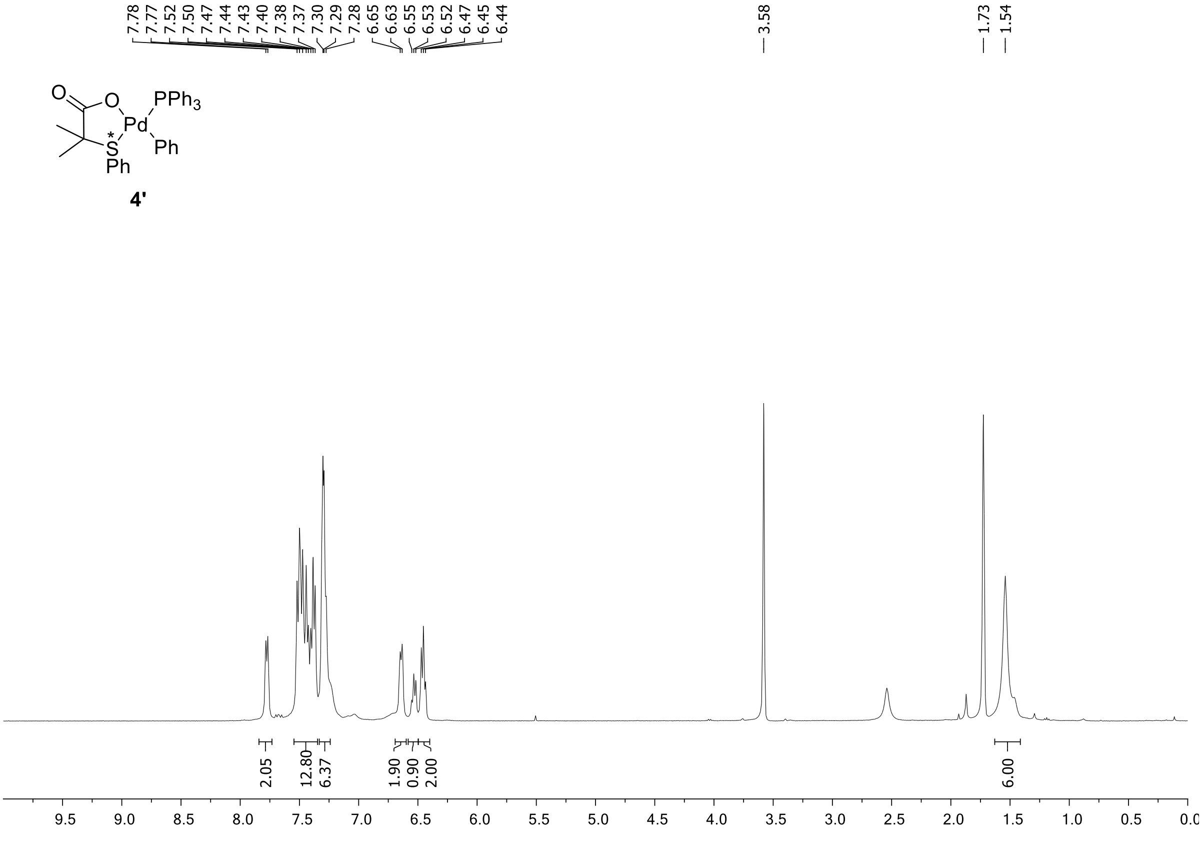
7.78 7.77 7.52 7.50 7.47 7.44 7.43 7.40 7.38 7.37 7.30 7.29 7.28 6.65 6.63 6.55 6.53 6.52 6.47 6.45 6.44
3.58
1.73 1.54
4'
2.05 12.80 6.37 1.90 0.90 2.00 6.00
9.5 9.0 8.5 8.0 7.5 7.0 6.5 6.0 5.5 5.0 4.5 4.0 3.5 3.0 2.5 2.0 1.5 1.0 0.5

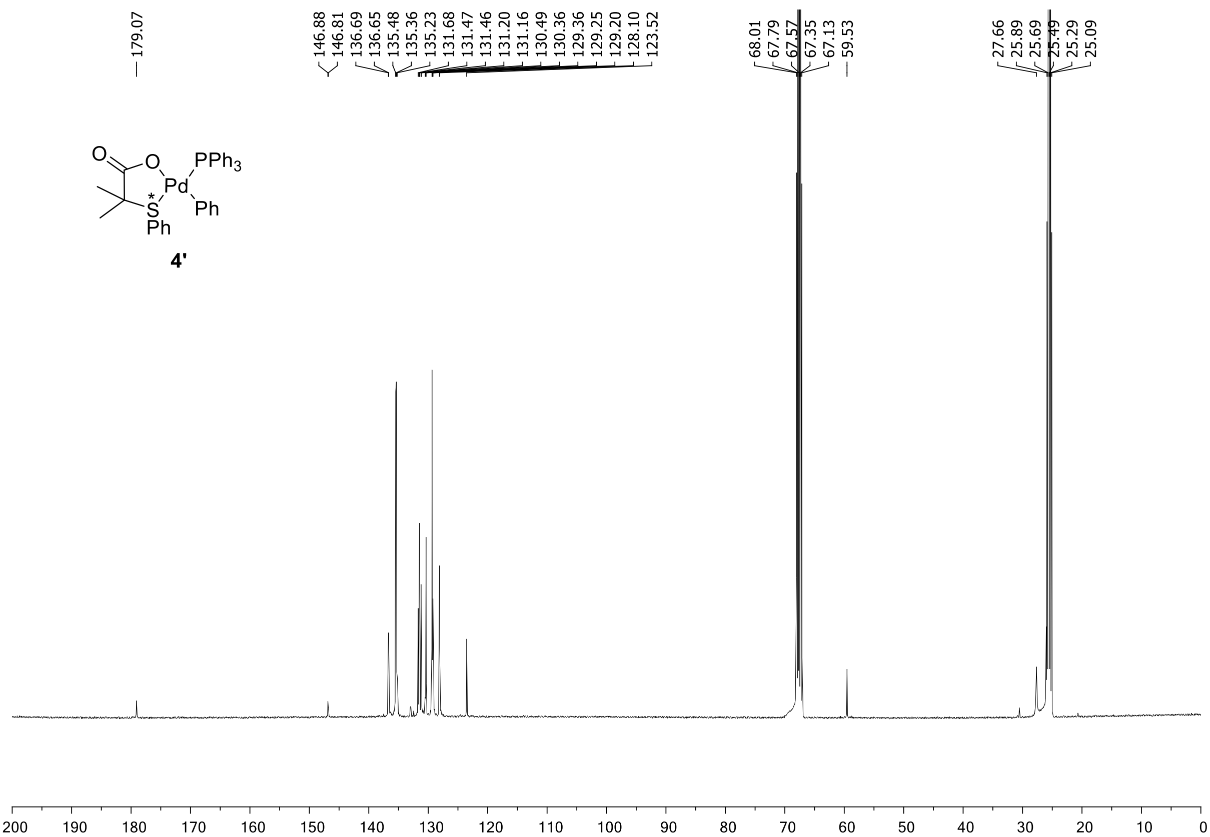
179.07
146.88
146.81
136.69
136.65
135.48
135.36
135.23
131.68
131.47
131.46
131.20
131.16
130.49
130.36
129.36
129.25
129.20
128.10
123.52
68.01
67.79
67.57
67.35
67.13
59.53
27.66
25.89
25.69
25.49
25.29
25.09
O
O
PPh3
Pd
S
Ph
Ph
4'
200 190 180 170 160 150 140 130 120 110 100 90 80 70 60 50 40 30 20 10 0

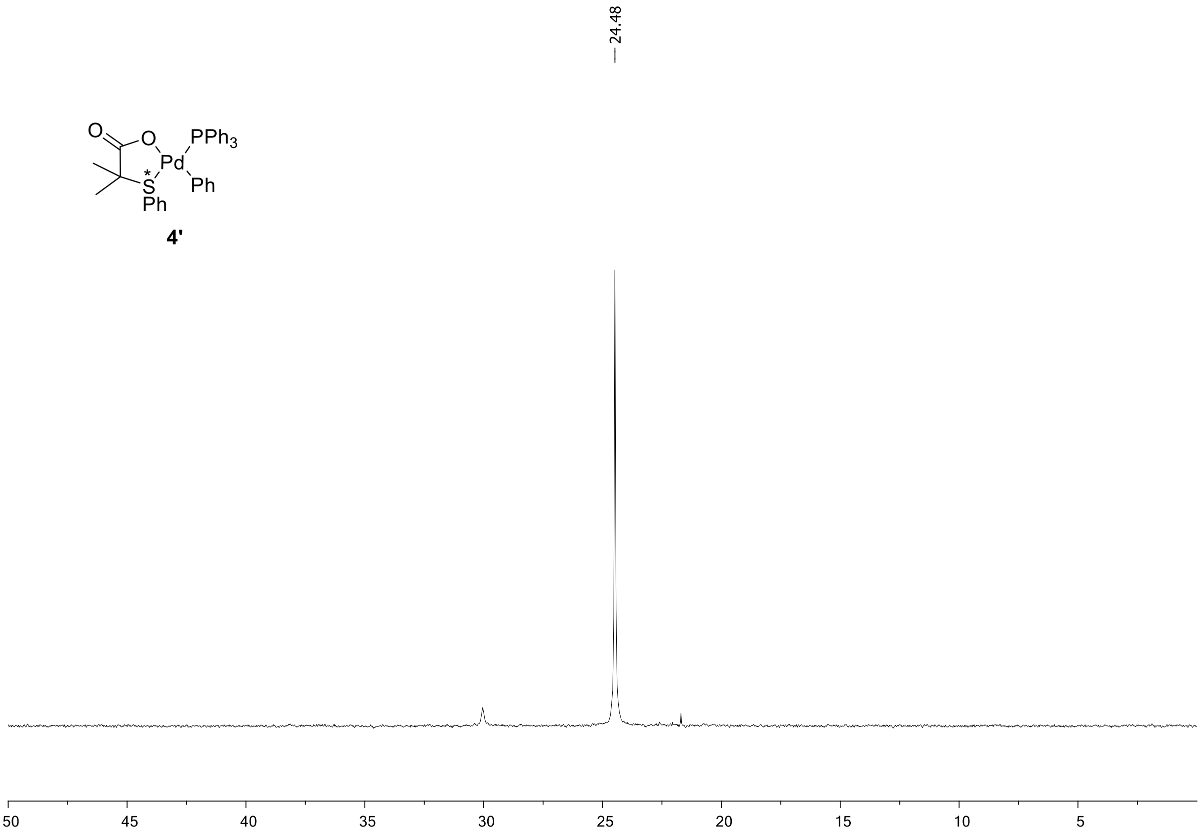
24.48
O
O
PPh3
Pd
S
Ph
Ph
4'
50 45 40 35 30 25 20 15 10 5

6. Mass spectrometric measurements

The mass spectra were recorded on a linear ion trap (LTQ) instrument with an electrospray ionization (ESI) source.[5] General conditions used were as follows: 4 to 5 kV spray voltage, 200 to 275 °C capillary temperature and 1 to 20 psi sheath gas. For positive mode, 0 to 20 V capillary voltage and 25 to 70 V tube lens while for the negative mode 0 to -20 V capillary voltage and -25 to -75 V tube lens.

Energy resolved collision induced dissociation (CID) experiments were performed on LCQ Deca mass spectrometer with an ESI source.[6] The calibration was performed using the thermometer ions using the Schroder's method to correlate the collision energy and the appearance energies of the ions.[7]

In general, 1 mM fresh stock solution was prepared in the desired solvent (sonication used if turbidity observed followed by filtration, filtrate used). From these stock solutions after mixing/addition, by appropriate dilution with the desired solvent final concentration of 50 to 200 µM was injected directly into the ESI–MS inlet with the help of a silica capillary using the nitrogen overpressure in the vial.

[Pd(**L2**)(PPh_2Ph^{SO3})OAc]Na i.e. the negatively charge tagged analog of 'complex **2**' was synthesized according to the "Procedure for the synthesis of complex **2**" described above with the exception that instead of triphenylphosphine, 3-(diphenylphosphino)benzenesulfonic acid sodium salt i.e. PPh_2Ph^{SO3}Na was used and dark yellowish solid of [Pd(**L2**)(PPh_2Ph^{SO3})OAc]Na was obtained. This was then used further for the mass spectrometry analysis.

High resolution mass spectra were recorded with a timsTOF instrument from Bruker Daltonik (Bremen, Germany) equipped with an ESI source. Calibration was performed using an Agilent ESI low concentration tune mix before the experiment. The sample and the calibration solutions were injected in the timsTOF using a glass syringe pump with 0.5 mL volume and flow rate of 0.3 µL. The timsTOF was operated in the negative ion mode in a mass range of *m/z* 50 to 1500 with the spray voltage of 4.5 kV. The end plate offset of -500 V with a N_2 nebulizer pressure of 0.3 bar and a dry gas flow of 1.5 L min^{-1} at 275 °C.

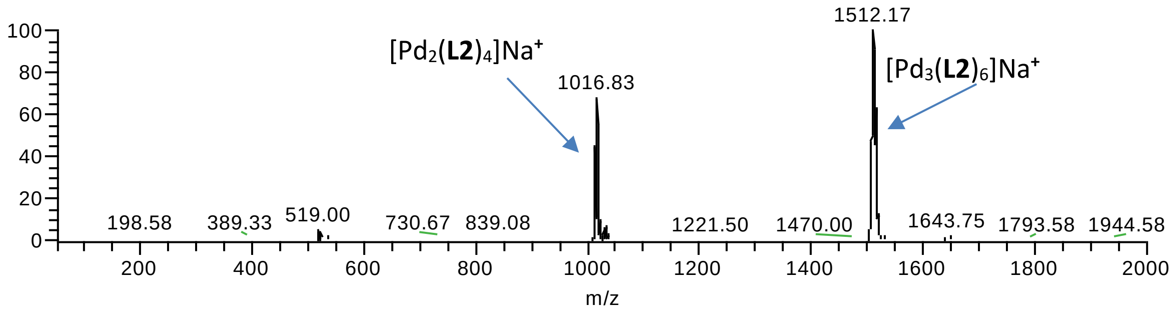

Figure S14. ESI mass spectrum (positive mode) of a mixture of $Pd(OAc)_2$ and **L2** in benzene and ethyl acetate.

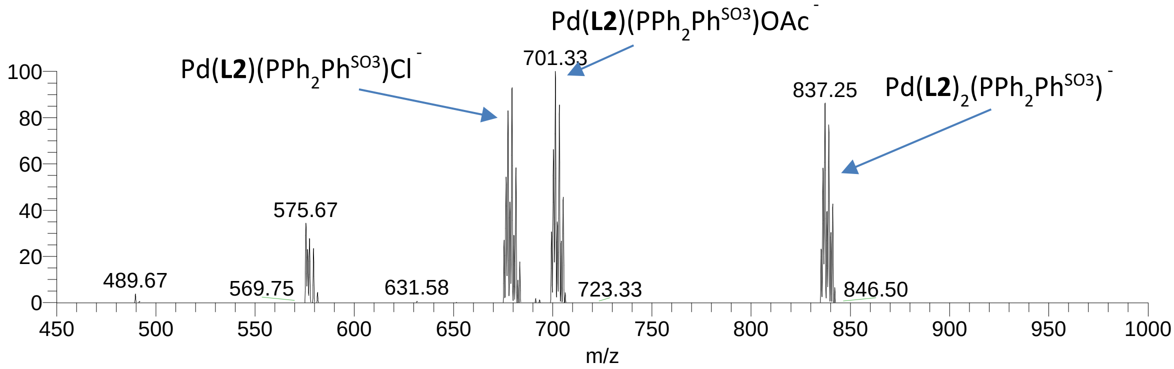

Figure S15. ESI mass spectrum (negative mode) of a mixture of $Pd(OAc)_2$, **L2**, $(PPh_2Ph^{SO3-})Na$ and acetic acid in benzene and deuterated benzene.

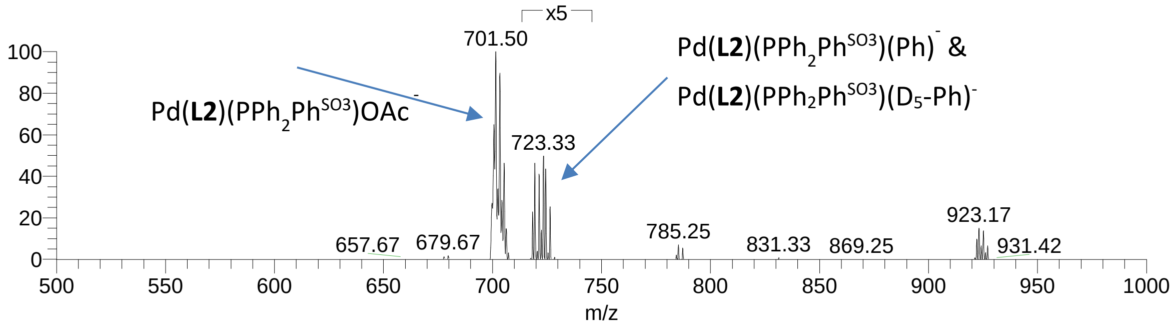

Figure S16. ESI mass spectrum (negative mode) of $[Pd(\mathbf{L2})(PPh_2Ph^{SO3})OAc\,]Na$ in benzene and deuterated benzene (heated for 5 min at 80 °C and filtered, filtrate injected).

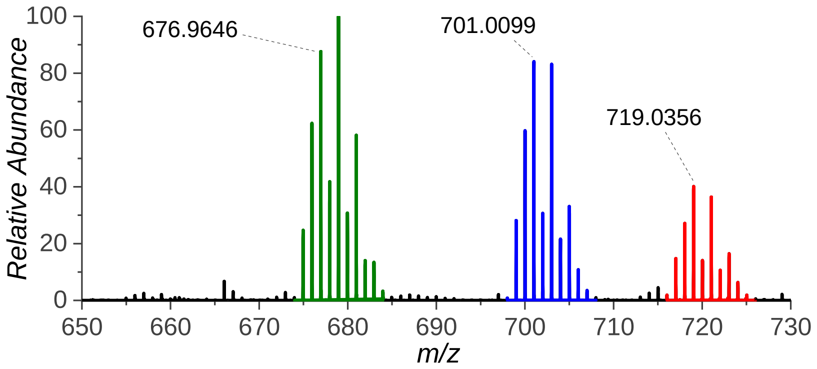

Figure S17. High resolution mass spectrum (negative mode) of [Pd(**L2**)(PPh_2Ph^{SO3})OAc]Na in benzene and DCM (heated for 5min at 60 ℃ and filtered, filtrate injected); [(PPh_2Ph^{SO3-})Pd(**L2**)(X)]$^-$ ions highlighted, where X = Cl (*m/z* 677, green), AcO (*m/z* 701, blue), or Ph (*m/z* 719, red).

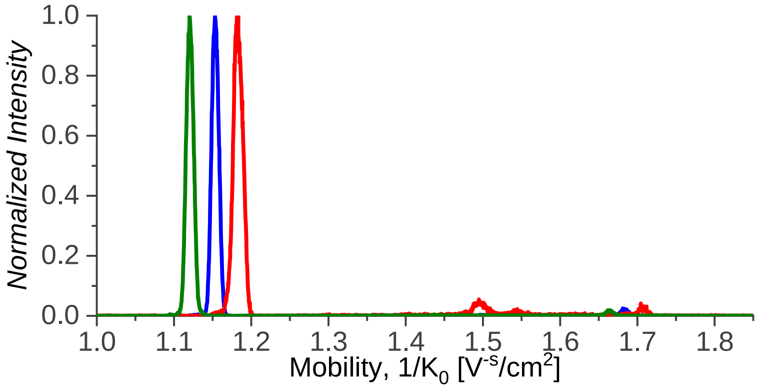

Figure S18. Mobilogram of [(PPh_2Ph^{SO3-})Pd(**L2**)(X)]$^-$, where X = Cl (*m/z* 677, green), AcO (*m/z* 701, blue), or Ph (*m/z* 719, red). Note that the minor peaks at the higher values of mobility originate from fragmentations of larger clusters; i.e., they do not correspond to isomers of the monopalladium complexes.

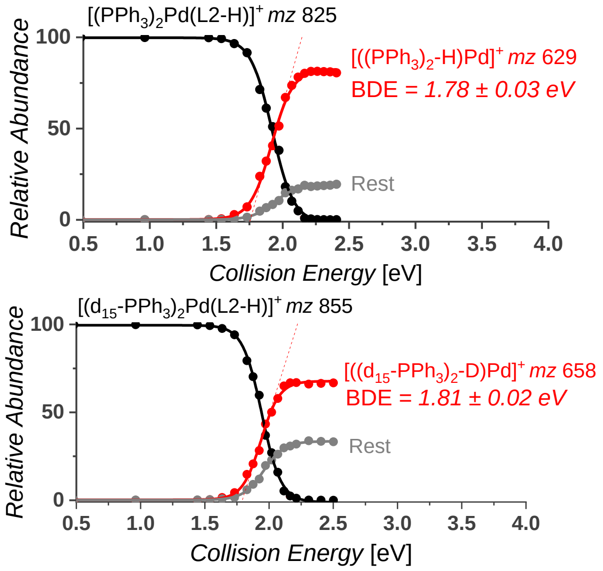

Figure S19. Energy resolved CIDs of $[(PPh_3)_2Pd(\mathbf{L2})]^+$ *m/z*=825 (left) and $[(P(C_6D_5)_3)_2Pd(\mathbf{L2})]^+$ *m/z*=855 (right).

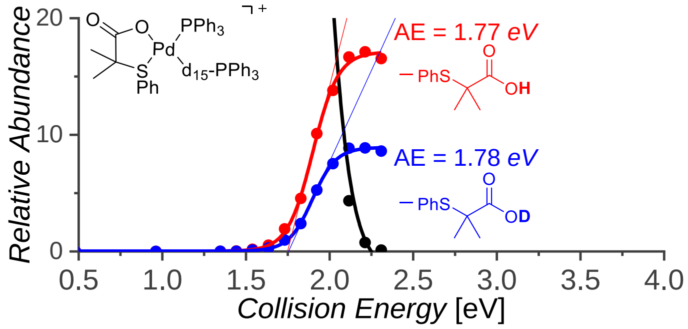

Figure S20. Energy resolved CID of $[(PPh_3)(d_{15}\text{-}PPh_3)Pd(\mathbf{L2})]^+$ (*m/z*=840).

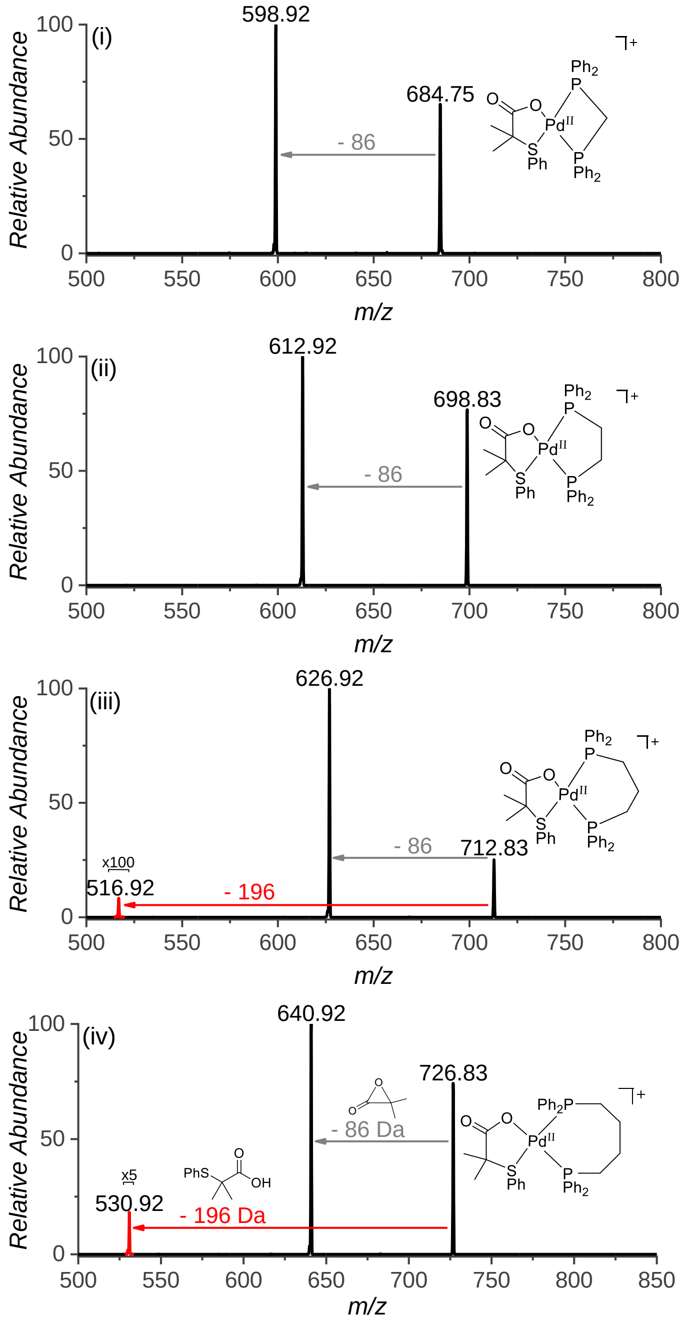

Figure S21. CID mass spectra of (i) [(dppm)Pd(**L2**)]$^+$ (*m/z*=685), (ii) [(dppe)Pd(**L2**)]$^+$ (*m/z*=699), (iii) [(dppp)Pd(**L2**)]$^+$ (*m/z*=713) and (iv) [(dppb)Pd(**L2**)]$^+$ (*m/z*=727).

7. DFT calculations

All structures were optimized using density functional theory (DFT) as implemented in Gaussian 16,[8] with B3LYP[9] as functional, 6-31G(d,p) as basis set for non-metallic atoms, and SDD[10] as basis set for palladium. Final energies were obtained performing single-point calculations on the previously optimized structures at M06[11]/def2tzvpp[12] level of theory, introducing solvation factors with the IEF-PCM[13] method, and acetic acid as solvent. The stationary points were characterized by frequency calculations in order to verify that they have the right number of imaginary frequencies.

Table S39. Energies of the structures included in the main Manuscript

Compound	E (B3LYP)	Correction to G	G (M06)	ΔG (kcal/mol)	Imaginary freq. (cm^{-1})
**$Pd_3(OAc)_6$**	-1754.741193	0.242712	-1754.498481	0	
**I** ($Pd(OAc)_2$)	-584.877443	0.064476	-584.812967	+12.5	
HOAc	-229.064385	0.034877	-229.029508		
Ligand **L2**	-936.787272	0.159941	-936.627331		
**II**	-1292.625462	0.193240	-1292.432222	-0.9	
**III**	-2000.373320	0.320370	-2000.052950	-15.3	
Benzene	-232.143505	0.07314	-232.070365		
**IV**	-817.026177	0.156968	-816.869209	+8.9	
**V**	-1392.258978	0.270498	-1391.988480		
**VI**	-1624.423646	0.365825	-1624.057821	+0.6	
**VII**	-2100.004899	0.398180	-2099.606719		
**VIII**	-2332.177308	0.495660	-2331.681648	-2.9	
**TS-A**	-2332.116435	0.488351	-2331.628084	+33.6	-1415.5
**IX**	-2332.156985	0.496851	-2331.660134	+13.5	
**TS-B**	-2332.110667	0.485327	-2331.625340	+35.3	-582.0
**VIII-OAc**	-2560.785447	0.538731	-2560.246716	-7.5	
**TS-C**	-2560.769467	0.535771	-2560.233696	+0.7	-838.7

Cartesian coordinates of the optimized structures are shown below, as well as their single point energy and correction to Gibbs free energy (in Hartrees).

$Pd_3(OAc)_6$:

Standard orientation:

Center Number	Atomic Number	Atomic Type	Coordinates (Angstroms) X	Y	Z
1	46	0	-0.041846	-1.870081	-0.000251
2	8	0	-2.385559	0.020794	-1.613349
3	6	0	-2.329656	-1.222021	-1.863010
4	8	0	-1.605465	-2.092314	-1.293891
5	6	0	-3.259194	-1.729598	-2.945940
6	1	0	-2.806630	-2.573690	-3.468142
7	1	0	-4.185984	-2.073188	-2.474105
8	1	0	-3.504648	-0.926283	-3.641598
9	8	0	1.510466	-2.160647	1.294178
10	6	0	2.271565	-1.323111	1.863913
11	8	0	2.381506	-0.083685	1.614832
12	6	0	3.178700	-1.868661	2.947344
13	1	0	2.719612	-2.733074	3.428385
14	1	0	4.119203	-2.186301	2.484513
15	1	0	3.404421	-1.089905	3.677208
16	46	0	-1.601387	0.972211	0.004679
17	46	0	1.643544	0.900921	-0.005037
18	8	0	-1.107771	2.100211	1.624504
19	6	0	0.022650	2.618353	1.875229
20	8	0	1.123510	2.391125	1.289510
21	6	0	0.071036	3.602233	3.025861
22	1	0	0.815158	4.374877	2.825614
23	1	0	-0.911675	4.043305	3.194940
24	1	0	0.377346	3.064499	3.929454
25	8	0	-2.626414	-0.225561	1.302239
26	6	0	-2.270188	-1.294439	1.883224
27	8	0	-1.260725	-2.016382	1.621816
28	6	0	-3.139496	-1.744290	3.039201
29	1	0	-2.987604	-2.803955	3.245251
30	1	0	-2.864744	-1.164711	3.926757
31	1	0	-4.188302	-1.538459	2.817477
32	8	0	2.614080	-0.342685	-1.300291
33	6	0	2.210190	-1.393869	-1.882617
34	8	0	1.168464	-2.069161	-1.623209
35	6	0	3.062370	-1.882128	-3.035742
36	1	0	4.119623	-1.763774	-2.790265
37	1	0	2.834443	-2.921804	-3.270931
38	1	0	2.852170	-1.260322	-3.912142
39	8	0	1.202019	2.049776	-1.625545
40	6	0	0.094632	2.614566	-1.877731
41	8	0	-1.015122	2.435710	-1.291923
42	6	0	0.086328	3.600680	-3.027599
43	1	0	-0.327706	3.102645	-3.910550
44	1	0	-0.562177	4.445188	-2.786740
45	1	0	1.097627	3.940887	-3.250885

$Pd(OAc)_2$:

Standard orientation:

Center Number	Atomic Number	Atomic Type	Coordinates (Angstroms) X	Y	Z
1	46	0	0.000017	-0.000075	-0.000104
2	8	0	1.762329	1.088029	0.017933
3	6	0	2.436426	-0.000016	0.017634
4	8	0	1.762594	-1.088157	0.018119
5	6	0	3.931471	0.000253	-0.013596
6	1	0	4.317007	-0.899216	0.469775
7	1	0	4.264866	0.002529	-1.057065
8	1	0	4.316617	0.897978	0.473361
9	8	0	-1.762470	-1.088086	-0.018105
10	6	0	-2.436408	-0.000082	-0.017475
11	8	0	-1.762503	1.088137	-0.017909
12	6	0	-3.931535	0.000182	0.013976
13	1	0	-4.317301	-0.897427	-0.472696
14	1	0	-4.264740	-0.001603	1.057498
15	1	0	-4.316563	0.899764	-0.469614

Ligand **L2**:

Standard orientation:

Center Number	Atomic Number	Atomic Type	Coordinates (Angstroms) X	Y	Z
1	6	0	-1.415433	-0.011049	0.476247
2	6	0	-0.986180	1.219633	1.274472
3	1	0	-1.649352	1.345608	2.135826
4	1	0	0.036440	1.101409	1.638377
5	1	0	-1.048287	2.126153	0.670198
6	6	0	-2.833753	0.205157	-0.058118
7	8	0	-3.510196	1.190594	0.133533
8	8	0	-3.279789	-0.853190	-0.775411
9	6	0	1.303281	-0.079256	-0.501755
10	6	0	1.966434	1.155971	-0.544697
11	6	0	1.997799	-1.217222	-0.064725
12	6	0	3.297725	1.252988	-0.138979
13	1	0	1.435037	2.032324	-0.900600
14	6	0	3.328233	-1.114755	0.343904
15	1	0	1.495370	-2.178832	-0.060844
16	6	0	3.978939	0.119644	0.309221
17	1	0	3.803244	2.213543	-0.176243
18	1	0	3.857794	-2.001005	0.681216
19	1	0	5.015884	0.196902	0.622643
20	16	0	-0.381749	-0.224667	-1.101576
21	6	0	-1.358128	-1.286983	1.332447
22	1	0	-2.001371	-1.178517	2.214109
23	1	0	-1.683637	-2.160465	0.765960

24	1	0	-0.335731	-1.452128	1.683147
25	1	0	-4.171037	-0.614328	-1.081643

PPh_3

Standard orientation:

Center Number	Atomic Number	Atomic Type	Coordinates (Angstroms) X	Y	Z
1	15	0	0.000572	-0.000303	-1.208857
2	6	0	-1.209555	-1.151830	-0.404320
3	6	0	-2.458107	-1.299632	-1.031732
4	6	0	-0.949852	-1.898507	0.754801
5	6	0	-3.427653	-2.151866	-0.504586
6	1	0	-2.668158	-0.745408	-1.943087
7	6	0	-1.916506	-2.760806	1.276320
8	1	0	0.011984	-1.809749	1.249621
9	6	0	-3.157720	-2.887111	0.651317
10	1	0	-4.388562	-2.250148	-1.001658
11	1	0	-1.698130	-3.333759	2.173292
12	1	0	-3.907838	-3.559143	1.058198
13	6	0	1.602702	-0.471584	-0.403476
14	6	0	2.111215	0.117858	0.763874
15	6	0	2.362576	-1.469241	-1.037051
16	6	0	3.340832	-0.287089	1.287121
17	1	0	1.546807	0.898612	1.263579
18	6	0	3.585157	-1.881779	-0.508351
19	1	0	1.993863	-1.921363	-1.954355
20	6	0	4.078659	-1.289156	0.655545
21	1	0	3.721413	0.181503	2.190502
22	1	0	4.156841	-2.657203	-1.010327
23	1	0	5.035418	-1.601948	1.063753
24	6	0	-0.392136	1.622969	-0.403584
25	6	0	0.104972	2.778620	-1.029452
26	6	0	-1.170109	1.770472	0.754710
27	6	0	-0.148957	4.043942	-0.501607
28	1	0	0.691212	2.684208	-1.940092
29	6	0	-1.434241	3.038375	1.276859
30	1	0	-1.574928	0.892653	1.248154
31	6	0	-0.922149	4.176925	0.653417
32	1	0	0.247118	4.925626	-0.997445
33	1	0	-2.040822	3.135050	2.173092
34	1	0	-1.129569	5.162249	1.060797

II

Standard orientation:

Center Number	Atomic Number	Atomic Type	Coordinates (Angstroms) X	Y	Z
1	46	0	1.337631	0.205851	-0.172156
2	8	0	-0.093344	3.979161	0.450914
3	6	0	0.016987	2.781876	0.256866
4	8	0	1.169741	2.161127	0.179339
5	6	0	-1.281349	1.924181	0.178601
6	8	0	3.353910	-0.253318	0.330600
7	6	0	3.136783	-1.462800	0.001756
8	8	0	1.953872	-1.767086	-0.401834
9	6	0	4.204625	-2.510989	0.089908
10	1	0	4.233562	-3.087915	-0.838046
11	1	0	5.173381	-2.048071	0.280834
12	1	0	3.965411	-3.202856	0.904053
13	16	0	-0.834236	0.422330	-0.897648
14	6	0	-1.662723	1.510790	1.605754
15	1	0	-1.839480	2.426267	2.178584
16	1	0	-2.576421	0.910892	1.618541
17	1	0	-0.862992	0.950186	2.096519
18	6	0	-2.408226	2.694537	-0.507999
19	1	0	-2.560536	3.629626	0.036905
20	1	0	-2.152550	2.947334	-1.540022
21	1	0	-3.339846	2.119695	-0.501788
22	6	0	-1.856936	-0.936950	-0.314735
23	6	0	-3.114049	-1.097861	-0.912948
24	6	0	-1.415933	-1.833061	0.665812
25	6	0	-3.940201	-2.146092	-0.506974
26	1	0	-3.437649	-0.414854	-1.692039
27	6	0	-2.247201	-2.883590	1.056068
28	1	0	-0.430085	-1.717396	1.102432
29	6	0	-3.508201	-3.038084	0.476674
30	1	0	-4.915892	-2.269470	-0.967003
31	1	0	-1.904889	-3.582932	1.812784
32	1	0	-4.150169	-3.857755	0.784960

III

Standard orientation:

Center Number	Atomic Number	Atomic Type	Coordinates (Angstroms) X	Y	Z
1	46	0	-0.019451	-1.161272	-0.033966
2	6	0	2.885425	-1.104571	-1.181449
3	6	0	4.265704	-0.471514	-1.327399
4	1	0	4.510583	0.175882	-0.482862
5	1	0	4.999869	-1.280467	-1.343211

6	1	0	4.341111	0.105099	-2.254738
7	6	0	2.794930	-2.006470	0.083937
8	8	0	1.627276	-2.124823	0.663330
9	8	0	3.802567	-2.588784	0.450761
10	16	0	1.549400	0.232750	-1.067516
11	6	0	2.520497	-1.942613	-2.420220
12	1	0	1.515870	-2.368828	-2.345769
13	1	0	2.588694	-1.346626	-3.335565
14	1	0	3.233208	-2.769874	-2.488679
15	6	0	2.155705	1.363574	0.202743
16	6	0	2.487300	2.659508	-0.210854
17	6	0	2.278993	0.990572	1.548599
18	6	0	2.945667	3.587195	0.726419
19	1	0	2.387116	2.937223	-1.255419
20	6	0	2.744901	1.925919	2.473719
21	1	0	2.021213	-0.014815	1.863760
22	6	0	3.075510	3.221244	2.067191
23	1	0	3.203693	4.592014	0.405608
24	1	0	2.847510	1.637488	3.515460
25	1	0	3.434713	3.943057	2.794656
26	8	0	-1.207104	-2.546256	0.844821
27	6	0	-2.505332	-2.434595	0.932934
28	8	0	-3.239329	-3.298663	1.383307
29	6	0	-3.160894	-1.075773	0.529012
30	6	0	-3.210147	-0.193701	1.782720
31	1	0	-3.675200	0.774362	1.578679
32	1	0	-3.814652	-0.719418	2.528106
33	1	0	-2.216166	-0.030603	2.207215
34	6	0	-4.552673	-1.287749	-0.065415
35	1	0	-5.037539	-0.331430	-0.287372
36	1	0	-4.512614	-1.886832	-0.978519
37	1	0	-5.152842	-1.834578	0.665984
38	16	0	-2.047165	-0.337364	-0.821877
39	6	0	-2.219547	1.448032	-0.690552
40	6	0	-3.137661	2.067818	-1.549052
41	6	0	-1.461712	2.213000	0.204444
42	6	0	-3.304296	3.452379	-1.499492
43	1	0	-3.709887	1.470514	-2.251832
44	6	0	-1.631505	3.597482	0.241895
45	1	0	-0.746185	1.730631	0.860925
46	6	0	-2.552462	4.217244	-0.605547
47	1	0	-4.016834	3.931395	-2.164125
48	1	0	-1.039863	4.189400	0.933581
49	1	0	-2.680524	5.295047	-0.572691

IV

Standard orientation:

Center Number	Atomic Number	Atomic Type	Coordinates (Angstroms) X	Y	Z
1	46	0	-0.124445	0.289172	-0.213089
2	8	0	-0.030835	2.418359	-0.208837
3	6	0	1.211873	2.341680	0.064811
4	8	0	1.727035	1.170367	0.163706
5	6	0	2.037193	3.571329	0.293895
6	1	0	3.096504	3.349450	0.154733
7	1	0	1.717153	4.366749	-0.382475
8	1	0	1.880932	3.917382	1.321298
9	6	0	0.120804	-1.885104	0.589488
10	6	0	0.309088	-1.912652	-0.818145
11	6	0	1.252108	-1.934566	1.442736
12	6	0	1.622416	-1.997448	-1.348232
13	1	0	-0.547900	-2.024902	-1.473805
14	6	0	2.523445	-2.005225	0.904646
15	1	0	1.104293	-1.925644	2.517804
16	6	0	2.709589	-2.035958	-0.495391
17	1	0	1.760984	-2.042204	-2.423895
18	1	0	-0.873943	-1.947820	1.017702
19	8	0	-2.562548	-0.314263	1.444171
20	6	0	-2.873509	-0.215582	0.260604
21	8	0	-2.017257	-0.082630	-0.734252
22	6	0	-4.318995	-0.221167	-0.212944
23	1	0	-4.964445	-0.587062	0.586552
24	1	0	-4.612985	0.801141	-0.472266
25	1	0	-4.435621	-0.834491	-1.109840
26	1	0	3.715318	-2.097626	-0.900147
27	1	0	3.388931	-2.043397	1.559327

V

Standard orientation:

Center Number	Atomic Number	Atomic Type	Coordinates (Angstroms) X	Y	Z
1	46	0	-1.459845	-0.191579	-0.293091
2	8	0	-3.570700	-0.303041	-0.636713
3	6	0	-3.369606	0.076174	-1.835306
4	8	0	-2.146268	0.313453	-2.177464
5	15	0	0.764895	0.026337	0.047740
6	6	0	-0.046727	-0.185209	1.655231
7	6	0	-0.868242	-1.351298	1.753756
8	6	0	-0.181439	0.846925	2.616258
9	6	0	-1.794245	-1.461347	2.813399
10	1	0	-0.671318	-2.222310	1.132704
11	6	0	-1.081777	0.699438	3.657740
12	1	0	0.429179	1.740244	2.536476
13	6	0	-1.898822	-0.446323	3.750419
14	1	0	-2.402856	-2.355714	2.895467
15	1	0	-1.162896	1.477437	4.410459

16	1	0	-2.604126	-0.535990	4.570328
17	6	0	1.864249	-1.351054	-0.344959
18	6	0	2.015237	-1.727824	-1.691699
19	6	0	2.565604	-2.032596	0.665385
20	6	0	2.870483	-2.777600	-2.020690
21	1	0	1.467130	-1.209908	-2.473614
22	6	0	3.416519	-3.082794	0.322174
23	1	0	2.453069	-1.745529	1.706331
24	6	0	3.568263	-3.453922	-1.016202
25	1	0	2.987076	-3.071155	-3.058876
26	1	0	3.960749	-3.609363	1.099664
27	1	0	4.230408	-4.273607	-1.276917
28	6	0	1.584291	1.620216	-0.139987
29	6	0	0.802908	2.778968	-0.307439
30	6	0	2.987998	1.707184	-0.132851
31	6	0	1.426968	4.017071	-0.441607
32	1	0	-0.280370	2.708964	-0.345370
33	6	0	3.599335	2.951740	-0.269344
34	1	0	3.594525	0.814077	-0.023541
35	6	0	2.821938	4.103031	-0.421583
36	1	0	0.826482	4.911272	-0.573790
37	1	0	4.682238	3.021980	-0.260601
38	1	0	3.304612	5.068859	-0.533864
39	6	0	-4.475745	0.219773	-2.827897
40	1	0	-4.234613	0.994454	-3.558153
41	1	0	-4.595726	-0.729591	-3.361714
42	1	0	-5.411508	0.446850	-2.314265

VI

Standard orientation:

Center Number	Atomic Number	Atomic Type	Coordinates (Angstroms) X	Y	Z
1	46	0	-1.110453	-1.178450	-0.204084
2	6	0	2.471117	-2.946152	-0.897985
3	6	0	2.412396	-3.039826	0.508281
4	6	0	1.311764	-2.967663	-1.655526
5	6	0	1.193879	-3.163845	1.153661
6	6	0	0.058881	-3.109282	-1.017284
7	1	0	1.357905	-2.926748	-2.739163
8	6	0	-0.000456	-3.210881	0.398432
9	1	0	3.436897	-2.873393	-1.386840
10	1	0	-0.821842	-3.330371	-1.614602
11	1	0	-0.926198	-3.514396	0.880689
12	1	0	1.149529	-3.274576	2.232563
13	1	0	3.332676	-3.027457	1.083071
14	8	0	-2.865492	-0.034629	-0.198445
15	6	0	-3.592893	-1.097162	-0.276103
16	8	0	-2.986505	-2.217246	-0.344839
17	15	0	0.313505	0.624468	0.042215
18	6	0	2.107392	0.414513	0.320079
19	6	0	2.965054	0.253266	-0.784007
20	6	0	2.650706	0.427287	1.616001
21	6	0	4.340950	0.132351	-0.592265
22	1	0	2.564072	0.248692	-1.792164
23	6	0	4.026867	0.294454	1.800231
24	1	0	2.008999	0.569050	2.478397
25	6	0	4.873908	0.150971	0.698807
26	1	0	4.996298	0.030070	-1.451866
27	1	0	4.437998	0.321633	2.804583
28	1	0	5.946045	0.063047	0.845503
29	6	0	0.171476	1.735923	-1.402967
30	6	0	1.082992	2.795543	-1.572603
31	6	0	-0.870048	1.567619	-2.330109
32	6	0	0.947248	3.668100	-2.651257
33	1	0	1.897030	2.938726	-0.869741
34	6	0	-0.993416	2.441253	-3.411033
35	6	0	-0.087708	3.490437	-3.572772
36	1	0	1.652895	4.483965	-2.772977
37	1	0	-1.799110	2.300885	-4.124720
38	1	0	-0.186645	4.168907	-4.414451
39	1	0	-1.589990	0.767087	-2.203820
40	6	0	-0.369025	1.484503	1.506360
41	6	0	-0.642425	0.741678	2.669716
42	6	0	-0.642892	2.860326	1.484813
43	6	0	-1.157244	1.373404	3.800368
44	1	0	-0.460783	-0.329464	2.691309
45	6	0	-1.162713	3.484453	2.619590
46	1	0	-0.457543	3.443581	0.589909
47	6	0	-1.417588	2.745955	3.776055
48	1	0	-1.362762	0.794083	4.695132
49	1	0	-1.371559	4.549388	2.595913
50	1	0	-1.824642	3.236260	4.654868
51	6	0	-5.083111	-1.001072	-0.253827
52	1	0	-5.520663	-1.842353	-0.794423
53	1	0	-5.424165	-1.045398	0.786600
54	1	0	-5.410868	-0.051448	-0.680797

VII

Standard orientation:

Center Number	Atomic Number	Atomic Type	Coordinates (Angstroms) X	Y	Z
1	46	0	0.337379	-0.319336	0.161497
2	8	0	2.088695	-2.147076	-3.031045
3	6	0	1.834751	-1.760181	-1.908946
4	8	0	0.729729	-1.072446	-1.652052
5	6	0	2.770115	-2.144885	-0.735000

6	6	0	2.272333	-3.475493	-0.135500
7	1	0	2.932527	-3.811478	0.668615
8	1	0	1.252892	-3.397177	0.255142
9	1	0	2.275219	-4.230251	-0.927130
10	6	0	4.222219	-2.253759	-1.194678
11	1	0	4.602964	-1.306092	-1.580074
12	1	0	4.866526	-2.592341	-0.378388
13	1	0	4.263047	-2.981882	-2.008334
14	16	0	2.604708	-0.887443	0.665044
15	6	0	3.560925	0.549731	0.147778
16	6	0	3.235635	1.303708	-0.989274
17	6	0	4.654649	0.899706	0.949249
18	6	0	4.021741	2.407307	-1.321314
19	1	0	2.390614	1.028872	-1.611596
20	6	0	5.426248	2.012815	0.610170
21	1	0	4.903106	0.304950	1.822624
22	6	0	5.112195	2.764273	-0.523239
23	1	0	3.779789	2.988395	-2.205766
24	1	0	6.275692	2.284840	1.228727
25	1	0	5.717413	3.625783	-0.787333
26	15	0	-1.889032	0.227706	0.002872
27	6	0	-1.417956	0.340797	1.757889
28	6	0	-0.311967	1.195905	2.041075
29	6	0	-1.869213	-0.560940	2.749562
30	6	0	0.299104	1.147170	3.311232
31	1	0	-0.040729	1.994616	1.354407
32	6	0	-1.259716	-0.578079	3.994789
33	1	0	-2.700171	-1.224795	2.534358
34	6	0	-0.169583	0.267584	4.275331
35	1	0	1.120200	1.821784	3.531581
36	1	0	-1.628859	-1.250772	4.762835
37	1	0	0.294400	0.238553	5.256033
38	6	0	-2.383111	1.844522	-0.643389
39	6	0	-2.143666	2.114301	-2.002665
40	6	0	-3.003991	2.807965	0.170176
41	6	0	-2.533872	3.339167	-2.541384
42	1	0	-1.655107	1.374773	-2.631063
43	6	0	-3.385551	4.031575	-0.380158
44	1	0	-3.190813	2.605759	1.220460
45	6	0	-3.152114	4.295916	-1.731948
46	1	0	-2.350527	3.547875	-3.590523
47	1	0	-3.866778	4.776103	0.246210
48	1	0	-3.451293	5.249893	-2.154874
49	6	0	-3.172861	-1.011327	-0.281790
50	6	0	-2.797607	-2.355682	-0.451023
51	6	0	-4.527865	-0.641720	-0.348915
52	6	0	-3.777322	-3.324883	-0.660346
53	1	0	-1.748113	-2.633394	-0.438496
54	6	0	-5.497906	-1.619568	-0.558949
55	1	0	-4.820691	0.398279	-0.244773
56	6	0	-5.124110	-2.957707	-0.712265
57	1	0	-3.488477	-4.362000	-0.797376
58	1	0	-6.544524	-1.336586	-0.608566
59	1	0	-5.883994	-3.713896	-0.883343

VIII

Standard orientation:

Center Number	Atomic Number	Atomic Type	Coordinates (Angstroms) X	Y	Z
1	46	0	-0.368695	-0.937323	-0.036191
2	6	0	0.590586	-2.429322	-1.780030
3	6	0	0.922366	-2.991072	-0.521246
4	6	0	-0.438507	-3.007736	-2.551879
5	6	0	0.222696	-4.123809	-0.053372
6	6	0	-1.119990	-4.115398	-2.073223
7	1	0	-0.675358	-2.594921	-3.527351
8	6	0	-0.788792	-4.674488	-0.823555
9	1	0	1.231102	-1.672932	-2.220702
10	1	0	-1.909693	-4.565114	-2.666480
11	1	0	-1.327077	-5.547806	-0.469512
12	1	0	0.497215	-4.570423	0.897050
13	1	0	1.819926	-2.669459	-0.005642
14	8	0	-2.096954	-2.018825	-0.104119
15	6	0	-3.109969	-1.826478	0.711995
16	8	0	-4.112152	-2.516991	0.705372
17	6	0	-2.976616	-0.697791	1.763914
18	6	0	-4.320964	-0.046492	2.075209
19	1	0	-4.227467	0.699069	2.870357
20	1	0	-4.766849	0.420728	1.195598
21	1	0	-4.997430	-0.839262	2.404798
22	6	0	-2.335165	-1.279504	3.036182
23	1	0	-1.370806	-1.754722	2.832641
24	1	0	-2.204595	-0.510108	3.802709
25	1	0	-3.006996	-2.048270	3.428753
26	16	0	-1.756095	0.595831	1.102329
27	6	0	-2.692068	1.483817	-0.155961
28	6	0	-2.953082	2.833792	0.106963
29	6	0	-3.158476	0.874406	-1.328784
30	6	0	-3.693851	3.579125	-0.811908
31	1	0	-2.584341	3.294672	1.017580
32	6	0	-3.900519	1.631580	-2.235236
33	1	0	-2.954228	-0.172581	-1.523489
34	6	0	-4.167873	2.979856	-1.979901
35	1	0	-3.902716	4.624859	-0.609454
36	1	0	-4.273559	1.164029	-3.141266
37	1	0	-4.748354	3.560516	-2.690116
38	15	0	1.545558	0.444802	0.086711
39	6	0	2.978172	-0.063150	-0.954745
40	6	0	3.169442	0.451821	-2.247526

41	6	0	3.875273	-1.031133	-0.466416
42	6	0	4.232408	0.003546	-3.033473
43	1	0	2.507639	1.216559	-2.638229
44	6	0	4.935215	-1.474354	-1.256911
45	1	0	3.767354	-1.420011	0.541589
46	6	0	5.113864	-0.961215	-2.543300
47	1	0	4.374801	0.418101	-4.026673
48	1	0	5.624493	-2.215186	-0.863732
49	1	0	5.940787	-1.305336	-3.156535
50	6	0	1.109013	2.143254	-0.456722
51	6	0	0.377433	2.294863	-1.648732
52	6	0	1.487998	3.285965	0.263987
53	6	0	0.065235	3.566309	-2.128306
54	1	0	0.041351	1.420516	-2.199264
55	6	0	1.161160	4.555961	-0.215061
56	1	0	2.034484	3.192504	1.195376
57	6	0	0.459368	4.699025	-1.412788
58	1	0	-0.495567	3.669917	-3.051802
59	1	0	1.460245	5.433504	0.349862
60	1	0	0.213729	5.689187	-1.784114
61	6	0	2.246471	0.553347	1.775689
62	6	0	1.557816	0.007738	2.869294
63	6	0	3.489306	1.178528	1.991593
64	6	0	2.091341	0.095669	4.156621
65	1	0	0.609940	-0.495809	2.715715
66	6	0	4.014739	1.269193	3.279325
67	1	0	4.050378	1.584932	1.155962
68	6	0	3.316471	0.729597	4.363247
69	1	0	1.551290	-0.334839	4.994156
70	1	0	4.972706	1.755406	3.435382
71	1	0	3.731558	0.797640	5.364029

TS-A

Standard orientation:

Center Number	Atomic Number	Atomic Type	Coordinates (Angstroms) X	Y	Z
1	46	0	-0.301544	-0.834892	-0.111735
2	6	0	-2.937351	-1.791543	1.417580
3	6	0	-4.387936	-2.067004	0.999456
4	1	0	-4.514231	-2.079655	-0.084428
5	1	0	-4.672015	-3.048421	1.391189
6	1	0	-5.063569	-1.318876	1.422529
7	6	0	3.286092	-3.675405	-1.917643
8	6	0	3.106354	-3.597841	-0.531318
9	6	0	2.366717	-3.083847	-2.791435
10	6	0	2.002795	-2.922446	-0.015517
11	6	0	1.265610	-2.405242	-2.276724
12	1	0	2.509444	-3.159318	-3.865036
13	6	0	1.049364	-2.325700	-0.878166
14	1	0	4.143743	-4.207811	-2.318971
15	1	0	0.546379	-1.959684	-2.958549
16	1	0	-0.220893	-2.811927	-0.614656
17	1	0	1.858111	-2.880405	1.060204
18	1	0	3.819002	-4.070743	0.137475
19	6	0	-2.020013	-2.894934	0.810771
20	8	0	-1.456857	-2.588971	-0.384885
21	8	0	-1.866926	-3.954377	1.365860
22	16	0	-2.374554	-0.038200	0.898533
23	6	0	-2.789818	-1.806868	2.947916
24	1	0	-1.780927	-1.523449	3.264366
25	1	0	-3.503798	-1.121336	3.409699
26	1	0	-2.977102	-2.821673	3.305261
27	6	0	-3.430091	0.454829	-0.476791
28	6	0	-4.337036	1.492405	-0.226625
29	6	0	-3.351972	-0.132185	-1.747858
30	6	0	-5.168057	1.943914	-1.254173
31	1	0	-4.389572	1.942449	0.759461
32	6	0	-4.190780	0.325355	-2.764460
33	1	0	-2.658643	-0.944884	-1.932802
34	6	0	-5.095849	1.362275	-2.520785
35	1	0	-5.873271	2.746077	-1.060179
36	1	0	-4.137820	-0.131579	-3.747880
37	1	0	-5.745099	1.712980	-3.316916
38	15	0	1.179708	0.903110	0.194264
39	6	0	2.233793	0.608690	1.662654
40	6	0	1.745670	-0.181761	2.716122
41	6	0	3.503740	1.198150	1.776410
42	6	0	2.512430	-0.378338	3.864483
43	1	0	0.766731	-0.646081	2.635272
44	6	0	4.267051	0.997702	2.927287
45	1	0	3.897835	1.808598	0.970674
46	6	0	3.774376	0.210537	3.970253
47	1	0	2.127164	-0.992701	4.672449
48	1	0	5.247880	1.456160	3.006819
49	1	0	4.373150	0.054548	4.862289
50	6	0	2.333807	1.238312	-1.194037
51	6	0	2.255096	2.418068	-1.950450
52	6	0	3.326618	0.290569	-1.500377
53	6	0	3.151776	2.641512	-2.997400
54	1	0	1.508563	3.169555	-1.721789
55	6	0	4.222909	0.524943	-2.540676
56	1	0	3.408201	-0.624099	-0.922804
57	6	0	4.135354	1.698565	-3.294336
58	1	0	3.082988	3.559218	-3.573229
59	1	0	4.989867	-0.210440	-2.763256
60	1	0	4.833876	1.877370	-4.105982
61	6	0	0.286612	2.475565	0.499765
62	6	0	-0.735088	2.862174	-0.385911

63	6	0	0.605409	3.304840	1.585647
64	6	0	-1.411906	4.066452	-0.196012
65	1	0	-1.001565	2.226864	-1.225946
66	6	0	-0.082496	4.504901	1.775042
67	1	0	1.387428	3.020911	2.281020
68	6	0	-1.086963	4.888845	0.885762
69	1	0	-2.195160	4.357284	-0.889036
70	1	0	0.171243	5.139894	2.618207
71	1	0	-1.616732	5.824611	1.035348

IX

Standard orientation:

Center Number	Atomic Number	Atomic Type	Coordinates (Angstroms) X	Y	Z
1	46	0	0.064919	0.477070	-0.609832
2	6	0	-3.117377	-1.913590	-1.195225
3	6	0	-4.075231	-1.379661	-2.261715
4	1	0	-4.448758	-0.386264	-2.011015
5	1	0	-3.558216	-1.314858	-3.225558
6	1	0	-4.922397	-2.061530	-2.369689
7	6	0	-1.758838	3.526853	-1.186259
8	6	0	-0.925714	3.322575	-0.100747
9	6	0	-1.324463	3.214784	-2.494493
10	6	0	0.373555	2.788695	-0.297430
11	6	0	-0.059315	2.698130	-2.711851
12	1	0	-1.987850	3.395142	-3.334227
13	6	0	0.808755	2.475865	-1.613452
14	1	0	-2.750820	3.940735	-1.037691
15	1	0	0.286886	2.483456	-3.717313
16	1	0	1.851070	2.246437	-1.800971
17	1	0	1.082634	2.798560	0.522699
18	1	0	-1.247255	3.587780	0.900825
19	6	0	-1.943008	-0.974314	-0.994617
20	8	0	-0.826010	-1.412560	-0.516338
21	8	0	-2.014311	0.261161	-1.281261
22	16	0	-3.959614	-2.092880	0.488419
23	6	0	-2.631178	-3.332822	-1.528788
24	1	0	-1.962146	-3.723604	-0.759874
25	1	0	-3.488025	-4.003762	-1.628763
26	1	0	-2.091939	-3.322517	-2.481388
27	6	0	-4.663868	-0.482806	0.848850
28	6	0	-5.940393	-0.141800	0.376145
29	6	0	-3.977439	0.405280	1.690711
30	6	0	-6.509623	1.085090	0.721216
31	1	0	-6.485222	-0.839391	-0.251224
32	6	0	-4.554916	1.627867	2.038984
33	1	0	-3.001843	0.130333	2.079271
34	6	0	-5.818089	1.971178	1.550925
35	1	0	-7.496998	1.343030	0.350100
36	1	0	-4.022298	2.307436	2.697998
37	1	0	-6.267203	2.920987	1.825572
38	15	0	2.093678	-0.185057	0.246130
39	6	0	1.730680	-1.235282	1.696618
40	6	0	0.705872	-0.860832	2.583758
41	6	0	2.468092	-2.404707	1.940189
42	6	0	0.439293	-1.637902	3.710277
43	1	0	0.109990	0.026556	2.390449
44	6	0	2.191124	-3.178750	3.067460
45	1	0	3.252011	-2.713382	1.257463
46	6	0	1.180866	-2.796931	3.952345
47	1	0	-0.352824	-1.344147	4.391619
48	1	0	2.764463	-4.082077	3.250181
49	1	0	0.966323	-3.404944	4.825681
50	6	0	3.006219	-1.190388	-0.975401
51	6	0	4.402306	-1.337962	-0.889505
52	6	0	2.297804	-1.866640	-1.983927
53	6	0	5.075008	-2.148672	-1.803919
54	1	0	4.964133	-0.824765	-0.116714
55	6	0	2.980285	-2.671879	-2.895720
56	1	0	1.219072	-1.773103	-2.052486
57	6	0	4.366978	-2.812421	-2.808315
58	1	0	6.152408	-2.257843	-1.730644
59	1	0	2.427065	-3.188009	-3.673994
60	1	0	4.895054	-3.438387	-3.520889
61	6	0	3.221328	1.146231	0.791050
62	6	0	3.996835	1.840230	-0.157033
63	6	0	3.279020	1.529318	2.141949
64	6	0	4.816217	2.894450	0.244145
65	1	0	3.985727	1.544758	-1.201586
66	6	0	4.100079	2.586976	2.534064
67	1	0	2.698201	0.999110	2.888444
68	6	0	4.866840	3.271148	1.588652
69	1	0	5.417241	3.416901	-0.493190
70	1	0	4.144154	2.869590	3.581070
71	1	0	5.506064	4.091831	1.898617

TS-B

Standard orientation:

Center Number	Atomic Number	Atomic Type	Coordinates (Angstroms) X	Y	Z
1	46	0	0.484085	-0.192110	-0.008590
2	6	0	1.012854	4.448160	-0.524694
3	6	0	1.596931	3.653411	-1.521605
4	6	0	0.030117	3.924595	0.323285
5	6	0	1.208920	2.325999	-1.661452

6	6	0	-0.373050	2.600439	0.175815
7	1	0	-0.430804	4.555393	1.077004
8	6	0	0.202764	1.770560	-0.825461
9	1	0	1.317022	5.485704	-0.419180
10	1	0	-1.171984	2.207384	0.798196
11	1	0	-0.667983	1.098226	-1.429815
12	1	0	1.651833	1.716920	-2.443556
13	1	0	2.344126	4.077611	-2.185360
14	8	0	-1.864169	0.507178	-1.916876
15	6	0	-2.276360	-0.244003	-0.991786
16	8	0	-1.546300	-0.649053	-0.019039
17	6	0	-3.754425	-0.667713	-1.004746
18	15	0	2.736064	-0.330366	0.203365
19	6	0	3.600686	0.469665	1.582501
20	6	0	3.487272	1.861214	1.741690
21	6	0	4.384425	-0.279535	2.477356
22	6	0	4.161254	2.495072	2.784868
23	1	0	2.875664	2.442873	1.058706
24	6	0	5.050998	0.364733	3.519241
25	1	0	4.473111	-1.355614	2.363395
26	6	0	4.940314	1.748987	3.672409
27	1	0	4.072813	3.569753	2.908691
28	1	0	5.654994	-0.214275	4.210709
29	1	0	5.459181	2.245722	4.486355
30	6	0	2.161641	-1.988748	0.703367
31	6	0	2.387309	-3.158020	-0.054915
32	6	0	1.235133	-2.024675	1.781028
33	6	0	1.723135	-4.332704	0.273321
34	1	0	3.087368	-3.139400	-0.883455
35	6	0	0.558281	-3.215167	2.082363
36	1	0	1.113260	-1.162798	2.432713
37	6	0	0.801999	-4.361036	1.332896
38	1	0	1.918293	-5.236287	-0.295837
39	1	0	-0.142355	-3.236599	2.910483
40	1	0	0.285378	-5.285167	1.572107
41	6	0	3.826413	-0.436665	-1.242091
42	6	0	3.269853	-0.770482	-2.491105
43	6	0	5.203444	-0.183030	-1.136027
44	6	0	4.089291	-0.868309	-3.614040
45	1	0	2.201935	-0.951057	-2.583260
46	6	0	6.014297	-0.278484	-2.267080
47	1	0	5.639513	0.087211	-0.180083
48	6	0	5.460718	-0.621775	-3.502201
49	1	0	3.657554	-1.128650	-4.575306
50	1	0	7.078643	-0.083555	-2.181979
51	1	0	6.096143	-0.692300	-4.379531
52	6	0	-4.170847	-1.100440	-2.416998
53	1	0	-5.244047	-1.307893	-2.436399
54	1	0	-3.944735	-0.324983	-3.149136
55	1	0	-3.645590	-2.020053	-2.703727
56	6	0	-4.051480	-1.753361	0.028132
57	1	0	-3.775080	-1.443207	1.036711
58	1	0	-5.114115	-2.004156	0.016373
59	1	0	-3.485184	-2.657285	-0.220375
60	16	0	-4.577226	0.991469	-0.559809
61	6	0	-6.138661	0.507764	0.173724
62	6	0	-6.276604	0.446990	1.568137
63	6	0	-7.253512	0.266272	-0.643888
64	6	0	-7.511786	0.133051	2.136067
65	1	0	-5.417768	0.651823	2.199203
66	6	0	-8.484665	-0.054300	-0.070450
67	1	0	-7.155177	0.345960	-1.721655
68	6	0	-8.614811	-0.122283	1.318374
69	1	0	-7.613314	0.090646	3.216517
70	1	0	-9.343722	-0.240086	-0.708231
71	1	0	-9.575593	-0.364874	1.762454

VIII-OAc

Standard orientation:

Center Number	Atomic Number	Atomic Type	Coordinates (Angstroms) X	Y	Z
1	46	0	-0.218978	-0.766091	-0.438515
2	6	0	0.802605	-0.951972	-2.716147
3	6	0	1.469020	-1.862718	-1.862238
4	6	0	-0.115433	-1.428552	-3.680171
5	6	0	1.209054	-3.245222	-1.995792
6	6	0	-0.344341	-2.789032	-3.799392
7	1	0	-0.607951	-0.726151	-4.345808
8	6	0	0.325593	-3.701050	-2.958098
9	1	0	1.111800	0.086202	-2.753879
10	1	0	-1.034650	-3.159864	-4.551341
11	1	0	0.143131	-4.765508	-3.070582
12	1	0	1.722896	-3.938499	-1.339220
13	8	0	-1.456064	-2.386742	-0.753044
14	6	0	-2.245641	-2.866426	0.168616
15	8	0	-2.920414	-3.879684	0.012012
16	6	0	-2.306584	-2.143185	1.536071
17	6	0	-3.710038	-2.193449	2.138172
18	1	0	-3.736086	-1.694989	3.111676
19	1	0	-4.461059	-1.743438	1.486369
20	1	0	-3.970398	-3.246021	2.273379
21	6	0	-1.275618	-2.764457	2.495973
22	1	0	-0.248369	-2.714849	2.123220
23	1	0	-1.331616	-2.281208	3.476560
24	1	0	-1.541646	-3.818955	2.622235
25	16	0	-1.775588	-0.339477	1.288923
26	6	0	-3.214422	0.428675	0.518467
27	6	0	-3.891578	1.385474	1.283853
28	6	0	-3.667892	0.085549	-0.762383

29	6	0	-5.033892	1.998403	0.764745
30	1	0	-3.532582	1.644687	2.274428
31	6	0	-4.813874	0.700897	-1.266150
32	1	0	-3.136965	-0.653769	-1.351025
33	6	0	-5.496378	1.655782	-0.506699
34	1	0	-5.560362	2.739488	1.358023
35	1	0	-5.171820	0.433240	-2.255626
36	1	0	-6.386671	2.131250	-0.906727
37	15	0	0.974853	1.217717	0.008973
38	6	0	2.365985	1.662586	-1.112583
39	6	0	2.303110	2.739252	-2.011787
40	6	0	3.539117	0.888415	-1.032574
41	6	0	3.399601	3.036841	-2.823817
42	1	0	1.413640	3.355228	-2.075683
43	6	0	4.626547	1.196170	-1.850787
44	1	0	3.601797	0.044426	-0.346590
45	6	0	4.560874	2.266398	-2.746656
46	1	0	3.343890	3.875947	-3.510899
47	1	0	5.528108	0.594391	-1.785261
48	1	0	5.411729	2.500567	-3.379659
49	6	0	-0.248990	2.584680	-0.140046
50	6	0	-0.971409	2.697415	-1.343027
51	6	0	-0.514154	3.490810	0.897615
52	6	0	-1.904032	3.718731	-1.516836
53	1	0	-0.807952	1.986182	-2.147178
54	6	0	-1.458493	4.505539	0.722624
55	1	0	0.010562	3.412571	1.842187
56	6	0	-2.147429	4.627915	-0.484078
57	1	0	-2.445899	3.797327	-2.454261
58	1	0	-1.650956	5.200700	1.534214
59	1	0	-2.876734	5.421042	-0.617944
60	6	0	1.727214	1.327572	1.676648
61	6	0	1.706282	0.232806	2.551302
62	6	0	2.380502	2.514034	2.061716
63	6	0	2.295112	0.344549	3.814738
64	1	0	1.311441	-0.728649	2.233884
65	6	0	2.959327	2.616760	3.324824
66	1	0	2.443846	3.354374	1.377143
67	6	0	2.908688	1.533429	4.207745
68	1	0	2.285761	-0.512236	4.481780
69	1	0	3.455978	3.537565	3.615410
70	1	0	3.363927	1.613559	5.190573
71	8	0	3.591262	-1.921952	0.455590
72	6	0	3.058640	-2.708135	1.289384
73	8	0	1.832539	-2.780261	1.592481
74	6	0	4.017741	-3.653033	2.042825
75	1	0	4.810315	-4.010588	1.379074
76	1	0	4.501431	-3.098501	2.856533
77	1	0	3.486149	-4.502511	2.479150
78	1	0	2.267463	-1.566545	-1.182475

TS-C

Standard orientation:

Center Number	Atomic Number	Atomic Type	Coordinates (Angstroms) X	Y	Z
1	46	0	-0.252015	-0.852948	-0.223393
2	6	0	1.345625	-1.309870	-2.740299
3	6	0	1.105867	-1.996251	-1.526100
4	6	0	1.285456	-1.970008	-3.969410
5	6	0	0.820727	-3.384011	-1.603672
6	6	0	1.005803	-3.337726	-4.010890
7	1	0	1.464971	-1.422358	-4.890366
8	6	0	0.786785	-4.048770	-2.823383
9	1	0	1.592383	-0.254268	-2.729069
10	1	0	0.966545	-3.853884	-4.966400
11	1	0	0.589154	-5.116436	-2.857991
12	1	0	0.663737	-3.926585	-0.677398
13	1	0	1.801365	-1.812452	-0.478953
14	8	0	-1.589054	-2.443107	-0.433767
15	6	0	-2.538729	-2.682294	0.423687
16	8	0	-3.309923	-3.634832	0.335899
17	6	0	-2.666262	-1.739752	1.649707
18	6	0	-4.109140	-1.631423	2.139856
19	1	0	-4.173725	-1.001466	3.032044
20	1	0	-4.776986	-1.232607	1.373977
21	1	0	-4.452289	-2.638035	2.389921
22	6	0	-1.743226	-2.254207	2.771114
23	1	0	-0.708627	-2.386513	2.441578
24	1	0	-1.773817	-1.585700	3.637263
25	1	0	-2.118138	-3.233551	3.085030
26	16	0	-2.019371	-0.031188	1.165310
27	6	0	-3.311863	0.619887	0.089336
28	6	0	-4.078967	1.677055	0.592949
29	6	0	-3.557758	0.105975	-1.190822
30	6	0	-5.100402	2.220418	-0.188595
31	1	0	-3.879393	2.069719	1.584659
32	6	0	-4.584328	0.654834	-1.960235
33	1	0	-2.958991	-0.712007	-1.574679
34	6	0	-5.354246	1.710131	-1.463074
35	1	0	-5.696134	3.039774	0.201749
36	1	0	-4.779864	0.255964	-2.950984
37	1	0	-6.150270	2.132957	-2.068472
38	15	0	1.035803	1.097383	0.049315
39	6	0	2.478490	1.457653	-1.046952
40	6	0	2.437349	2.466620	-2.023249
41	6	0	3.656142	0.704402	-0.886761
42	6	0	3.553746	2.717348	-2.824249
43	1	0	1.546867	3.068574	-2.158100
44	6	0	4.766296	0.964761	-1.689982

45	1	0	3.695074	-0.095923	-0.154749
46	6	0	4.719748	1.968820	-2.660163
47	1	0	3.508899	3.504394	-3.571138
48	1	0	5.669319	0.376151	-1.557603
49	1	0	5.586646	2.166399	-3.283783
50	6	0	-0.091032	2.532505	-0.207091
51	6	0	-0.839352	2.580430	-1.397455
52	6	0	-0.223349	3.573793	0.723045
53	6	0	-1.673556	3.665020	-1.665479
54	1	0	-0.770499	1.768411	-2.115744
55	6	0	-1.072954	4.650679	0.457053
56	1	0	0.333526	3.552579	1.652748
57	6	0	-1.791312	4.703288	-0.738012
58	1	0	-2.240018	3.691102	-2.591130
59	1	0	-1.167039	5.449774	1.186284
60	1	0	-2.445864	5.544906	-0.943672
61	6	0	1.733976	1.278982	1.737800
62	6	0	1.387460	0.378641	2.754922
63	6	0	2.624297	2.329125	2.024908
64	6	0	1.905580	0.536699	4.042621
65	1	0	0.741960	-0.464416	2.538432
66	6	0	3.136397	2.485192	3.312508
67	1	0	2.921113	3.022681	1.244570
68	6	0	2.775433	1.591170	4.324162
69	1	0	1.635189	-0.171632	4.819720
70	1	0	3.821093	3.301152	3.522982
71	1	0	3.179126	1.711543	5.325021
72	8	0	2.828632	-1.915248	0.503858
73	6	0	2.534076	-2.650623	1.527148
74	8	0	1.427117	-3.173318	1.746481
75	6	0	3.677603	-2.830354	2.526093
76	1	0	3.826657	-1.891282	3.070785
77	1	0	3.445014	-3.620741	3.242350
78	1	0	4.612136	-3.059138	2.006504

----

8. References

(1) Naksomboon, K.; Valderas, C.; Gómez-Martínez, M.; Álvarez-Casao, Y.; Fernández-Ibáñez, M. Á. *ACS Catal.* **2017**, *7*, 6342.

(2) Zhang, Z.; Wang, Z. *J. Org. Chem.* **2006**, *71*, 7485.

(3) Kubota, A.; Emmert, M. H.; Sanford, M. S. *Org. Lett.* **2012**, *14*, 1760.

(4) (a) Perego, L. A.; Grimaud, L.; Bellina, F. *Adv. Synth. Catal.* **2016**, *358*, 597; (b) Grushin, V. V.; Alper, H. *Organometallics* **1993**, *12*, 1890.

(5) Schröder, D. *Acc. Chem. Res.*, **2012**, *45*, 1521.

(6) Škríba, A.; Schulz. J.; Roithová, J. *Organometallics*, **2014**, *33*, 6868.

(7) Zins, E.-L.; Pepe, C.; Schröder, D. *J. Mass Spectrom*., **2010**, *45*, 1253; (b) Carpenter, J. E.; McNary, C. P.; Furin, A.; Sweeney A. F.; Armentrout, P. B. *J. Am. Soc. Mass Spectrom*., **2017**, *28*, 1876 (c) Rahrt, R.; Auth, T.; Demireva, M.; Armentrout P. B.; Koszinowski, K. *Anal. Chem*., **2019**, *91*, 11703.

(8) Gaussian 16, Revision A.03, Frisch, M. J.; Trucks, G. W.; Schlegel, H. B.; Scuseria, G. E.; Robb, M. A.; Cheeseman, J. R.; Scalmani, G.; Barone, V.; Petersson, G. A.; Nakatsuji, H.; Li, X.; Caricato, M.; Marenich, A. V.; Bloino, J.; Janesko, B. G.; Gomperts, R.; Mennucci, B.; Hratchian, H. P.; Ortiz, J. V.; Izmaylov, A. F.; Sonnenberg, J. L.; Williams-Young, D.; Ding, F.; Lipparini, F.; Egidi, F.; Goings, J.; Peng, B.; Petrone, A.; Henderson, T.; Ranasinghe, D.; Zakrzewski, V. G.; Gao, J.; Rega, N.; Zheng, G.; Liang, W.; Hada, M.; Ehara, M.; Toyota, K.; Fukuda, R.; Hasegawa, J.; Ishida, M.; Nakajima, T.; Honda, Y.; Kitao, O.; Nakai, H.; Vreven, T.; Throssell, K.; Montgomery, J. A., Jr.; Peralta, J. E.; Ogliaro, F.; Bearpark, M. J.; Heyd, J. J.; Brothers, E. N.; Kudin, K. N.; Staroverov, V. N.; Keith, T. A.; Kobayashi, R.; Normand, J.; Raghavachari, K.; Rendell, A. P.; Burant, J. C.; Iyengar, S. S.; Tomasi, J.; Cossi, M.; Millam, J. M.; Klene, M.; Adamo, C.; Cammi, R.; Ochterski, J. W.; Martin, R. L.; Morokuma, K.; Farkas, O.; Foresman, J. B.; Fox, D. J. Gaussian, Inc., Wallingford CT, **2016**.

(9) (a) Lee, C.; Yang. W.; Parr, R. G. *Phys. Rev. B*, **1988**, *37*, 785; (b) Becke, A. D. *J. Chem. Phys.*, **1993**, *98*, 5648; (c) Kohn, W.; Becke, A. D.; Parr, R. G. *J. Phys. Chem*., **1996**, *31*, 12974.

(10) (a) Dolg, M.; Wedig, U.; Stoll, H.; Preuss, H. *J. Chem. Phys*. **1987**, *86,* 866. (b) Andrae, D.; Haussermann, U.; Dolg, M.; Stoll, H.; Preuss, H. *Theor. Chim. Acta* **1990**, *77*, 123.

(11) Zhao, Y.; Truhlar, D. G. *Theor. Chem. Acc.*, **2008**, *120*, 215.

(12) (a) Furche, F.; Ahlrichs, R. *J. Chem. Phys.* **2003**, *119*, 12753; (b) Weigend, F;. Ahlrichs, R. *Phys. Chem. Chem. Phys.* **2005**, *7*, 3297.

(13) (a) Cancés, E.; Mennucci, B.; Tomasi, J. *J. Chem. Phys.*, **1997**, *107*, 3032; (b) Cossi, M.; Barone, V.; Mennucci, B.; Tomasi, J. *Chem. Phys. Lett.*, **1998**, *286*, 253; (c) Tomasi, J.; Mennucci, B.; Cancès, E. *J. Mol.Struc.: THEOCHEM*, **1999**, *464*, 211.
